# Putative effectors for prognosis in lung adenocarcinoma are ethnic and gender specific

**DOI:** 10.18632/oncotarget.4287

**Published:** 2015-06-22

**Authors:** Andrew Woolston, Nardnisa Sintupisut, Tzu-Pin Lu, Liang-Chuan Lai, Mong-Hsun Tsai, Eric Y. Chuang, Chen-Hsiang Yeang

**Affiliations:** ^1^ Institute of Statistical Science, Academia Sinica, Taipei, Taiwan; ^2^ Department of Public Health, National Taiwan University, Taipei, Taiwan; ^3^ Graduate Institute of Physiology, National Taiwan University, Taipei, Taiwan; ^4^ Institute of Biotechnology, National Taiwan University, Taipei, Taiwan; ^5^ Graduate Institute of Biomedical Electronics and Bioinformatics, National Taiwan University, Taipei, Taiwan

**Keywords:** lung adenocarcinoma, association module, ethnic specific, gender specific, east asian

## Abstract

Lung adenocarcinoma possesses distinct patterns of *EGFR/KRAS* mutations between East Asian and Western, male and female patients. However, beyond the well-known *EGFR/KRAS* distinction, gender and ethnic specific molecular aberrations and their effects on prognosis remain largely unexplored. Association modules capture the dependency of an effector molecular aberration and target gene expressions. We established association modules from the copy number variation (CNV), DNA methylation and mRNA expression data of a Taiwanese female cohort. The inferred modules were validated in four external datasets of East Asian and Caucasian patients by examining the coherence of the target gene expressions and their associations with prognostic outcomes. Modules 1 (*cis*-acting effects with chromosome 7 CNV) and 3 (DNA methylations of *UBIAD1* and *VAV1*) possessed significantly negative associations with survival times among two East Asian patient cohorts. Module 2 (*cis*-acting effects with chromosome 18 CNV) possessed significantly negative associations with survival times among the East Asian female subpopulation alone. By examining the genomic locations and functions of the target genes, we identified several putative effectors of the two *cis*-acting CNV modules: *RAC1*, *EGFR*, *CDK5* and *RALBP1*. Furthermore, module 3 targets were enriched with genes involved in cell proliferation and division and hence were consistent with the negative associations with survival times. We demonstrated that association modules in lung adenocarcinoma with significant links of prognostic outcomes were ethnic and/or gender specific. This discovery has profound implications in diagnosis and treatment of lung adenocarcinoma and echoes the fundamental principles of the personalized medicine paradigm.

## INTRODUCTION

It is commonly believed that major clinical phenotypes of cancer (e.g., tumorigenesis, malignancy, survival time, treatment response, metastasis) are caused by molecular aberrations - such as sequence mutations, copy number variations (CNV), epigenetic alterations - on a selected number of driver genes [1]. The list of driver genes expands substantially as new high-throughput technologies detect more rare molecular aberrations in larger patient cohorts [2]. Some scientists argue that the current list of leading driver genes is close to complete [3, 4].

Despite the rich documentation about driver genes, their specificity in terms of patient populations remains less well-known. Molecular aberrations of certain genes occur primarily on specific types of cancers in specific populations. Variations of driver molecular aberration landscapes across tumor types are widely appreciated and well-documented in cancer research [4, 5]. Characterization of drivers in race and gender specific subpopulations, in contrast, is relatively rare. Numerous studies demonstrated sex or ethnic specific prognostic outcomes in multiple cancer types. For instance, a survival advantage has been noted for younger females with cancer types such as hepatocellular carcinoma [6, 7], melanoma [8–10] and colorectal cancer [11, 12]. In contrast, a worse prognosis has been identified for female patients with bladder cancer [13, 14]. Prognostic differences between Caucasian and Asian men have also been reported in prostate cancer [15, 16]. However, the molecular aberrations underpinning these differences remain largely unclear. Characterization of drivers in race and gender specific subpopulations is a foundation of personalized medicine in oncology as it has strong implications in both diagnosis and treatments. Molecular biomarkers derived from one race/gender group may not be relevant in other populations [17, 18]. Furthermore, patients with distinct genetic backgrounds may yield drastically diverse responses to different drugs [19–21]. Identification of population-specific molecular aberration drivers bridges the gap between the demand of personalized medicine and existing knowledge and is thence highly impactful.

Lung cancer is the most commonly diagnosed and foremost cause of cancer related mortality [22]. Epidemiological studies have demonstrated significant gender and ethnic differences in trends amongst lung cancer patients. Tumors arising from the central airway compartment (predominantly small cell lung cancer and squamous cell carcinoma) are primarily associated with smoking. Tumors arising from the periphery airway compartment (predominantly adenocarcinoma) occur much more frequently in never-smokers and are attributed to poorly understood factors [23]. Lung cancers in non-smokers occur disproportionately more frequent amongst East Asian female populations [23, 24]. Moreover, lung adenocarcinoma exhibits mutually exclusive somatic mutations between patients from East Asian and European descent. East Asian patients possess predominantly *EGFR* mutations, whereas Western patients possess primarily *KRAS* mutations [25, 26].

The remarkable distinction of *EGFR/KRAS* mutations is already applied to screen recipients for the targeted drugs of tyrosine kinase inhibitors such as gefitinib [19, 27, 28]. Yet they are unlikely to be the only population-specific drivers of lung adenocarcinoma. A plethora of prior studies detected prognostic biomarkers of lung adenocarcinoma from high-throughput data of genome sequences [29, 30], transcriptome levels [31, 32], copy number variations [33, 34], epigenetic states [35, 36], and combinations of them [37, 38]. The majority of those studies restricted samples to certain geographic areas and/or gender groups. Without comparison and verification with other datasets, it is impossible to determine whether findings from single sources are universal, population-specific, or outliers rarely occurred elsewhere. Meta-analysis of lung adenocarcinoma has been pursued by a number of research groups [39–41]. However, those studies focused primarily on universal biomarkers common in all datasets, rather than population-specific patterns. This approach gives rise to robust findings but also fails to address the population-specific variations.

In our previous work [42], we developed an algorithm to identify association modules [43] of genes linking molecular aberrations on DNA with mRNA expressions from integrative datasets (copy number variations, DNA methylations, mRNA expressions), and employed this algorithm to the Glioblastoma multiforme data from The Cancer Genome Atlas (TCGA) [44]. In the present study, we applied the module-finding algorithm to a lung adenocarcinoma data of a cohort of female non-smoking Taiwanese patients, and examined the prognostic outcomes of the inferred modules in four additional datasets covering East Asian and Caucasian, male and female subpopulations. The novelty of this work is to investigate and demonstrate ethnic and gender specific properties of the inferred modules. Strikingly, three modules – *cis*-acting effects with chromosomes 7 and 18 CNVs, and methylations with *UBIAD1* and *VAV1* – demonstrated strong associations with prognosis amongst the East Asian or the East Asian female subpopulation. In the specified subpopulation, patients possessing high average expression levels in each association module had significantly shorter survival times than patients possessing low average expression levels. For the module with *cis*-acting effects on chromosome 18, the associations of module members with survival times were significant in East Asian females, but insignificant among other subpopulations (East Asian males, Western males and females). In contrast, for modules with *cis*-acting effects with chromosome 7 and methylations with *UBIAD1* and *VAV1*, these demonstrated strong prognostic effects amongst the East Asian subpopulations. Extending the validation methods of our previous study, we utilized partial least squares (PLS) to analyze dependencies between the inferred association modules. Co-citation and pathway enrichment methods were also included in the analysis to identify likely putative effector genes and their possible role in related biological mechanisms. Beyond the well-known *EGFR*, the two *cis*-acting modules also harbor other likely effectors such as *RAC1* (chromosome 7), *CDK5* (chromosome 7), and *RALBP1* (chromosome 18).

## RESULTS

### Association modules

Cancer cells harbor a large number of molecular aberrations such as CNV, DNA methylation and sequence mutations. Some aberrations may dysregulate gene expressions, which in turn drive the malignancy of tumors. Those mechanistic relations between molecular aberrations and gene expressions can be manifested on the statistical associations of their measured data. Previously, we encapsulated those statistical associations as modules [42, 43]. An association module consists of three components: (i) an observed effector molecular aberration on DNA; (ii) the downstream target genes with expression profiles associated with the effector molecular aberration; and (iii) regulators (transcription factors or signalling proteins) that mediate the effects between effector molecular aberrations and target gene expressions. For the data types available in this study, the association modules generated can be described by three distinct types:

1. ***Cis*-acting effects with CNVs of chromosomes** The segment CNV of a chromosome is positively associated with the expressions of target genes on the same chromosomal segment location.
2. ***Trans*-acting effects with CNVs of chromosomes** The segment CNV of a chromosome exhibits *cis*-acting effects with intermediate regulators, and both the chromosome segment CNV and regulator expressions have either positive or negative associations with the expressions of target genes on other chromosomes.
3. **Effects with DNA methylations** The coherent DNA methylation states of a collection of genes are negatively associated with the expressions of themselves and other target genes.

**Figure 1:**
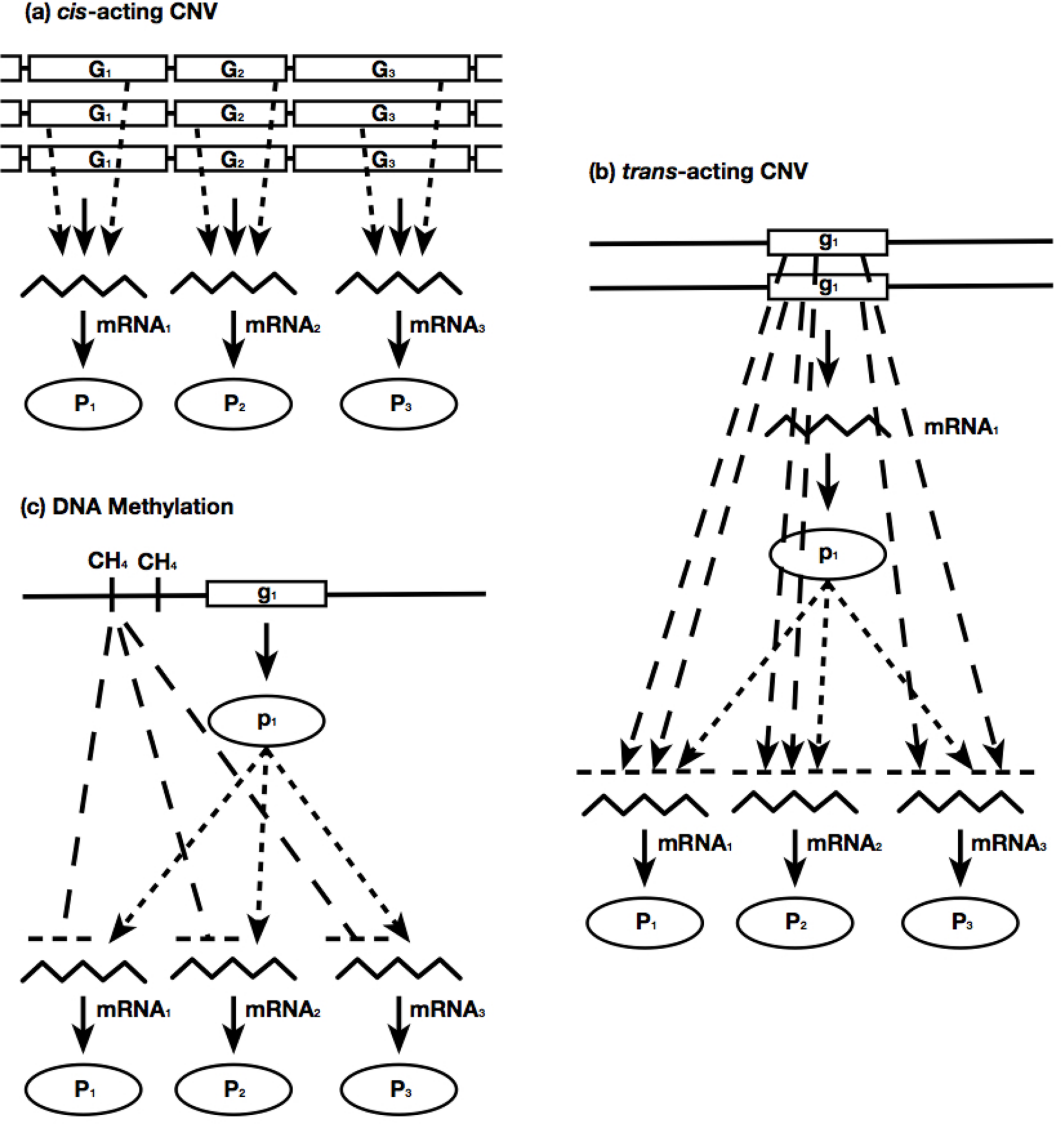
Three types of association module **a.** *Cis*-acting effects with CNVs of chromosomes, **b.** *Trans*-acting effects with CNVs of chromosomes; and **c.** Effects with DNA methylations. Solid lines: information flows following the central dogma (DNA → mRNA → protein). Dotted lines: regulatory links from regulators to their targets on other chromosomal locations. Dashed lines: associations between observed aberrations and mRNA gene expressions. Arrowheads indicate positive associations and bar-ends indicate negative associations. The figure is adapted from [42].

A summary of the assumptions underlying these modules is illustrated in Fig. 1. We employed a layered approach to prioritize associations in terms of their mechanistic certainty, incrementally constructed the logistic regression model of each gene expression profile, and grouped the genes sharing common effectors to form association modules. The procedures of the module finding algorithm are described in Materials and Methods and S1 Text.

### Datasets

Tissue specimens were collected from non-smoking female lung cancer patients admitted to National Taiwan University (NTU) Hospital and Taichung Veterans General Hospital (details of data collection have been described previously [45]). A total of 60 tumor and adjacent normal lung tissue specimen pairs were interrogated using Affymetrix U133plus2.0 mRNA expression microarrays, Affymetrix SNP6.0 microarrays, and Illumina Infinium. A further 32 pairs of lung adenocarcinoma samples were selected with both DNA and mRNA expression data available. We pre-processed those integrative datasets and converted them into standard formats (see Materials and Methods for details), and employed the module finding algorithm to the processed data. Validation of the inferred modules was performed on the mRNA and survival data from two lung adenocarcinoma datasets of East Asian cohorts (Japanese [46] and Korean [47]) and two datasets of US Caucasian populations (US_1_ [48] and US_2_ [49]). Table 1 summarizes the information of the five datasets. In the two US datasets only lung adenocarcinoma samples from Caucasian patients were extracted. All data except the training set of the Taiwanese cohort have clinical information available to assess the prognostic value of the association modules.

**Table 1:** East Asian and White Caucasian datasets of lung adenocarcinoma

Key	Ethnicity	Subjects (Male/Female)	Reference
Taiwan	East Asian	32 (0/32)	[45]
Japan	East Asian	117 (60/57)	[46]
Korea	East Asian	63 (34/29)	[47]
US1	White Caucasian	244 (104/140)	[48]
US2	White Caucasian	294 (127/167)	[49]

A description of the data used in the study. The Taiwan mRNA, CNV and methylation integrative data were used to build the association modules. Validation of the modules was performed on mRNA and clinical data from two East Asian cohorts (Japan and Korea) and two datasets of US Caucasian populations (US_1_ and US_2_).

### Discovery and validation of association modules

The module finding algorithm [42, 43] was applied to the Taiwanese dataset. A total of 44 association modules were generated: 23 modules with *cis*-acting effects of chromosomes' CNVs, 15 modules with *trans*-acting effects of chromosomes' CNVs, and 6 modules with DNA methylations as effectors. A complete list of association modules is provided in S1 Table. The inferred modules were passed through two validation tests on two East Asian female datasets to examine their biological and prognostic relevance. First, we checked whether the targets in each module retained coherent expression profiles in both external datasets. Expression profile coherence of an association module was gauged by statistical deviation of its pairwise correlation coefficient distribution from a background distribution derived from a random collection of genes (S2 Table (a)). Second, we checked whether the target gene expression profiles in each module possessed prognostic power. Prognostic power of an association module was assessed by two indicators. We evaluated the Cox regression coefficients of target gene expression profiles and calculated the Kolmogorov-Smirnov (KS) statistical significance of their distribution relative to a background distribution derived from a random collection of genes (S2 Table (b)). To reduce the effect of module size to the KS statistical significance, we further collapsed the expression profiles of multiple target genes into one median expression profile and evaluated the log-rank *p*-values of the Kaplan-Meier curves derived from the aggregate biomarker (S2 Table (c)). Validation procedures are elaborated in Materials and Methods.

**Figure 2:**
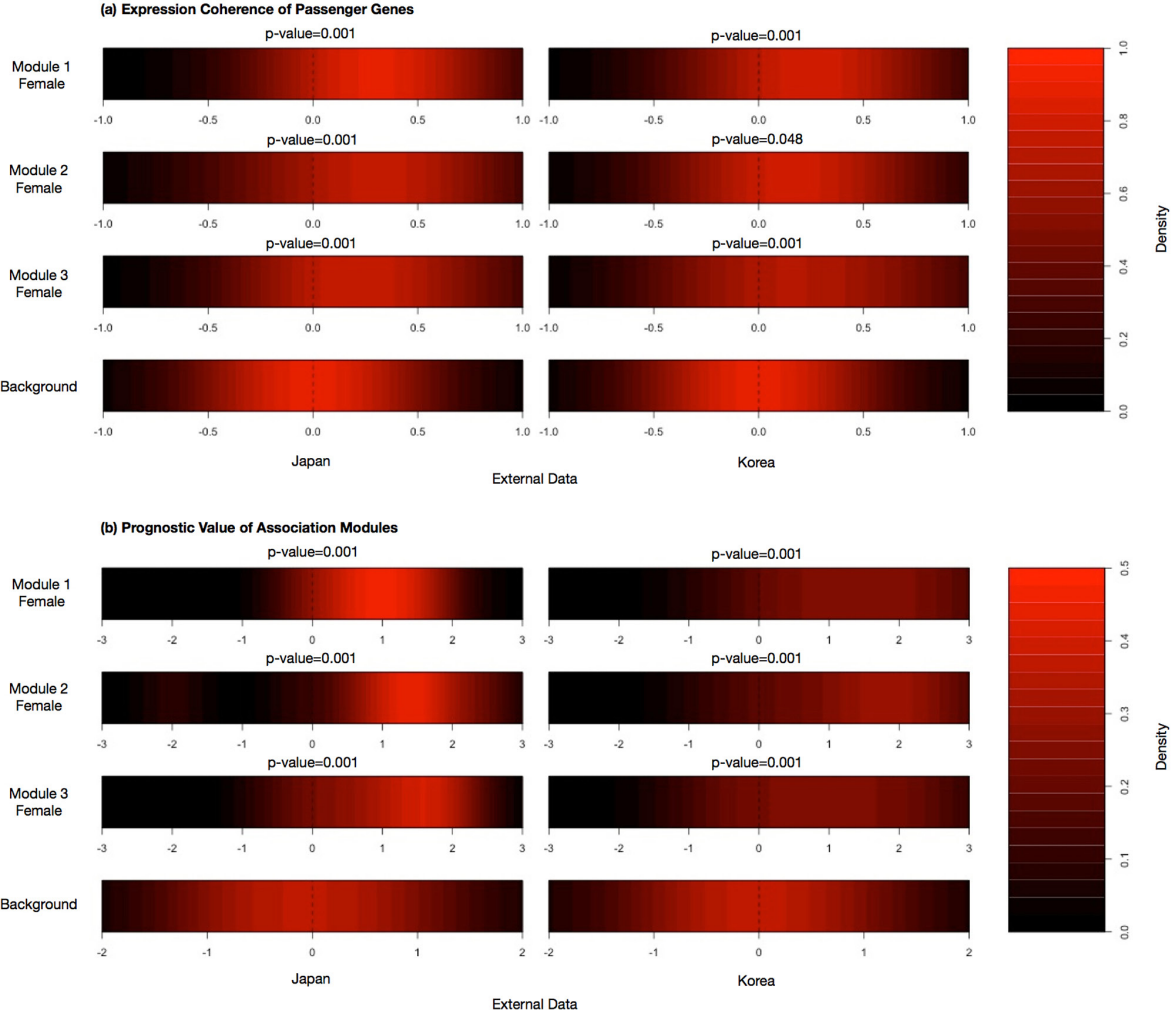
Validation tests to examine the target gene coherence of genes and prognostic power of the modules **a.** Heatmaps illustrating the distributions of correlation coefficients among target gene expressions of female East Asian datasets. **b.** Heatmaps illustrating the distributions of Cox coefficients of target genes for female East Asian datasets. A distribution of all genes within the external data is included to provide a background comparison to compute the Kolmogorov-Smirnov statistic.

Three association modules passed the validation tests: module 1 comprises *cis*-acting effects of chromosome 7 CNV, module 2 comprises *cis*-acting effects of chromosome 18 CNV, and module 3 comprises DNA methylation of *UBIAD1* and *VAV1* as effectors. Fig. 2 displays expression coherence and prognostic power of the three modules on two East Asian female datasets. Both expression correlation coefficients and Cox regression coefficients have significantly positive deviations from the background distributions. The false discovery rate (FDR) of randomized modules passing all the validation tests is < 0.002 (see Materials and Methods and S1 Text about FDR evaluation procedures).

### Inferred association modules are more specific within the East Asian female population

Since the three modules were inferred from and validated on the datasets of East Asian female patients, it is unclear whether they were universal to all the patient populations or specific to the East Asian female subpopulation alone. To answer this question, we extended validation by including two additional datasets of Caucasian patients (US_1_ and US_2_) and further divided each dataset by gender. Accordingly there were four subpopulations: East Asian females, East Asian males, Caucasian females and Caucasian males. We again analyzed Kaplan-Meier curves for each inferred module to study time-to-event data. Patients were assigned to high and low expression groups if the median gene expression for the selected module was higher or lower than the average module expression respectively. All three modules demonstrated strong prognostic effects amongst female East Asian subpopulations and one module was both gender and ethnic specific.

### Modules 1 and 3 show prognostic power in East Asian populations

**Figure 3:**
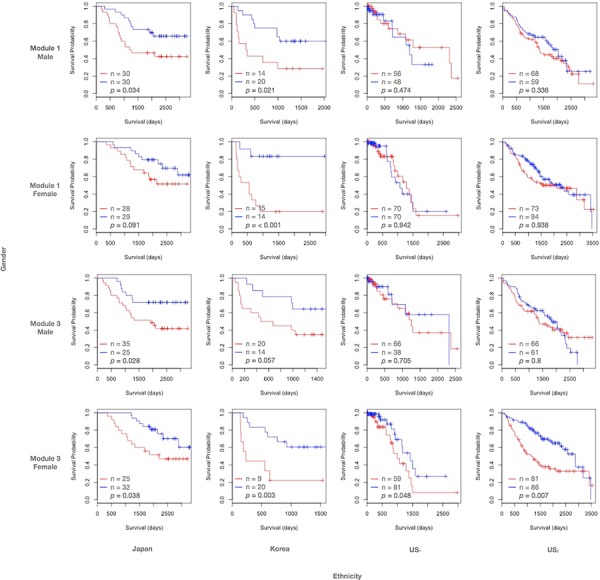
Kaplan-Meier curves of modules 1 and 3 Kaplan-Meier survival curves of patients divided by median gene expression amongst target genes in inferred modules. A red line indicates the survival curve of the patient group with high median expression levels in the target genes. A blue line indicates the survival curve of the patient group with low median expression levels in the target genes. Tick marks indicate censored data points; *p*-values are determined by log-rank tests. The size of each patient group and the log-rank *p*-value are reported.

Expression coherence of modules 1 and 3 was significant in all the four subpopulations (S1 Fig. (a) and (c)). In contrast, their prognostic outcomes were highly specific in certain subpopulations. Fig. 3 displays the Kaplan-Meier curves for modules 1 and 3, with patients assigned to low and high expression groups based on median gene expression. For module 1 we observed that the associations of module members with survival times was significant for East Asian (columns 1 and 2), but not Caucasian (columns 3 and 4) subpopulations. In both Japanese and Korean cohorts, the Kaplan-Meier curves indicated that a high expression of target genes had a negative effect on the survival prospects of the subject. The log-rank *p*-value of module 1 in the Japanese female data is less significant (*p* = 0.091), but still substantially lower than the two US counterpart datasets (*p* = 0.942 and 0.938 respectively). Furthermore, module 1 exhibited significant prognostic outcomes in both East Asian males (rows 1 and 3) and females (rows 2 and 4), suggesting ethnic rather than gender specificity. In contrast, module 3 demonstrated a similar significance for East Asian subpopulations, but was also significant for the female Caucasian cohorts.

### Module 2 is gender and ethnic specific

**Figure 4:**
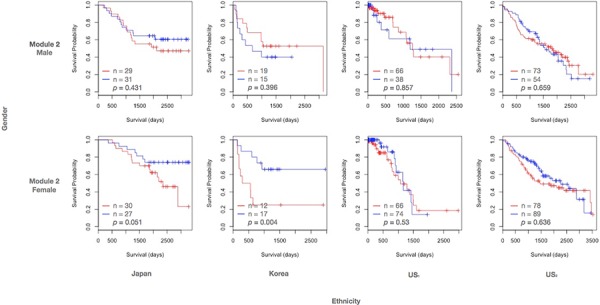
Kaplan-Meier curves of module 2 The legend follows Fig. 3.

Expression coherence of module 2 was also significant in all the four subpopulations (S1 Fig. (b)). Furthermore, module 2 exhibits significant prognostic outcomes in the East Asian female subpopulation alone. Fig. 4 displays the Kaplan-Meier curves for module 2, with patients assigned to low and high expression groups based on median gene expression. The log-rank *p*-values were significant only among the females of the Japanese and Korean datasets. Like modules 1 and 3, a high expression of target genes has a negative effect on the survival time. The Cox coefficient distributions achieved significantly positive deviation in East Asian female and Japanese male subpopulations (S2 Fig. (b)). Although the distribution was significant in Japanese males (*p* = 0.003), this trend was further enhanced in Japanese females (*p* = 0.001).

### Dependency between association modules

As modules 1 and 3 demonstrated a similar distribution of Cox coefficients in our tests of prognostic power (S2 Fig. (a) and (c)), we examined the possibility that chromosome 7 CNV is a candidate effector for the target gene expressions observed in module 3. A partial least squares (PLS) regression [50, 51] was employed to assess the association between target genes of modules 1 and 3.

PLS is useful for assessing the module members in this study, as we may treat the targets of two modules as X and Y blocks of genes and build a PLS model between them. This allows us to calculate an *R^2^*-type measure for the first few components in each block, that can represent a variance explained between association modules. The procedures of computing PLS are described in Materials and Methods. A correlation circle can be useful in demonstrating variable loadings on the first two PLS components. This plot demonstrates the associations between module targets, by assessing their proximity with one another. The cosine of the angles between gene locations in the plot will indicate the correlation between module members projected onto these two PLS components. In addition, the orthogonal projection of the module members onto the component axes will demonstrate the respective loading on each component.

**Figure 5:**
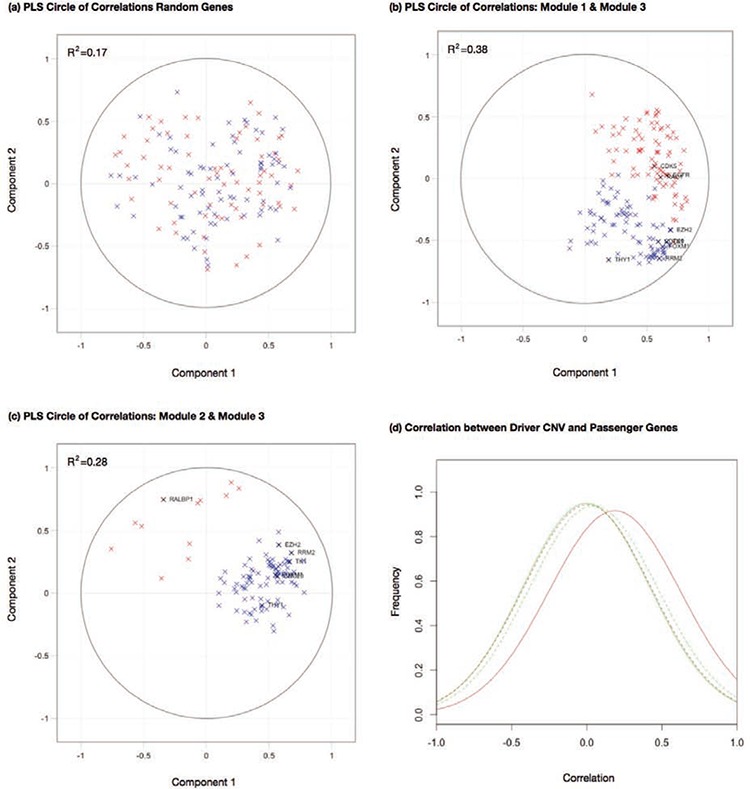
Partial least squares to investigate an association between modules in the Taiwan data **a.** A correlation circle displaying the first two component loadings for two sets of randomly selected genes **b.** A correlation circle displaying the first two component loadings for target genes in modules 1 (shown in red) and 3 (shown in blue), **c.** A correlation circle displaying the first two component loadings for target genes in modules 2 (shown in red) and 3 (shown in blue). **d.** Distributions of correlation coefficients between module effectors and target genes. Solid red line: correlation coefficients between module 1 effector of chromosome 7p CNV and module 3 target gene expressions. Solid green line: correlation coefficients between module 1 effector of chromosome 7q CNV and module 3 target gene expressions. Dashed red line: correlation coefficients between module 2 effector of chromosome 18p CNV and module 3 target gene expressions. Dashed green line: correlation coefficients between module 2 effector of chromosome 18q CNV and module 3 target gene expressions.

Two sets of randomly selected genes confer no correlation on their PLS component projections (Fig. 5(a)). The correlation circles for modules 1 and 3 in Fig. 5(b) demonstrated the projection of their target genes on the first two PLS components in the Taiwanese dataset. The two clusters of genes occupied restricted regions separated by an angle less than *π*/2, indicating positive correlations between them. Positive correlations between target genes of modules 1 and 3 were pronounced by comparing Fig. 5(b) with the correlation circle plot between modules 2 and 3 (Fig. 5(c)). In the latter case the two clusters of genes spread over a wide range of angles and possessed a much smaller *R^2^* for the first two components relative to the former case (0.38 versus 0.28 respectively). Similar trends were observed in Japanese and Korean datasets (S3 Fig.).

The effectors of modules 1 and 3 are chromosome 7 CNV and DNA methylation of UBIAD1 and VAV1 respectively. Strong correlations of their target genes suggest that module 3 targets might be affected by chromosome 7 CNV. To further verify this hypothesis, we calculated the distributions of correlation coefficients between module 3 target gene expressions and the segment CNVs of chromosome 7p and 7q in the Taiwanese data (Fig. 5(d)). The correlations between chromosome 7p CNV and module 3 target gene expressions have a significantly positive deviation (solid red line) relative to the background correlation coefficient distributions between chromosome 18 p and q arm CNVs and module 3 target gene expressions (dashed red and green lines respectively). In contrast, the correlations between chromosome 7q CNV and module 3 target gene expressions are close to the background distributions (solid green line). The results suggest that the effector genes for module 3 targets are likely located on chromosome 7p.

### Identification of putative effector genes on *cis*-acting association modules

**Figure 6:**
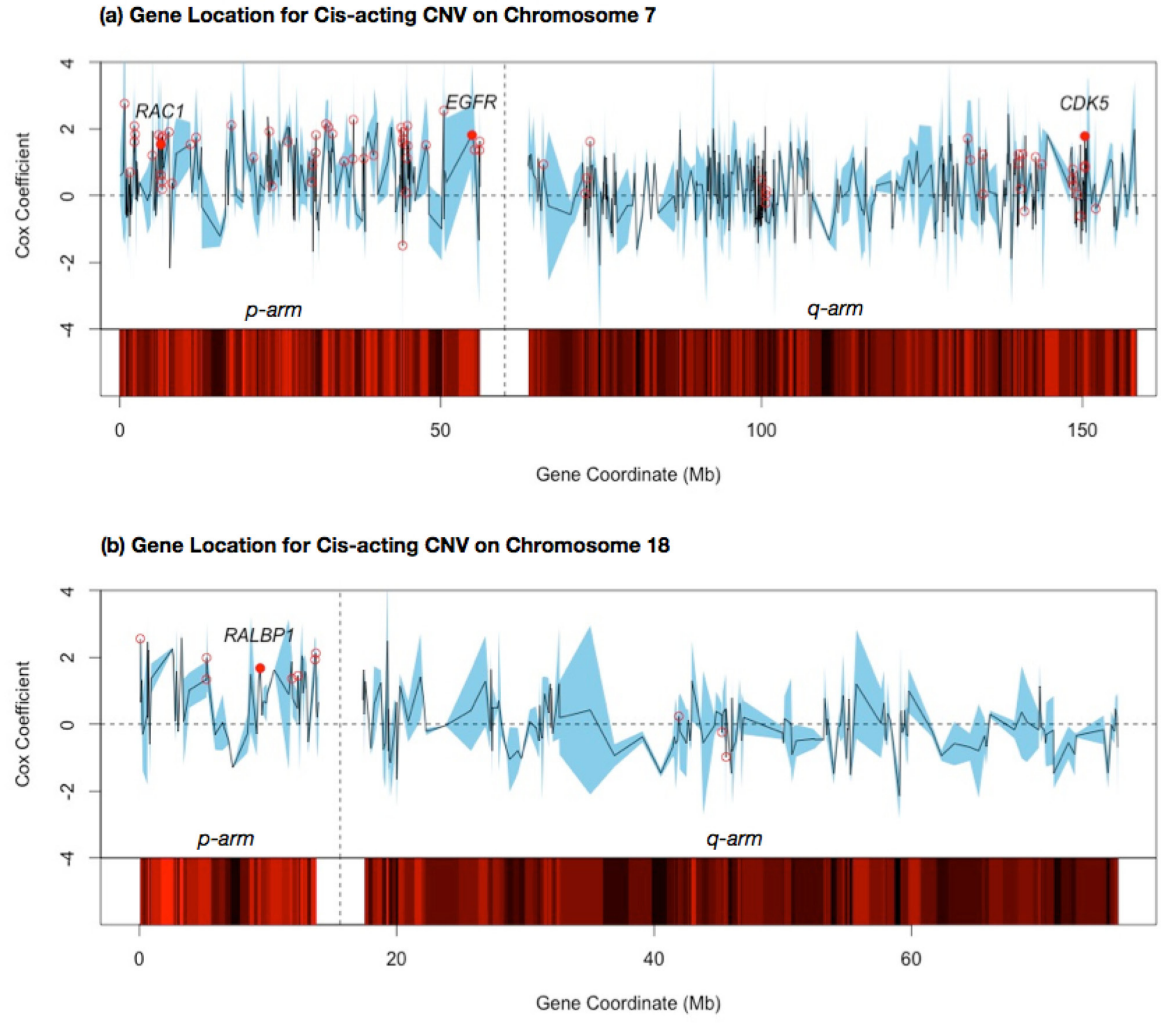
Cox regression coefficients of target genes within selected *cis*-acting CNV modules mapped by the relative locations on the chromosome The average of the standardized Cox coefficients for the two East Asian female datasets is shown by the black line, with a confidence interval for the two datasets shown by the blue band. Each target gene in the association module is flagged by a hollow red dot. The names of genes with high-ranking PubMed citations to lung cancer key terms are highlighted (see Materials and Methods), with the mean Cox coefficient shown with a solid red dot. The p and q arms of each chromosome are separated by blank columns; **a.** shows the gene locations for *cis*-acting CNV on chromosome 7; **b.** shows the gene locations for *cis*-acting CNV on chromosome 18.

Prognostic effects of a *cis*-acting CNV module are likely caused by a few effector genes on the designated chromosome. Expression level variations of those genes may modulate activities of specific functions and eventually affect survival times. Other genes in the vicinity may possess similar CNV and expression profiles as the effectors, thus exhibit a strong correlation with survival times despite the lack of mechanistic links. To identify putative effector genes on *cis*-acting CNV modules, we searched co-citations of the keyword *lung cancer* with all the module members and examined the distributions of their chromosomal locations. Fig. 6 shows the locations of all genes on chromosomes 7 and 18 and their Cox regression coefficients. The Cox coefficients have been standardized to allow for a direct comparison across external East Asian female validation datasets. Members of association modules are flagged by red circles. Intriguingly, members of both modules 1 and 3 were clustered in a few regions of the corresponding chromosomes. Module 1 targets were located primarily on chromosome 7p and a smaller region (130–150 Mb) of chromosome 7q. Module 2 targets were located on chromosome 18p. Generally, module members possessed higher Cox regression coefficients than other genes on the same chromosomes. Yet there was no consecutive region with consistently high Cox regression coefficients among all constituent genes.

To solicit putative effector genes in the *cis*-acting CNV modules, we counted the number of co-citations with the keyword *lung cancer* and each target gene and retained the top ranking 5% of genes as described in Materials and Methods. Results of the citation count are presented in S3 Table (a). Four genes were identified in the co-citation search as putative effectors of modules 1 and 3.

- ***RAC1*** (chromosome 7p): A signalling G protein member of the Rho family of GTPases, involved in cancer cell proliferation and invasion. The effect on cell motility may result in epithelial-mesenchymal transition, driving tumor metastasis in lung adenocarcinoma for cancer progression and drug-resistant tumor relapse [52, 53].
- ***EGFR*** (chromosome 7p): A cell surface receptor and member of the ErbB family of receptors. Overexpression of *EGFR* by mutation, amplifications or misregulations is considered a key factor in the uncontrolled cell division of a number of cancer types [54]. *EGFR* inhibitors, such as gefitinib and erlotinib, have been developed primarily to target lung adenocarcinoma [19, 27].
- ***CDK5*** (chromosome 7q): Codes an enzyme involved in sensory signalling. *CDK5* plays a role in many neurodegenerative diseases and cancers by phosphorylating the actin regulatory protein caldesmon [55]. Amplification of *CDK5* has been found to play an important role in the migration and invasion of lung adenocarcinoma cancer cells.
- ***RALBP1*** (chromosome 18p): A protein that is commonly overexpressed in malignant cells, preventing apoptosis in selected cancer cells [52]. *RALBP1* is a major transporter of doxorubicin, a drug used in cancer chemotherapy, and can potentially contribute to multi-drug resistance [56].

The effector analysis was repeated on the external male East Asian validation datasets and illustrated in S4 Fig. Whilst positive Cox coefficients are again observed amongst these putative effector genes, we note that the coefficients of *EGFR* and *RALBP1* in particular are not as strong when compared with the female subpopulations.

Whilst citation count provides a useful quantitative measure of a target genes relevance to lung adenocarcinoma, it is potentially biased against recently discovered cancer genes. To relieve this problem, we adopted an alternative approach by using the NCBI OMIM database [57] to identify cancer-related genes, and to search for their overlap with the target genes of inferred modules. Results of the OMIM analysis are presented in S3 Table (b). A total of 100 genes of the inferred modules were present in the OMIM database, including *RAC1*, *EGFR* and *CDK5*.

### Pathway enrichment of target genes

A pathway enrichment was performed using QIAGEN's Ingenuity pathway analysis (IPA) (http://www.qiagen.com/ingenuity) [58]. Complete results of the pathway analysis for all modules are included in S4 Table. A total of 8 pathways were found significant for the target genes in module 1 by the criteria defined in Materials and Methods. The pathways of module 1 are related to less specific processes, such as DNA transcription and translation control. In comparison, no pathways reached significance for module 2. This is likely due to the small size of the module (i.e. 12 genes) and the location restriction of target genes to chromosome 18 only.

Module 3 is the only significant association module containing target genes not restricted to a single chromosome. This reflects the nature of the majority of pathways in the IPA database. Two of the highest scoring cancer pathways in module 3 were ‘uterine serous papillary cancer’ (*p* = 3.82 × 10^−13^) and ‘mitosis of cervical cancer cell lines’ (*p* = 1.93 × 10^−7^). This appears relevant to the current study, as both are cancers predominantly affecting female populations. Common to both of these pathways were genes such as *CDC20* and *PTTG1*. These genes were also significant in the PubMed search of key terms (see Materials and Methods and S3 Table), along with other members of the ‘uterine serous papillary cancer’ pathway such as *FOXM1*, *TK1*, *CCNA2* and UBE2C. Human Papillomavirus (HPV) has previously been linked to lung cancer in Taiwanese female non-smokers [59], and so this finding may hold some significance for module 3.

## DISCUSSION

In this study, we demonstrated that the dominant associations linking DNA molecular aberrations, mRNA expressions and prognosis in lung adenocarcinoma were population specific. Target gene expressions of chromosome 7 *cis*-acting CNV (module 1) and *UBIAD1* and *VAV1* methylations (module 3) had negative effects on survival times among East Asian patients. Furthermore, target gene expressions of chromosome 18 *cis*-acting CNV (module 2) had negative effects on survival times among East Asian female patients alone. Gender and ethnic specificity of biomarkers has profound implications in the diagnosis and treatment of cancer. Patients with different ethnic and gender backgrounds should be diagnosed with different molecular biomarkers and receive different treatments targeting distinct molecular aberrations. This mode of operations will constitute the foundation of personalized medicine in the post-genomic era.

Our use of the term “putative effectors” has a weaker connotation than the canonical definition of “driver genes” in cancer research. A putative effector is identified by statistical associations between DNA molecular aberrations and transcriptional profiles, and further validated by information from external datasets as well as literature co-citation and functional annotations. In contrast, the driver function of a gene cannot be affirmed without explicit intervention such as mutagenesis, RNA silencing or genome editing. Nevertheless, the list of putative effectors provides useful candidates for further experimental validation.

The salient associations of chromosome 7 *cis*-acting CNV (module 1) with prognosis likely reflects the effect of *EGFR*. *EGFR* mutations occur predominantly among East Asian lung cancer patients [25, 26]. Here we demonstrated that beyond mutations, copy number variations of *EGFR* (and chromosome 7p in general) also likely contributed to poor prognosis in East Asian cohorts.

The effectors of module 3 – DNA methylations of *UBIAD1* and *VAV1* – are less well-known in lung adenocarcinoma. Unlike *cis*-acting CNV modules, associations with DNA methylations are more difficult to verify since DNA methylations are not probed in all external datasets, and their proxies – mRNA expressions of the methylated genes – rely on a few genes. Coincidentally, we discovered that the target genes of module 3 were correlated with both effectors and targets of module 1. Such dependency suggests that module 3 may also be a *trans*-acting module of chromosome 7p CNV. Although no module 3 targets are located on chromosome 7p, they can be co-regulated by a common effector gene on chromosome 7p and hence exhibit strong dependency with module 1. *EGFR* is a likely candidate for the effector as (1) it is located on chromosome 7p, (2) it is an oncogene with a negative effect on survival duration, and (3) its molecular aberration is biased in East Asian cohorts.

Despite the strong association between chromosome 7p CNV and module 3 target gene expressions, the *trans*-acting module of chromosome 7 CNV inferred from the Taiwanese data (S1 Table) did not pass the validation tests nor was overlapped with module 3. As shown in Fig. 5(d), many of chromosome 7p CNV and module 3 target gene expression associations were significantly higher than the background but still weaker than the correlation coefficient threshold (0.5) in the module finding algorithm. Thus the *trans*-acting module of chromosome 7 CNV was not reported in our study.

Unlike chromosome 7 and *EGFR*, prognostic associations of chromosome 18 (module 2) and its gender and ethnic specificity are novel and striking. The negative effect on survival is manifested in East Asian female patients alone. *RALBP1* is the only candidate effector with relatively extensive reports in lung cancer. The function of *RALBP1* as a molecular transporter may account for its links to drug resistance and hence poor prognosis.

It is difficult to establish mechanistic links from the biomarker levels to prognostic outcomes as the latter are determined by many genetic and environmental factors. Generally, the associations with most members of *cis*-acting CNV modules (modules 1 and 2) are likely caused by a few effector genes in the vicinity. In contrast, the associations with members of *trans*-acting modules (module 3) are possibly on the genes mediating the effector molecular aberrations (chromosome 7p CNV or *UBIAD1* and *VAV1* methylations) and the prognostic outcomes. Indeed, the few putative effector genes of modules 1 and 2 (*RAC1*, *EGFR*, *CDK5*, *RALBP1*) and many target genes in module 3 are involved in cell division and proliferation, anti-apoptosis, and molecular transport. High activities in those processes will enhance malignancy and drug resistance and thus lead to poor prognostic outcomes. However, although the directions of prognostic associations match the functions of those genes, it remains a puzzle to explain why those apparently generic processes are gender and ethnic specific.

An equally important puzzle is the cause of gender and ethnic specificity of molecular aberrations. The majority of the molecular aberrations observed in cancer genomic data are somatic - arising after birth rather than being inherited. Why would certain types of molecular aberrations (e.g., *EGFR* mutations or amplifications) arise more frequently in a specific population (e.g., East Asian female patients) even though they are not inherited? Understanding the causes of population-specific bias of somatic aberrations will be a milestone to the research of tumor genome evolution.

Enrichment of pathways involved in papillary and cervical cancers in module 3 members is intriguing. Given the strong bias in East Asian female populations and correlations with other female-specific cancers, lung adenocarcinoma with *EGFR* aberrations has very strong links with female patients of papillary and cervical cancers.

The association modules were generated on a Taiwanese training dataset, with significance determined on two external East Asian validation datasets. The inferred modules would ideally have been verified on a third East Asian dataset independent of module discovery. However, the current study was limited by the lack of a suitable external candidate data. Nevertheless, the inclusion of East Asian and Caucasian external datasets allowed for intriguing insights to be made on the population specificity of the inferred association modules.

One counter-argument against the genetic explanation for the population-specific difference is their smoking behaviors. Male lung cancer patients have a disproportionately higher smoking rate than female counterparts [60]. Thus the male-female difference may be attributed to smoking behaviors rather than genetics and sex-specific physiology. Due the lack of smoking status reports in the external datasets, we can neither rule out nor confirm this confounding factor. A larger dataset(s) covering cases of all possible combinations of gender, ethnicity and smoking behaviors is needed in order to resolve this contention.

Likewise, the present work cannot determine whether genetic or environmental differences are the causes of ethnic specificity. An ideal study to answer this question is to collect patients of European and East Asian descent in the same locales and investigate whether their samples exhibit the same ethnic-specific trends. The datasets appeared in present work do not have sufficient ethnic diversity for the proposed study. The TCGA lung adenocarcinoma data contains only 12 Asian samples, whereas all the East Asian datasets contain samples from mono-ethnic origins.

Lung adenocarcinoma is not the only cancer exhibiting population specificity. The results in this study add to this growing body of research and further highlight gender and ethnicity as predominant risk factors in predicting the survival outcomes of patients in a range of cancer types. Identification of other cancer types demonstrating specific molecular aberrations and prognostic effects in gender, race and other attributes is an important extension of the present study.

## MATERIALS AND METHODS

### Data sources and processing

Table 1 summarizes the information of the five datasets analyzed in the present study. The training set of 32 Taiwanese female lung adenocarcinoma patients consists of the data of transcriptomes (Affymetrix U133plus2.0 microarrays), CNV (Affymetrix SNP6.0 microarrays), and DNA methylations (Illumina Infinium). In each patient the measurements from the tumor tissue and the adjacent normal tissue were provided. We calculated the tumor/normal ratios of each data type and used them in constructing association modules. However, survival data in the training set was not included in our analysis due to the shortage of events: 28 of the 32 subjects were censored.

Validation data were identified and downloaded from open-source locations. These included the Gene Expression Omnibus (GEO, http://www.ncbi.nlm.nih.gov/geo/), The Cancer Genome Atlas (TCGA, https://tcga-data.nci.nih.gov/tcga/) and caArray (https://array.nci.nih.gov/caarray/home.action). The four validation sets consist of transcriptomic and survival data. The Japanese dataset contains the transcriptomic data of Agilent G4112F microarrays on 117 patients. The Korean dataset contains the transcriptomic data of Affymetrix U133plus2.0 microarrays on 63 patients. The US_1_ source is the TCGA lung adenocarcinoma (LUAD) dataset. We selected the transcriptomic data of Illumina RNAseq on 244 Caucasian patients. The US_2_ data also includes patients from diverse ethnic backgrounds. We selected the transcriptomic data of Affymetrix U133A on 294 Caucasian patients.

Probe information for the specific microarray platform was used to assign gene information (i.e. gene name, chromosome number, genomic location) to each probe level data. For CNV and mRNA expression data, we normalized the measurements into a compatible scale. For each probe in a microarray or each gene in an RNAseq assay, we converted the vector of observed data over a patient cohort into the vector of cumulative distribution function (CDF) values. The normalized values ranged between 0 and 1. DNA methylation data ranged in [0, 1] thus did not require normalization. The normalized value of a gene in a subject was the median over its probe values. Furthermore, the CDF value was converted into a probability vector of trinary states (up, down regulation and no change):

$$\begin{array}{l} P\left(x=1|y\right)=\int_{1}^{\infty }\frac{y}{1-\log y}\\ P\left(x=-1|y\right)=\int_{1}^{\infty }\frac{1-y}{1-\log\left(1-y\right)}\\ P\left(x=0|y\right)=1-P\left(x=1|y\right)-P\left(x=-1|y\right) \end{array}$$

where *y* denotes the CDF value and *x* the hidden discrete variable of the expression (CNV) state. Derivation of the formulation is described in [42]. Following the CDF transformation, we split the patients of each transcriptome data by gender to generate separate datasets for the Japan, Korea and US studies.

The elementary subunits of CNV data are segments bounded by amplification and deletion events. To identify segmentation regions, the raw CNV data were visualized using heatmaps. We observed that CNV data were mostly coherent within each chromosomal arm. To simplify computation, a decision was therefore made to use chromosomal arms as natural segmentation boundaries. We aggregated the measurements of probes on each chromosomal arm and used the median over the corresponding probe values as the proxy CNV value of a chromosome segment.

### Purity and ploidy of the tumors

We employed the ABSOLUTE algorithm [61] to estimate both ploidy and purity of samples in the training set. The inferred results are reported in S5 Table. The inferred purity indicates most samples retain a significant fraction of cancer DNA. Therefore, the association modules reflect information in cancer genomes. The inferred ploidy indicates many cancer samples undergo copy number amplifications in most spots in the genomes. The fluctuations of CNVs provide a necessary condition to build associations between CNV and mRNA data.

### Module finding algorithm

We established a logistic regression model to fit the mRNA expression profile of each gene in terms of CNV and DNA methylation data. Denote *y* the expression of a gene and x the molecular aberrations that explain *y*. The conditional probability is

$$P\left(y|x\right)=\frac{1}{Z\left(x\right)}e^{\sum_{i}\lambda_{i}f_{i}\left(x\right)},\lambda_{i}\geq 0,i.$$

where *f_i_*(x)'s are scalar feature functions specifying the relations of x and *y*. λ_i_'s are non-negative parameters, and *Z(*x) is the partition function that normalizes the conditional probabilities.

Candidate covariates (molecular aberrations) included the CNV of each chromosome and DNA methylations of selected genes with sufficient variation across the subjects. For each gene expression profile, we pre-selected a list of candidate covariates with sufficient pairwise association scores. Threshold values for incorporating associations into the model are reported in S6 Table.

We incrementally added the covariates to fit the expression data and employed a model selection procedure to balance goodness of fit and model complexity. Inclusion of covariates was prioritized with the following order: *cis*-acting CNV, *trans*-acting CNV, and DNA methylation.

After the logistic regression model of each gene expression profile was established, we grouped genes in terms of their covariates in the models. These groups of genes were the association modules. The dependent variables of gene expressions constituted the targets, while the independent variables of molecular aberrations constituted the effectors. Details about the module finding algorithm are described in our previous work [42, 43].

### Validation tests

#### Expression coherence of target genes

The expression coherence of target genes was evaluated using the correlations amongst target gene expressions within each association module. The distribution of correlation coefficients was compared to a background of correlations of 1000 randomly selected genes from the chosen dataset. A Kolmogorov-Smirnov (KS) test was applied to examine the difference in correlations between the module and background distributions. We applied both two-sided and one-sided KS tests to identify if a deviation exists, and if so, whether it is skewed in a common direction amongst East Asian female validation datasets. To determine the significance of a module for this test, an adjusted *p*-value was developed to account for differences in module size. Smaller modules would tend to have larger *p*-values, whereas larger modules are more likely to be identified as significant. To mitigate this bias, an adjusted *p*-value was calculated by selecting a random group of genes of the same dimension as the test set and comparing each to the background distribution. This process was repeated 1000 times for each module, with the original *p*-value ranked within this set. The final position of the *p*-value relative to the 1000 random samples would determine the significance (with threshold *p* < 0.05) of the module.

#### Prognostic power of association modules

We validated prognostic power of association modules with both Cox regression coefficients [62] and log-rank *p*-values of the Kaplan-Meier curves [63]. Cox regression coefficients gauged the dependency of a patients hazard function on observed variables. Negative associations with survival times exhibit positive Cox regression coefficients. For each module, we evaluated the distribution of Cox regression coefficients of its target gene expression profiles. The prognostic power of an association module was quantified by the KS statistic of its Cox regression coefficient distribution against a background distribution of coefficients for all genes in the selected dataset. Modules with an adjusted *p*-value < 0.05 were selected as significant.

In addition to the distribution of Cox regression coefficients, we also intended to demonstrate that an aggregate index derived from the data of an association module could quantify its prognostic power. To fulfill this goal we used the median over the expression profiles of target genes as the aggregate biomarker. In each dataset, the patients were divided into two groups in terms of whether their aggregate biomarker values exceeded the average expression values over all genes in the data. The log-rank *p*-value of the Kaplan-Meier curves of the two groups was used to measure prognostic power (* p* < 0.1).

### Validation of segment boundaries

Chromosomal arms were used as natural segmentation boundaries for the CNV data. To verify this decision, we applied the Circular Binary Segmentation algorithm [64] to identify segments of consistent CNV on the individual samples of the training data. We visually inspected the segment boundaries on individual samples to identify candidate boundary partitions across all samples. The results of the Circular Binary Segmentation are illustrated using heatmaps of the CNV data in S5 Fig.

For the majority of chromosomes we found that either no clear segment boundaries were identified, or the chromosomal arm partitions were sufficient. However, chromosomes 4, 7, 8 and 12 demonstrated evidence of additional boundaries. We therefore ran the module finding algorithm again on the new segment CNV data to identify whether using these additional boundaries would affect our final results in the study. None of the *cis*-acting CNV or *trans*-acting CNV modules for chromosomes 4, 8 and 12 passed all of the validation tests. In contrast, the chromosome 7 *cis*-acting CNV module was still found to be significant. The Kaplan-Meier curves for this module are presented in S6 Fig.

### False-discovery rates of pairwise associations and module discovery

A large number of pairwise models were generated between effector aberrations (*cis*-acting CNV, *trans*-acting CNV and methylations), and target mRNA gene expressions. It is likely that some of these significant associations have arisen by chance in the data. To assess the proportion of such discoveries, we calculated a false-discovery rate (FDR) for each effector aberration type [65, 66]. The data was permuted for each sample, and pairwise calculations were assessed. Significant associations were identified using the thresholds previously employed. The process was repeated 100 times for each molecular aberration type, with the number of significant associations recorded for each repetition. The expected number of false discoveries was calculated over the repetitions and the FDR evaluated as follows:

$$\frac{expected\;\#false\;positives\;according\;to\;the\;null\;model}{\#positive\;calls\;from\;the\;data\;}\;$$

[66]. We also assessed the significance of a large number of association modules using validation tests of passenger coherence and prognostic value. To assess the FDR of the validation tests, we first randomly assigned genes to modules of equal sizes as the original 44 reported in the study. The validation tests of passenger coherence and prognostic value were then applied to the randomly generated modules. The number of significant modules was recorded for each individual test, and also for how many pass all validation tests. This process was repeated over 200 runs and the expected value of false negatives calculated by averaging the significant modules over all repetitions.

FDR's of 20% and 46% were calculated for intra-CNV and inter-CNV pairwise associations respectively. A relatively high FDR of 75% reported for the methylation pairwise associations is likely attributed to the small sample size of the data and large number of features. The validation procedures at module level were designed to introduce larger external datasets into the analysis to verify the inferred modules of the Taiwan data. An overall FDR < 0.002 indicates that we can reduce the impact of false positives from pairwise models at the module level of the analysis. The complete results of the validation FDR rates for pairwise associations and validation tests are reported in S7 Table.

### Partial least square analysis of dependency between two groups of variables

Partial least squares (PLS) is a dimension reduction methodology, with certain similarities to principal component analysis (PCA). Like PCA, PLS seeks to obtain linear combinations of manifest covariates that are orthogonal to one another (i.e. components). A bilinear decomposition of the predictors X can be formed such that,

$$X=T_{m}P_{m}^{\text{T}}+E_{m}$$

where T_*M*_ = t_1_, t_2_, …, t_*M*_ are the *m* latent variables (or scores), P_*M*_ = p_1_, p_2_, …, p_*M*_ are the weights (or loadings) on the components and E is a residual error term. In contrast with the global variance maximizing goal that defines PCA components (X^T^X), PLS attempts to maximize X and response Y covariance (i.e. X^T^Y). For PLS, a linear combination has to be obtained for both the predictors and the response [67]. This is achieved by an iterative process to compute the optimal weight vectors to maximize covariance between X and Y. The components are ordered by their maximal covariance with the response. As such, removing weaker components from the model will often maintain explanatory power in a PLS regression.

We used PLS to analyze the dependency between the target genes of our inferred association modules. PLS allows us to gain an insight into how the modules behave as a complete set of genes, rather than limit the study to pairwise correlations only. With PLS we can summarize modules in a few latent components that maximize the covariance between two sets of target genes (considered as X and Y blocks). If we retain only the first two PLS components, we are able to plot the results on a two-dimensional ‘correlation circle’ (e.g. S3 Fig). Each point on the circle demonstrates the correlation between the gene expression and the PLS component axes. The cosine of the angle between the gene locations in the plot will indicate the correlation between module members projected onto these two component axes. In addition, the orthogonal projection of the module members onto the component axes will demonstrate the respective loading on each component. We can also indicate the variance explained by one block on the other by calculating a cumulative *R^2^* for each component retained in the model. This measure gives some indication of the dependency between target genes in the comparative modules.

### Pubmed co-citation and OMIM analysis of selected genes

To find candidate effector genes on inferred association modules, we examined whether some genes were more frequently co-cited with variations of the keyword lung cancer from prior studies. We wrote a Pearl script to query the NCBI PubMed database and counted the number of publications where the keyword and the name of a gene were co-present in the text. We then sorted all the genes according to their co-citation numbers and identified the intersection of the top-ranking (5%) genes and members in each module.

Whilst providing a useful indication of more established oncogenes in the literature, the co-citation approach is susceptible to false negatives that have been more recently discovered. To relieve this problem, we used the NCBI OMIM database of cancer-related genes to search for intersection with the target genes of the three significant modules.

### QIAGEN's Ingenuity pathway analysis

A pathway enrichment was performed on the target genes of all significant modules using QIAGEN's Ingenuity pathway analysis (IPA) (http://www.qiagen.com/ingenuity) [58]. The IPA software provides matches of functions or pathways with a significant overlap with the target genes. For each pathway, a *p*-value was calculated using a right tailed Fisher's exact test to assess the likelihood that any identified overlap of genes was statistically significant (i.e. not due to random chance). As the software performs tests on a large number of pathways, it is appropriate to adjust for multiple testing and provides an adjusted *p*-value using the Benjamini-Hochberg method [65]. Significance was determined by *p* < 0.01. The results were further filtered to report only pathways with an intersection > 4 genes.

## SUPPLEMENTARY MATERIAL FIGURES AND TABLES


