# Supplementary material for: Putative effectors for prognosis in lung adenocarcinoma are ethnic and gender specific
Source: Oncotarget. 2015 Jun 22;6(23):19483–99. doi: 10.18632/oncotarget.4287 (PMC4637300; doi:10.18632/oncotarget.4287)
Supplement: Supplementary file 1 [file oncotarget-06-19483-s001.pdf]

DATA SOURCES AND PRE-PROCESSING

Data sources

The training set of 32 Taiwanese female lung adenocarcinoma patients consists of the data of transcriptomes (Affymetrix U133plus2.0 microarrays), CNV (Affymetrix SNP6.0 microarrays), and DNA methylations (Illumina Infinium). In each patient the measurements from the tumor tissue and the adjacent normal tissue were provided.

Validation data were identified and downloaded from open-source locations (as detailed in Table 1). These included the Gene Expression Omnibus (GEO, http://www.ncbi.nlm.nih.gov/geo/), The Cancer Genome Atlas (TCGA, https://tcga-data.nci.nih.gov/tcga/) and caArray (https://array.nci.nih.gov/caarray/home.action). The four validation sets consist of transcriptomic and survival data. The Japanese dataset contains the transcriptomic data of Agilent G4112F microarrays on 117 patients. The Korean dataset contains the transcriptomic data of Affymetrix U133plus2.0 microarrays on 63 patients. The US_1 source is the TCGA lung adenocarcinoma (LUAD) dataset. We selected the transcriptomic data of Illumina RNAseq on 244 Caucasian patients. TCGA provides four levels of data with an increasing extent of processing and interpretation. We used level 2 data in our analysis as they contained probe-level information of microarray data. The US_2 data also includes patients from diverse ethnic backgrounds. We selected the transcriptomic data of Affymetrix U133A on 294 Caucasian patients. Probe information for the specific microarray platform was used to assign gene information (i.e. gene name, chromosome number, genomic location)

Data normalization

The Taiwanese training set consists of 3 types of data: copy number variations, DNA methylations and mRNA expressions. To incorporate the heterogeneous sources of data in the same integrated model, we had to convert them into the same format with compatible scales. We treated each feature as a discrete random variable. For such features with numerical values, simple quantization with hard thresholds results in substantial information loss. To avoid this problem we adopted a probabilistic quantization procedure to convert a measurement outcome into a probabilistic vector over the discrete states. This procedure hypothesizes that the underlying molecular states are discrete with uncertainty and/or different mixture coefficients in a population of cells, and the uncertainties or mixture coefficients are reflected in the magnitudes of measurement outcomes. Therefore, information pertaining to magnitudes of measurement outcomes are preserved in the probability vectors of the discrete states.

The data of mRNA and DNA methylations constitute continuous measurements from microarrays. We converted these continuous values into probability vectors of trinary states – up, down regulation or no change. For each dataset, denote z_{ij} the observed value of probe i on sample j, and x_{ij} its discrete hidden state. The following procedures convert each z_{ij} into a probability vector $(P(x_{ij}=-1), P(x_{ij}=0), P(x_{ij}=1))$.

1. If z_{ij} has a missing value, then assign equal probability $(P(x_{ij}=-1)=\frac{1}{3}, P(x_{ij}=0)=\frac{1}{3}, P(x_{ij}=1)=\frac{1}{3})$ to each state.

2. Rank-transform z_{ij} into the cumulative distribution function (CDF) value $y_{ij} \in [0,1]$. For the datasets reporting relative values (Agilent arrays), rank transformation is applied to the entire matrix. For the datasets reporting absolute values (Affymetrix arrays), each feature is rank-transformed separately. This is because we want to capture the relative variation of features across different samples instead of comparing the values of distinct features. DNA methylation data are scaled in $[0, 1]$ thus need not be rank-transformed.

3. Convert y_{ij} into a probability vector $(P(x_{ij}=-1), P(x_{ij}=0), P(x_{ij}=1))$ with a specific quantization function. Intuitively, a data point with a low CDF value is more likely to be down-regulated ($P(x_{ij}=-1)$ is high), and a data point with a high CDF value is more likely to be up-regulated ($P(x_{ij}=1)$ is high). This intuition is translated into the requirements that a quantization function is monotonic and maps $y_{ij}=0$ into $P(x_{ij}=-1)=1$ and $y_{ij}=1$ into $P(x_{ij}=1)=1$. We chose polynomial functions f_γ and $\bar{f}_\gamma$ as the quantization curves.

$$\begin{aligned} P(x_{ij}=1|y_{ij},\gamma) &= f\gamma(y_{ij}) \equiv y_{ij}^{\gamma} \\ P(x_{ij}=-1|y_{ij},\gamma) &= \bar{f}\gamma(y_{ij}) \equiv (1-y_{ij})^{\gamma} \\ P(x_{ij}=0|y_{ij},\gamma) &= 1-P(x_{ij}=1|y_{ij},\gamma)-P(x_{ij}=-1|y_{ij},\gamma) \end{aligned} \quad (1)$$

Parameter γ controls the "soft thresholds" of assigning x_{ij} to be $+1$ or -1. A higher γ lifts the threshold on y_{ij}(and $1-y_{ij}$) of calling the hidden state x_{ij} to be 1 (and -1). Thus a higher γ raises $P(x_{ij}=0)$ and reduces $P(x_{ij}=\pm 1)$.

4. Quantization results are sensitive to γ values. To reduce the bias induced by a specific quantization function we assigned weights (prior) on $f\gamma$ functions and integrate the transformed values over a family of quantization functions. In this work we chose an exponential prior $e^{-(\gamma-1)}$ and restricted $\gamma \in [1, \infty)$. The averaged quantization outputs are:

$$\begin{aligned} P(x_{ij}=1|y_{ij}) &= \int_1^{\infty} e^{-(\gamma-1)} f_\gamma(y_{ij})\, d\gamma = \frac{y_{ij}}{1-\log y_{ij}} \\ P(x_{ij}=-1|y_{ij}) &= \int_1^{\infty} e^{-(\gamma-1)} \bar{f}_\gamma(y_{ij})\, d\gamma = \frac{1-y_{ij}}{1-\log(1-y_{ij})} \\ P(x_{ij}=0|y_{ij}) &= 1-P(x_{ij}=1|y_{ij})-P(x_{ij}=-1|y_{ij}) \end{aligned} \quad (2)$$

The exponential prior $e^{-(\gamma-1)}$ was chosen for the following reasons. First, large γ values are penalized because they assign the probability mass to $x_{ij}=0$ for most y_{ij} values. An exponential prior naturally penalizes large γ values. Second, it ensures the existence of the integrals in equation 2. Third, the requirements that $P(x_{ij}=1|y_{ij}=1)=1$ and $P(x_{ij}=-1|y=0)=1$ are satisfied. Fourth, the most justified single value of γ is $\hat{\gamma}=\log 3/\log 2$ because it assigns an equal probability (1/3) for each state when the input CDF $y_{ij}=0.5$. The marginal quantization curves indeed resemble the quantization curves generated by $\hat{\gamma}$.

These procedures converted the measurement value of one probe in one sample into a probability vector. Genes are the elementary units in our analysis. The expression and DNA methylation of a gene are often measured by multiple probes. The probe-level data were first rank-transformed into CDF values. We generated gene-level data by merging the probe data corresponding to the same genes. The entry of each gene in each sample was the average over all probe values of the corresponding gene and sample.

To examine gender specific effects, we required the expression data of the 4 validation sets to be split by gender. The data were first rank-transformed into CDF values, and subsequently split into separate male and female subdata.

CNV data processing

The aforementioned procedures of data normalization need to be modified on CNV data as the underlying assumption mismatches the empirical characteristics of CNV measurements. Equation 2 gives the probability of each trinary state x given the CDF value of its measurement outcome y. For each specific probe (gene), y is uniformly distributed in $[0,1]$. Thus the total probability of encountering the up-regulation state $(x=1)$ is

$$\begin{aligned} P(x=1) &= \int_0^1 P(y)P(x=1|y)dy \\ &= \int_0^1 \frac{y}{1-\log y} dy \\ &\approx 0.3663 \end{aligned} \tag{3}$$

Similarly, $P(x=-1)\approx 0.3663$ and $P(x=0)\approx 0.2674$. This distribution stipulates that the fractions of amplification/deletion/no change entries in the CNV data are approximately comparable. In reality, the vast majority of the CNV data entries do not deviate from normal values (0s for two-channel microarrays). Only about 1% of the data points have log2 ratios ≥ 1 or ≤ -1. Therefore, the quantization function in equation 2 severely distorts the global characteristic of the CGH array data.

To reduce this distortion we introduced an extra parameter β to the quantization function:

$$\begin{aligned} P(x_{ij}=1|y_{ij}) &= \frac{y_{ij}^{\beta}}{1-\log y_{ij}^{\beta}} \\ P(x_{ij}=-1|y_{ij}) &= \frac{1-y_{ij}^{\beta}}{1-\log(1-y_{ij}^{\beta})} \\ P(x_{ij}=0|y_{ij}) &= 1-P(x_{ij}=1|y_{ij})-P(x_{ij}=-1|y_{ij}) \end{aligned} \tag{4}$$

The new $P(x_{ij}=1|y_{ij})$ and $P(x_{ij}=-1|y_{ij})$ shrink with increasing β values. We adjusted β to make the global distribution $(P(x=1), P(x=0), P(x=-1))$ obtained from equation 4 close to the empirical distribution. We counted the fractions of entries exceeding $log_2(3/2)=0.585$ (f_1) and below $log_2(1/2)=-1$ (f_2). For simplicity we set the global empirical probability of amplification and deletion to be equal:

$$P(x=1)=P(x=-1)\approx \frac{1}{2}(f_1+f_2) \tag{5}$$

We then found the parameter value $\hat{\beta}$ that fit the following equality:

$$\begin{aligned} &\int_0^1 \frac{x^{\hat{\beta}}}{1-logx^{\hat{\beta}}}dx \approx P(x=1) \\ &\int_0^1 \frac{(1-x)^{\hat{\beta}}}{1-log(1-x)^{\hat{\beta}}}dx \approx P(x=-1) \end{aligned} \tag{6}$$

The estimated parameter $\hat{\beta}$ was substituted in equation 4 in probabilistic quantization.

Unlike mRNA expressions or DNA methylations, the elementary subunits of CNV data are segments bounded by amplification and deletion events instead of genes. In an earlier version of the module-finding algorithm [1], we partitioned each chromosome into smaller segments according to their CNV data. In this particular dataset, however, we observed that CNV data were mostly coherent within each chromosomal arm. To simplify computation, a decision was therefore made to use chromosomal arms as natural segmentation boundaries. We aggregated the measurements of probes on each chromosomal arm and used the median over the corresponding probe values as the proxy CNV value of a chromosome segment.

Assessment of purity and ploidy of the tumors

We employed the ABSOLUTE algorithm to estimate both ploidy and purity of samples in the training set. The inferred results are reported in S5 Table. The inferred purity indicates most samples retain a significant fraction of cancer DNA. Therefore, the association modules reflect information in cancer genomes. Inferred ploidy indicates many cancer samples undergo copy number amplifications in most spots in the genomes. The fluctuations of CNVs provide a necessary condition to build associations between CNV and mRNA data.

Clustering DNA methylation, and mRNA expression data

Correlated effector aberrations tend to fit the same set of target genes. It is therefore more efficient to cluster the effector aberrations and build association modules with the proxy data derived from clustered effector aberrations. We propose a graph-based method to cluster DNA methylation and mRNA expression data. In brief, by setting a threshold on correlation coefficients we could build a graph with genes as nodes and edges connecting correlated genes. By gradually lowering the threshold we extracted cliques – maximally and completely connected components – with increasing sizes. Highly connected cliques were then merged to form clusters.

1. Calculate the correlation coefficient matrix of the data.
2. Sort the pairwise correlation coefficients with a decreasing order.
3. Set the threshold to the highest correlation coefficient value.
4. Build a graph G with nodes as genes and edges connecting genes whose correlation coefficients exceed the threshold.
5. Find cliques on G.
6. Repeat the following steps until the threshold value ≤ 0.4.
    - (a) Lower the threshold value. Add edges to G according to the new threshold value.
    - (b) Find all existing clique pairs that become fully connected on the updated G. Among them merge the clique pair with the largest joint size.
    - (c) Find the nodes that are connected to existing cliques with the new threshold value. Assign each node to the largest connecting clique.
    - (d) Find newly emerged cliques with the new threshold value by incrementally adding nodes with high connectivity.
    - (e) Update clique information.
7. Start with cliques as clusters, repeat the following steps until no clusters are mergeable:
    - (a) For each pair of clusters, calculate the means of intra-cluster and inter-cluster correlation coefficients. Also calculate the mean of the joint cluster correlation coefficient.
    - (b) Find the cluster pairs whose joint mean correlation coefficient ≥ 0.4 and the difference between the intra-cluster mean correlation coefficient and inter-cluster mean correlation coefficient ≤ 0.1.
    - (c) Among the candidate cluster pairs merge the ones with the largest joint size.
    - (d) Update the cluster information.

CONSTRUCTION OF ASSOCIATION MODULES

Pairwise associations

For each pair of molecular aberration (segment CNV, DNA methylation) and mRNA gene expression, we evaluated their Pearson correlation coefficient, log-likelihood ratio of the logistic regression model, and *p*-value obtained from both χ^2 approximation of log-likelihood ratio and permutation tests. Denote x a candidate effector and y a target gene expression. We express the conditional probability $P(y|x)$ with a logistic regression model:

$$P(y|\mathrm{x}) = \frac{1}{Z(x)} e^{\lambda f(x) y}, \lambda \geq 0 \qquad (7)$$

where either $f(x)=x$ (the effector activates the target) or $f(x)=-x$ (the effector represses the target). λ is a nonnegative parameter. $Z(x)=1+e\lambda+e^{-\lambda}$ is the partition function that normalizes the conditional probabilities.

Denote $D \equiv (x^1, y^1), \cdot\cdot\cdot, (x^m, y^m)$ the observed instantiations of x and y over m samples. The log-likelihood function of the observed data D is

$$\begin{aligned} L(D;\lambda) &= \sum_{k=1}^{m} \left\{ log(P(x^k)) + \log(\frac{1}{Z(x)}) + \lambda f(x^k) y^k \right\} \\ &= \sum_{C_x, C_y} \left\{ \mathrm{N}(\mathrm{C}_x) \log(\mathrm{P}(\mathrm{C}_x)) + \mathrm{N}(\mathrm{C}_x, \mathrm{C}_y) \right. \\ &\quad \left. [-log(Z(C_x)) + \lambda f(C_x) C_y] \right\} \end{aligned} \qquad (8)$$

where C_x and C_y stand for configurations of x and y, and $N(C_x), N(C_x, C_y)$ the fractional counts for configurations C_x and (C_x, C_y) over all samples. Using probabilistic quantization each entry x_{ij} was converted into $(P(x_{ij}=-1), P(x_{ij}=0), P(x_{ij}=1))$, where i and j are gene and sample indices. The fractional count for a state configuration (C_x, C_y) is

$$N(C_x, C_y) = \sum_j P(y_j = C_y) P(x_j = C_x) \qquad (9)$$

The maximum likelihood parameter $\hat{\lambda}$ was numerically estimated using the Newton-Raphson method. Denote $F(\lambda) \equiv L'(D;\lambda)$ and $G(\lambda) \equiv F'(\lambda)$. Set $\lambda^0 = 1$ as the initial value of λ. Iteratively execute the following updates until either λ^t converges or $\lambda^t \leq 0$:

$$\lambda^{t+1} = \lambda^t - G^{-1}(\lambda^t) F(\lambda^t) \qquad (10)$$

To test the significance of the pairwise association, we introduced a null model M_0 where x and y are independent $(\lambda = 0)$ and treated equation 7 as an alternative model M_1. The log-likelihood $L(D; M_1)$ was given by equation 8. The log-likelihood $L(D; M_0)$ was simply $-m \; log \; Nx$, where Nx denotes the number of states of variable x.

Given the observed data D and two nested models M_0, $M_1 \supseteq M_0$, we incurred a standard hypothesis testing procedure to calculate the log-likelihood ratio and χ^2 p-value:

$$L(D; M_0, M_1) = L(D|M_1) - L(D|M_0)$$
$$p = 1 - \chi_1^2(2L(D; M_0, M_1)) \quad (11)$$

where χ_1^2 is the χ^2 CDF function with one degree of freedom.

The χ^2 p-values tend to over-estimate the significance of the testing results. Thus we also evaluated the p-values of permutation tests and reported the supremum of χ^2 and permutation p-values. Permutation p-values were calculated by the following procedures:

1. Quantize the aberration and expression CDF values into binary or trinary states. For trinary variables we choose 0.4 and 0.6 as thresholds.
2. Count the number of samples where the aberration and expression states are consistent with the truth tables from M_1. Denote this number as n_c.
3. Find the additional feature variables X_v that appear in M_1 but not in M_0.
4. Repeat the following steps 10,000 times:
   (a) Randomly permute the data in X_v.
   (b) Count the number n_p of samples where the permuted aberration and expression states are consistent with M_1.
5. The p-value is the fraction of n_p's exceeding n_c.

The reported p-value for each pairwise association is the maximum of the p-values derived from χ^2 approximation and permutations.

Building association models for individual genes

Not all types of molecular aberrations are equally likely to drive gene expressions. Some candidate effectors provide direct explanations for gene expressions without requiring many mechanistic assumptions underlying gene regulation (e.g., *cis*-acting effects with CNVs). Others have a greater number of features thus are likely to introduce spurious associations. We proposed a *layered modeling framework* to prioritize molecular aberrations differently and incrementally incorporating candidate effectors to the model according to their priorities. Molecular aberrations are categorized with the following priorities.

1. **Level 1**: segment CNVs on the same chromosomal arm as the target (CNV *cis*-acting effects) and DNA methylations on the target.
2. **Level 2**: positive associations with segment CNVs on distinct chromosomes from the target (CNV *trans*-acting effects) and negative associations with DNA methylations of non-target genes.
3. **Level 3**: negative associations with CNV *trans*-acting effects when the target is not explained by level 1–2 models.

In addition, we placed an extra requirement for CNV *trans*-acting effects. To build an association between a segment CNV and a target gene on another chromosome, the segment must accommodate at least one intermediate regulator that likely modulates the expression of the target. The regulator gene expressions are positively associated with the segment CNVs, and the functional direction of the association between the regulator and the target gene expressions coincides with that of the association between the segment CNV and the target gene expression. 727 candidate regulators were pulled from three sources: human transcription factors from TRANSFAC [2], human transcription factors and signaling proteins from FanTom [3], and genes pertaining to cancer from the OMIM database [4].

Pairwise associations were filtered with different thresholds of log-likelihood ratios and correlation coefficients according to the levels and types of effectors. The thresholds applied in the analysis are reported in S6 Table.

We again applied logistic regressions to build association models with multiple effectors. The procedures of calculating the maximum log-likelihood of a model are analogous to those for pairwise associations (equations 7–10).

The log-likelihood ratio, χ^2 p-value and permutation p-value between two nested models M_0, $M_1 \supseteq M_0$ were calculated analogous to equation 11. Here the degree of freedom d of the χ^2 function χ_d^2 is the number of additional features in M_1 compared to M_0.

The hypothesis testing procedures were applied in a model selection algorithm as follows. Suppose M_0 is a null model containing no effectors, M_1 is an association model at the current level. Consider adding a candidate effector x to the current association model M_1. We define the following model selection procedures as selection $(M_1, x, \theta, \theta')$:

1. Build a model M_2 containing a single effector x, and a model $M_{12} \supset M_1$ by adding an additional term of x to the logit of M_1.
2. Incur three hypothesis tests: M_2 against M_0, M_{12} against M_1, and M_{12} against M_2. Denote their p-values as p_0, p_1, p_2 respectively. The following operations are executed depending on the testing outcomes:
   (a) If $p_0 > \theta$, then discard x since its association with the target is weak.
   (b) If both $p_1 \leq \theta'$ and $p_2 \leq \theta'$, then the joint model M_{12} provides an additional explanatory power relative to both M_1 and M_2. Replace M_1 with M_{12}.
   (c) If $p_1 \leq \theta'$ and $p_2 > \theta'$, then the joint model M_{12} is better than M_1 but not M_2. This implies that the explanatory power of M_1 is dominated by M_2 but not the other way around. Replace M_1 with M_2.
   (d) If $p_2 \leq \theta'$and $p_1 > \theta'$, then the joint model M_1 is better than M_2 but not M_1. This implies that the

explanatory power of M_2 is dominated by M_1 but not the other way around. Discard x.

(e) If both $p_1 > \theta'$ and $p_2 > \theta'$, then neither M_1 nor M_2 can dominate the other, and the joint model does not provide an additional explanatory power. Keep M_1 and add M_2 to the current model list, yet do not merge M_1 and M_2.

θ and θ' stand for the p-value thresholds for testing a single-effector model (M_2) against an independent model (M_0) and testing a multi-effector model (M_{12}) against a null model by removing one effector (M_1 or M_2). The test is significant if the p-value is not greater than the threshold.

To build association models to explain a gene expression data y, we employed the layered modeling framework to incrementally add effector molecular aberrations to the existing models with the following procedures. Initially set the association model M as empty.

1. At level 1, implement the following steps.
   (a) Filter out candidate effectors according to the thresholds of log-likelihood ratios and correlation coefficients.
   (b) Among all the CNV segments located on the same chromosome of y, keep the one with the highest log-likelihood ratio.
   (c) For each remaining candidate effector x at level 1, apply selection (M, x, θ, θ') to each current model $M \in M$. The effectors are incorporated according to the following order: CNV *cis*-acting effects, DNA methylations.
2. At level 2, implement the following steps.
   (a) Filter out candidate effectors according to the thresholds of log-likelihood ratios and correlation coefficients.
   (b) Remove the CNV positive *trans*-acting associations that do not contain candidate regulators with consistent associations with both segment CNVs and target gene expressions.
   (c) If multiple CNV segments on the same chromosome have *trans*-acting effects with y, then only keep the one with the strongest log-likelihood ratio.
   (d) For each remaining candidate effector x at level 2, apply selection (M, x, θ, θ') to each current model $M \in M$. The effectors are incorporated according to the following order: CNV *trans*-acting effects, DNA methylations.
   (e) Multiple effector candidates of DNA methylations may possess significant explanatory power for they share correlated methylation profiles. To prevent including all these correlated DNA methylation effectors in the model selection process, we solicited one representative from each cluster of DNA methylation data. The representative DNA methylation profile possesses the highest log-likelihood ratio among the members in the same cluster. When incorporating higher-layered associations, only the representative DNA methylations will be included in the association model of the current level. Non-representative DNA methylations that pass the model selection procedures will be reported as effectors but not used in evaluating log-likelihood ratios. Notice model selection still operates at the DNA methylation profiles of single genes, and non-representative DNA methylations are still included as effectors. Clustering results are used only to reduce model complexity in model selection.
3. At level 3, implement the following steps.
   (a) Filter out candidate effectors according to the thresholds of log-likelihood ratios and correlation coefficients.
   (b) Remove the CNV negative *trans*-acting associations that do not contain candidate regulators with consistent associations with both segment CNVs and target gene expressions.
   (c) If multiple CNV segments on the same chromosome have *trans*-acting effects with y, then only keep the one with the strongest log-likelihood ratio.
   (d) For each remaining candidate effector x at level 3, apply selection(M, x, θ, θ') to each current model $M \in M$.

The outcome is a list of association models that explain the mRNA expression of each gene.

Assembling association models of individual genes to modules

Molecular aberrations on effector genes or genomic components often mis-regulate many downstream targets. We define an association module as a tuple consisting of three components: (1) observed effector molecular aberrations, (2) target genes, (3) regulators that mediate the effects between effectors and targets. From the association models of individual genes we incurred the following procedures to construct modules.

1. Group gene expressions by each candidate effector molecular aberration. Assign a gene to the targets of a effector molecular aberration if the latter appears in the association model(s) of the former.
2. Merge all the CNV *cis*-acting modules with segments on the same chromosome together. The effectors and targets are the union of those in all the member modules. Hence there is at most one CNV *cis*-acting module per chromosome.
3. Merge all the CNV *trans*-acting modules of identical functional direction and with segments on the same chromosome together. Merge intermediate regulators in addition to targets and targets. There are at most two CNV *trans*-acting modules per chromosome (one positive and one negative association).

4. Discard small modules with less than 20 target genes.
5. Construct an undirected graph GM of *mergeability* among the remaining modules according to the following criteria.
   (a) Do not merge modules with CNV *cis*-acting effects.
   (b) Do not merge module pairs with CNV *trans*-acting effects.
   (c) If the intersection of targets between two modules exceeds one third of the target set of the smaller module, then the two modules are mergeable and add an edge connecting them in GM.
   (d) If the intersection of targets between two modules exceeds 50 genes, then add an edge connecting the two modules in GM.
6. Find connected components in GM. Merge the modules in each connected component together.

VALIDATION OF ASSOCIATION MODULES

FDR evaluation of pairwise associations

False discovery rates (FDRs) quantify the expected fraction of false positives among the positive calls from a multiple hypothesis testing problem [5]. To simplify the testing procedures, we considered a positive call as a significant pairwise association between a effector and a target. The thresholds of log-likelihood ratios and correlation coefficients for determining positive calls are reported in S6 Table. Significant pairwise associations arising from the permuted data are false positives according to the null model. Lacking specific information about the distribution of noise, we created a simple null model by randomly permuting the effector aberration and target expression 100 times. Such permutation tests preserve the marginal distributions of individual components thus are widely used in evaluating the significance of detected signals. The empirical distribution of the number of significant pairwise associations arising from permuted data provides a reasonable measure for false positive numbers. A standard formula of FDR is

E {# false positives according to the null model}/# positive calls from the data

[6] The expected number of false positives can be directly calculated using its distribution from the permuted data. FDR rates are reported in S7 Table.

Reproducibility verification of associations in external datasets

We verified the reproducibility of the association modules by checking whether the associations extracted from each module are robust in East Asian and Caucasian external datasets. For each module, we incurred the following tests to verify the reproducibility of associations.

We computed the distributions of correlation coefficients between the target gene expressions in all external datasets. To quantify the strength of coherence, we compared the distribution of correlation coefficients with the background distribution derived from a reference set of genes. A reference set was generated by calculating correlation coefficients of 1,000 randomly selected pairs of genes in the dataset. We evaluated the *p*-value of the one-sided, two-sample Kolmogorov-Smirnov test between the two distributions of correlation coefficients.

Modules containing a large number of target genes are more likely to be found significant using the Kolmogorov-Smirnov test. To account for the effects of module size we generated 1,000 modules of randomly selected genes of the same size as the inferred modules and compared them to the previously generated background distributions. The *p*-value of the inferred module is ranked amongst the *p*-values of the randomly generated modules. The rank over the number of randomly generated modules provides an adjusted *p*-value.

Assessment of the prognostic power of association modules

We selected datasets for survival analysis according to the following criteria: (1) patient survival information was available, and (2) exact survival durations (in terms of days or weeks) instead of broad ranges were labeled. The Taiwan training data were excluded from the module selection due to a shortage of death events. The four external datasets all met these criteria. The prognostic power of biomarkers is typically measured by Cox regression coefficients. In survival analysis, Cox regression coefficients quantify the association of a set of independent variables (e.g., biomarker gene expression levels) with the hazard function of the population.

In the first stage, we treated the mRNA expression of each gene as an independent variable in the Cox regression model and evaluated its regression coefficient. To assess the prognostic power of an association module, we evaluated the distribution of Cox regression coefficients of its targets and compared this distribution with the background distribution of all genes. Both one-sided and two-sided, two-sample Kolmogorov-Smirnov tests were applied to calculate the *p*-values. Deviations from the background distribution in both directions provide useful prognostic information. Strong positive coefficients indicate negative associations with survival durations (higher expression levels are associated with shorter survival durations), and strong negative coefficients indicate positive associations with survival durations (higher expression levels are associated with longer survival durations).

Adjusted *p*-values were calculated once again to account for the effects of module size on the significance of the test statistic. We generated 1,000 modules of randomly selected genes of the same size as the inferred modules. The Cox regression coefficients were calculated for each of the genes in the generated modules and compared to the background distribution previously generated. The *p*-values of the Kolmogorov-Smirnov statistic for the inferred modules are ranked amongst the *p*-values from the randomly generated modules. The rank over the number of randomly generated modules provides an adjusted *p*-value accounting for module size.

In the second stage, we intended to demonstrate that an aggregate index derived from the data of an association module could quantify its prognostic power. We used the median over the expression profiles of target genes as the aggregate biomarker. Patients were divided into *low* and *high* expression groups according to whether their aggregate biomarker exceeded the average expression over all the genes in the data. The log-rank *p*-value was used to measure prognostic power.

Validation of segmentation boundaries

To validate the decision to chromosomal arms as segment partitions we employed the Circular Binary Segmentation (CBS) algorithm [7] to partition the patient data of each chromosome into segments with coherent CNV profiles. We visually inspected the segment boundaries on individual samples to identify candidate boundary partitions across all samples. Any full CNV segments generated from the CBS algorithm that differed from the chromosomal arm segmentation were entered into the module-building algorithm separately and verified to ensure that the results were consistent regardless of segmentation approach.

FDR evaluation of validation tests

As we are testing a large number of modules in the passenger coherence and prognostic power validation tests, we must consider the chance generation of false positive results. For a module to be deemed significant it must pass the passenger coherence and the two stages of the prognostic power tests. We can consider these tests individually and as a collective. To calculate an FDR statistic we randomly assign genes to modules the same sizes as the inferred modules. This process is repeated 200 times and each of the validation tests applied at each repetition. The number of significant modules are counted for each individual test and also for any randomly generated modules that pass both the passenger coherence and prognostic power tests.

Partial least squares to assess the dependency between association modules

It is possible that association modules identify different elements of a larger biological mechanism and therefore they may be interrelated. To investigate possible dependencies between association modules we employ a partial least squares (PLS) analysis [8]. Similar to principal components analysis (PCA), PLS is a dimension reduction method that allows the user to project high dimensional data onto a lower dimensional space, often capturing a high proportion of variance with only a few orthogonal latent variables. Unlike PCA, PLS incorporates information of the response, building latent variables that maximize the covariance between the predictors $\boldsymbol{X}$ and the response $\boldsymbol{Y}$. A bilinear decomposition of $\boldsymbol{X}$ can be formed such that

$$\boldsymbol{X} = \boldsymbol{T}_m \boldsymbol{P}_m^T + \boldsymbol{E}_m \quad (12)$$

where $\boldsymbol{T}_M = \boldsymbol{t}_1, \boldsymbol{t}_2, \ldots, \boldsymbol{t}_M$ are the m latent variables (or scores), $\boldsymbol{P}_M = \boldsymbol{p}_1, \boldsymbol{p}_2, \ldots, \boldsymbol{p}_M$ are the weights (or loadings) on the components and $\boldsymbol{E}$ is a residual error term. PLS is particularly beneficial for the current study as we can plot the target genes in each module as points in an object space representation. This allows the current analysis to be extended by considering the modules as a complete set of genes, rather than being limited to just pairwise associations between module members. By considering two modules as $\boldsymbol{X}$ and $\boldsymbol{Y}$ *blocks* of genes, PLS can build latent variables that maximize covariance (i.e. $\boldsymbol{X}^T\boldsymbol{Y}$) between the two sets of target genes.

We can visualize the associations of the module members by projecting them onto a two dimensional space, labeled a 'correlation circle'. The cosine of the angle between the gene locations in the plot indicates the association between the gene expressions and the distance from the center represents the proportion of variance explained by the first two PLS components. A cumulative R^2 can also be calculated to provide a quantitative measure of the dependency between a pair of association modules.

Co-citation analysis on PubMed and OMIM database

We incurred a search on the PubMed database to find targets from all modules that were co-cited in publications with variations of the key term "lung cancer". The citation counts for each target gene were then ranked, with the top-ranking 5% of genes selected as significant.

Co-citation count is susceptible to false negative driver genes that have been more recently discovered. To relieve this problem we consider the overlap of target genes in significant modules with the NCBI OMIM database [4] of cancer related genes. Whilst this database is not designed specifically for lung adenocarcinoma,

overlapped genes can be flagged as potential module effectors.

QIAGEN's Ingenuity pathway analysis

A pathway enrichment can be performed on the target genes of inferred modules in the study. This step is intended primarily to assess *trans*-acting CNV and methylation modules, as targets are not restricted to a single chromosome. We employed QIAGEN's Ingenuity pathway analysis (IPA) [9] to search for pathways amongst target genes. The software uses a right tailed Fisher's exact test to assess whether any identified overlap of genes between module targets and pathways is statistically significant (not generated by chance). As a large number of pathways are present and tested for in the IPA software, an adjusted *p*-value is calculated using the Benjamini-Hochberg method.

REFERENCES

1. Sintupisut N, Liu PL, Yeang CH. An integrative characterization of recurrent molecular aberrations in glioblastoma genomes. Nucleic Acids Research. 2013; 41:8803–8821.
2. Matys V, Fricke E, Geffers R, Gößling E, Haubrock M, Hehl R, Hornischer K, Karas D, Kel A, Kel-Margoulis OV, Kloos DU, Land S, Lewicki-Potapov B, et al. TRANSFAC®: transcriptional regulation, from patterns to profiles. Nucleic acids research. 2003; 31:374–378.
3. Kawai J, Shinagawa A, Shibata K, Yoshino M, Itoh M, Ishii Y, Arakawa T, Hara A, Fukunishi Y, Konno H, Adachi J, Fukuda S, Aizawa K, et al. Functional annotation of a full-length mouse cDNA collection. Nature. 2001; 409:685–690.
4. Hamosh A, Scott AF, Amberger JS, Bocchini CA, McKusick VA. Online Mendelian Inheritance in Man (OMIM), a knowledgebase of human genes and genetic disorders. Nucleic acids research. 2005; 33:D514–D517.
5. Benjamini Y, Hochberg Y. Controlling the false discovery rate: a practical and powerful approach to multiple testing. Journal of the Royal Statistical Society. Series B (Methodological). 1995; 57:289–300.
6. Storey JD, Tibshirani R. Statistical significance for genomewide studies. Proceedings of the National Academy of Sciences. 2003; 100:9440–9445.
7. Olshen AB, Venkatraman ES. Circular binary segmentation for the analysis of array-based DNA copy number data. Biostatistics. 2004; 5:557–572.
8. Wold H. Partial least squares. Encyclopedia of statistical sciences. 1985.
9. IPA, QIAGEN Redwood City, www.qiagen.com/ingenuity.

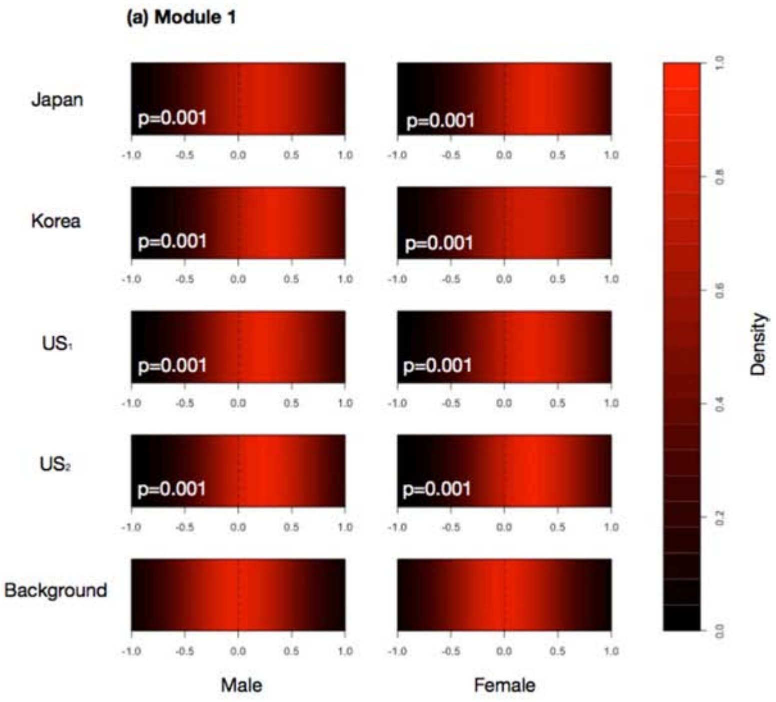
(a) Module 1
Japan
Korea
US1
US2
Background
p=0.001
p=0.001
p=0.001
p=0.001
p=0.001
p=0.001
p=0.001
p=0.001
Male
Female
Density

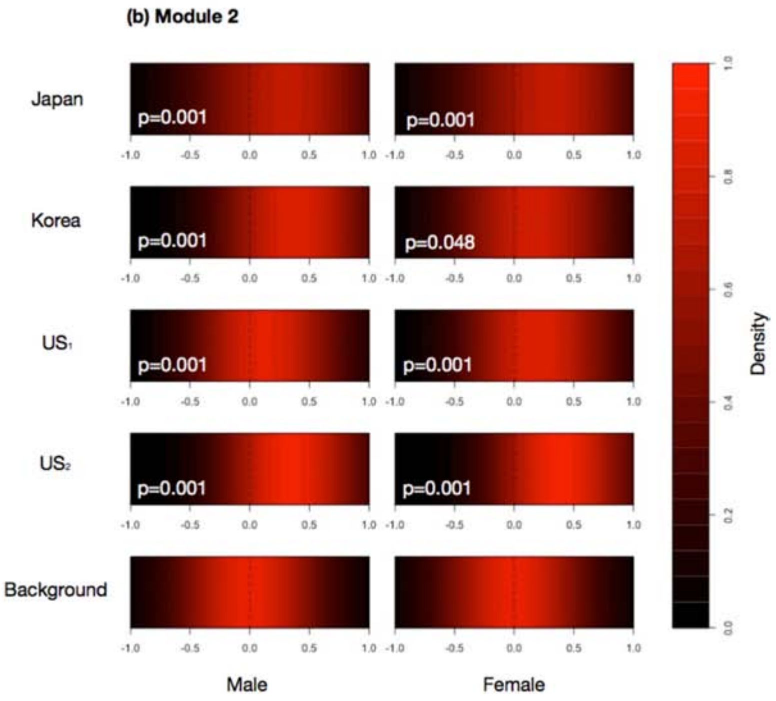
(b) Module 2
Japan
Korea
US1
US2
Background
p=0.001
p=0.001
p=0.001
p=0.048
p=0.001
p=0.001
p=0.001
p=0.001
Male
Female
Density

(*Continued*)

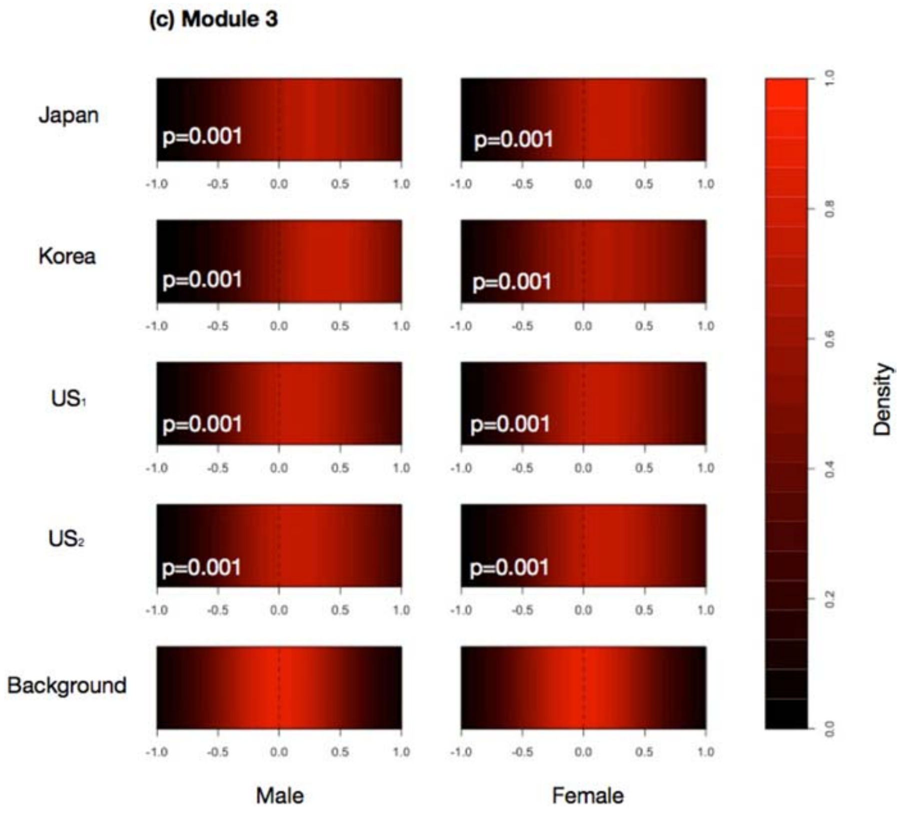

Supplementary Figure S1: Gender and ethnic specific coherence of target genes. Heatmaps illustrating the distributions of correlation coefficients among target gene expressions of both male and female external datasets. A distribution of 1000 randomly selected pairs of genes is included to provide a background comparison to compute the Kolmogorov-Smirnov statistic.

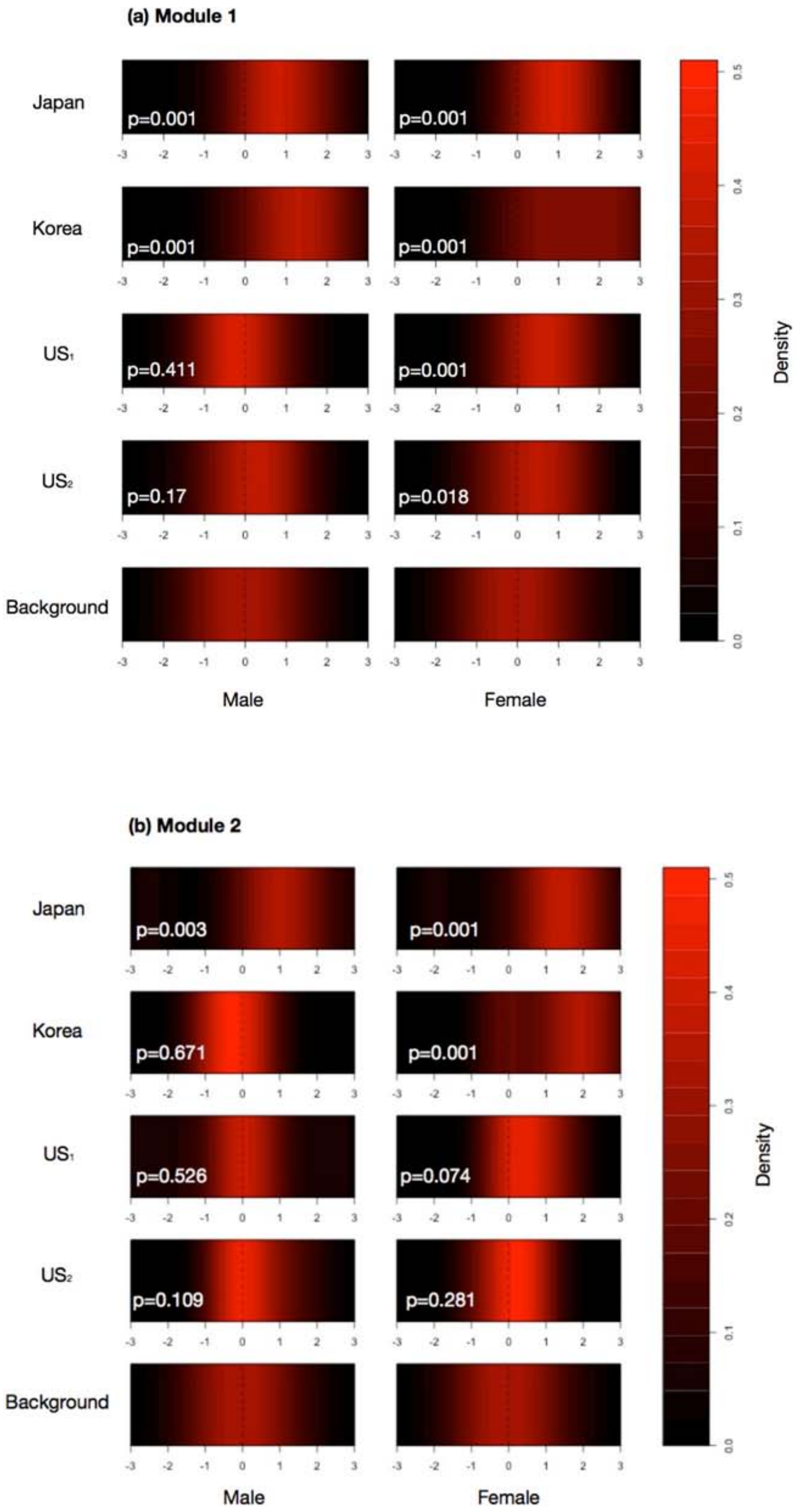
(a) Module 1
Japan
p=0.001
p=0.001
Korea
p=0.001
p=0.001
US1
p=0.411
p=0.001
US2
p=0.17
p=0.018
Background
-3 -2 -1 0 1 2 3
Male
Female
Density
0.0 0.1 0.2 0.3 0.4 0.5
(b) Module 2
Japan
p=0.003
p=0.001
Korea
p=0.671
p=0.001
US1
p=0.526
p=0.074
US2
p=0.109
p=0.281
Background
-3 -2 -1 0 1 2 3
Male
Female
Density
0.0 0.1 0.2 0.3 0.4 0.5

(Continued)

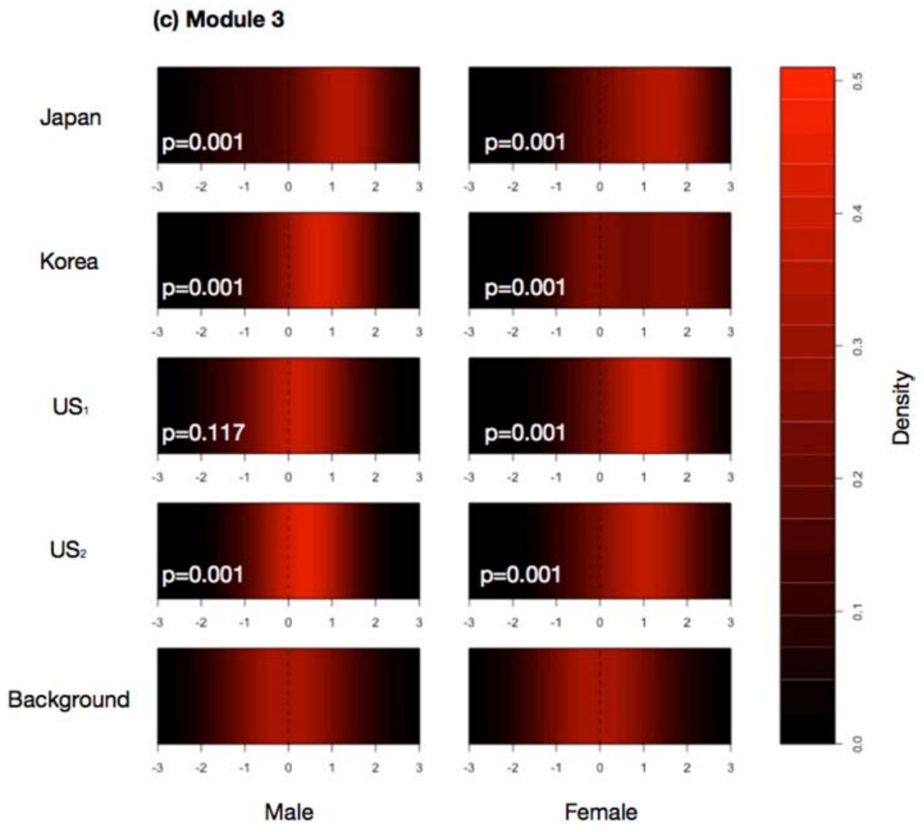

Supplementary Figure S2: Gender and ethnic specific prognostic values of association modules. Heatmaps illustrating the distributions of Cox regression coefficients among target gene expressions of both male and female external datasets. The Cox coefficients of all genes in the external dataset are included to provide a background comparison to compute the Kolmogorov-Smirnov statistic.

(a) PLS Circle of Correlations for Modules 1 (red) and 2 (blue)

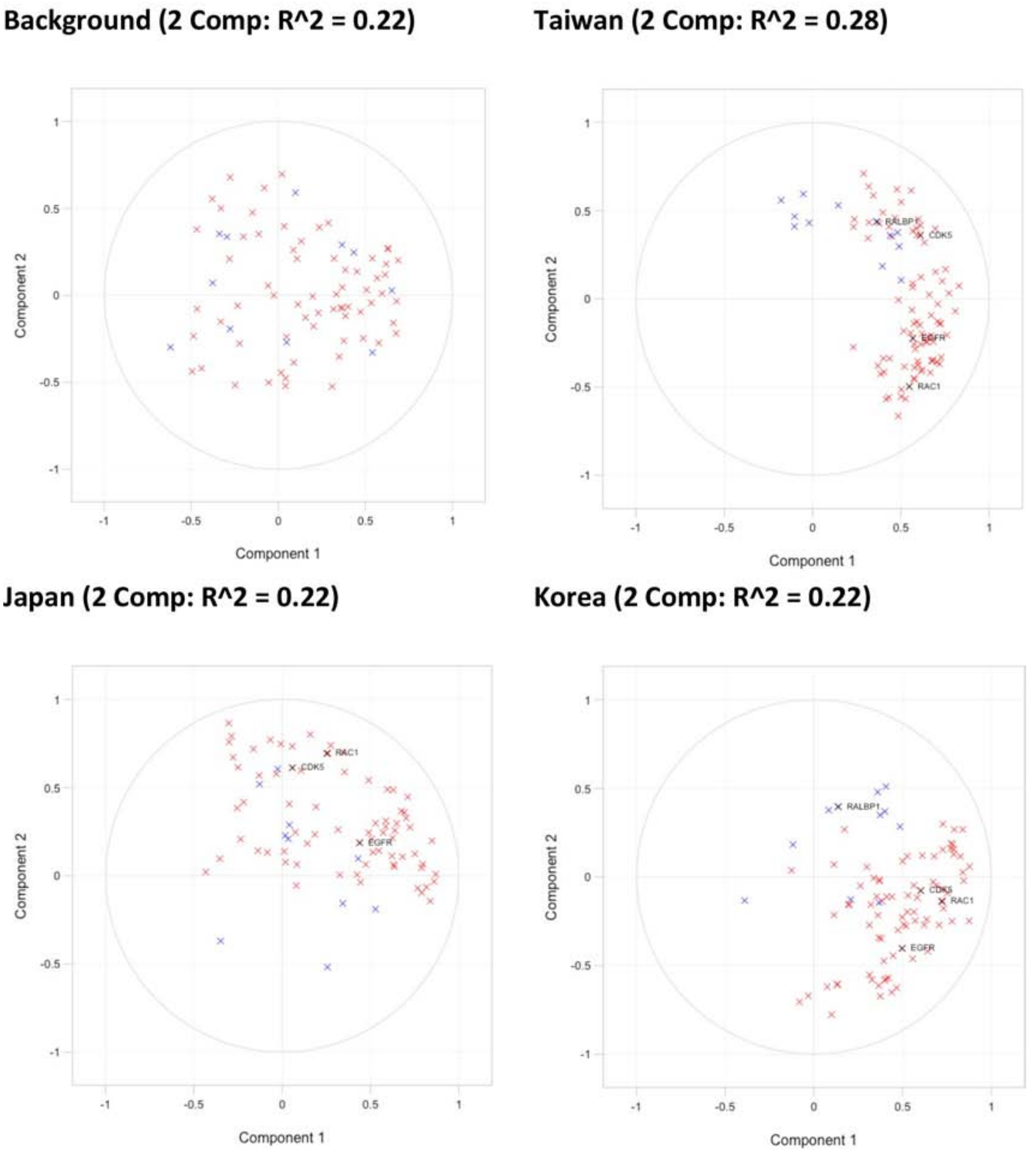

(*Continued*)

(b) PLS Circle of Correlations for Modules 1 (red) and 3 (blue)

Background (2 Comp: R^2 = 0.19)

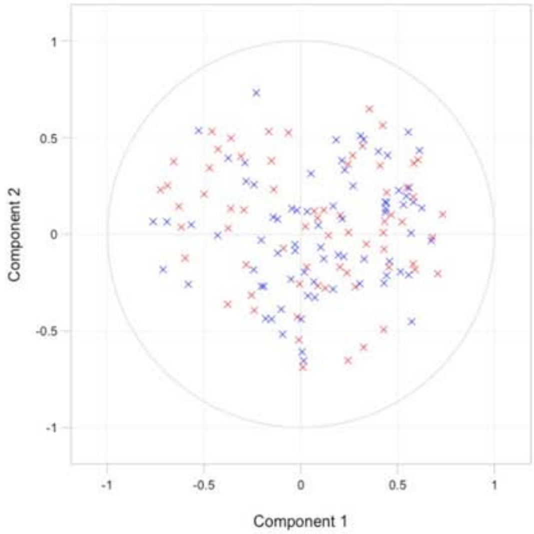

Taiwan (2 Comp: R^2 = 0.38)

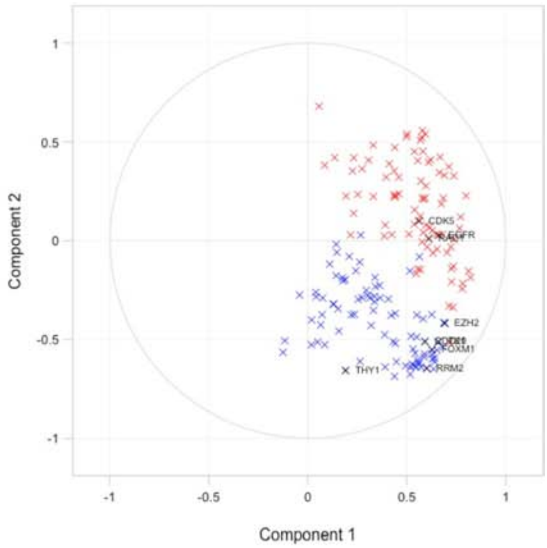

Japan (2 Comp: R^2 = 0.32)

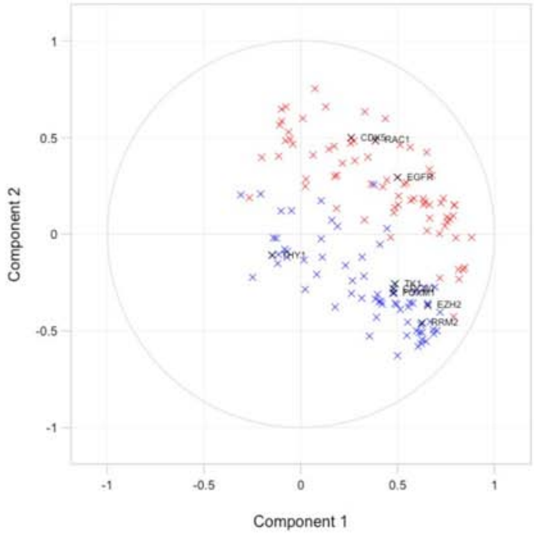

Korea (2 Comp: R^2 = 0.42)

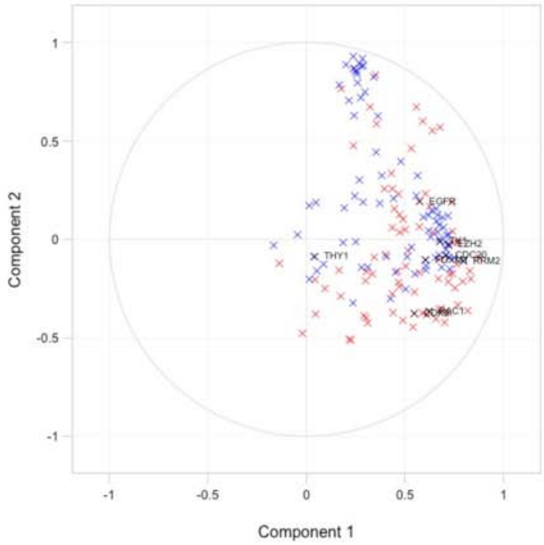

(*Continued*)

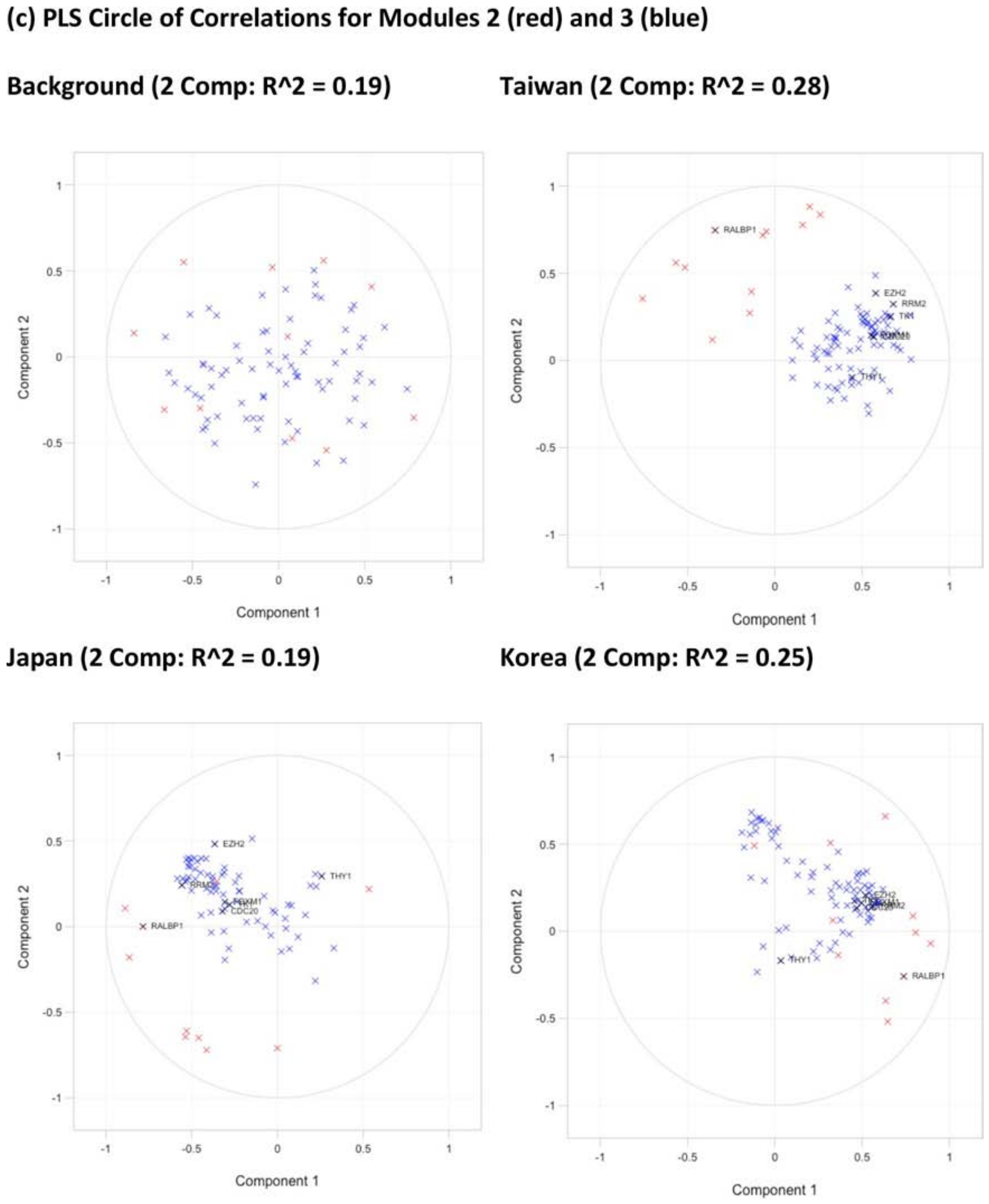

Supplementary Figure S3: Partial least squares to assess the association between modules. Correlation circles are used to display the target genes projected onto the first two PLS components for two module sets of target genes. For each module comparison we include the plots of a random background collection of genes selected to reflect the same dimensions as the test modules (top left), and also comparisons in the Taiwanese (top right), Japanese (bottom left) and Korean (bottom right) datasets. We include an R^2 statistic based on the 2 PLS components to give some indication of the association between modules sets in each East Asian female dataset.

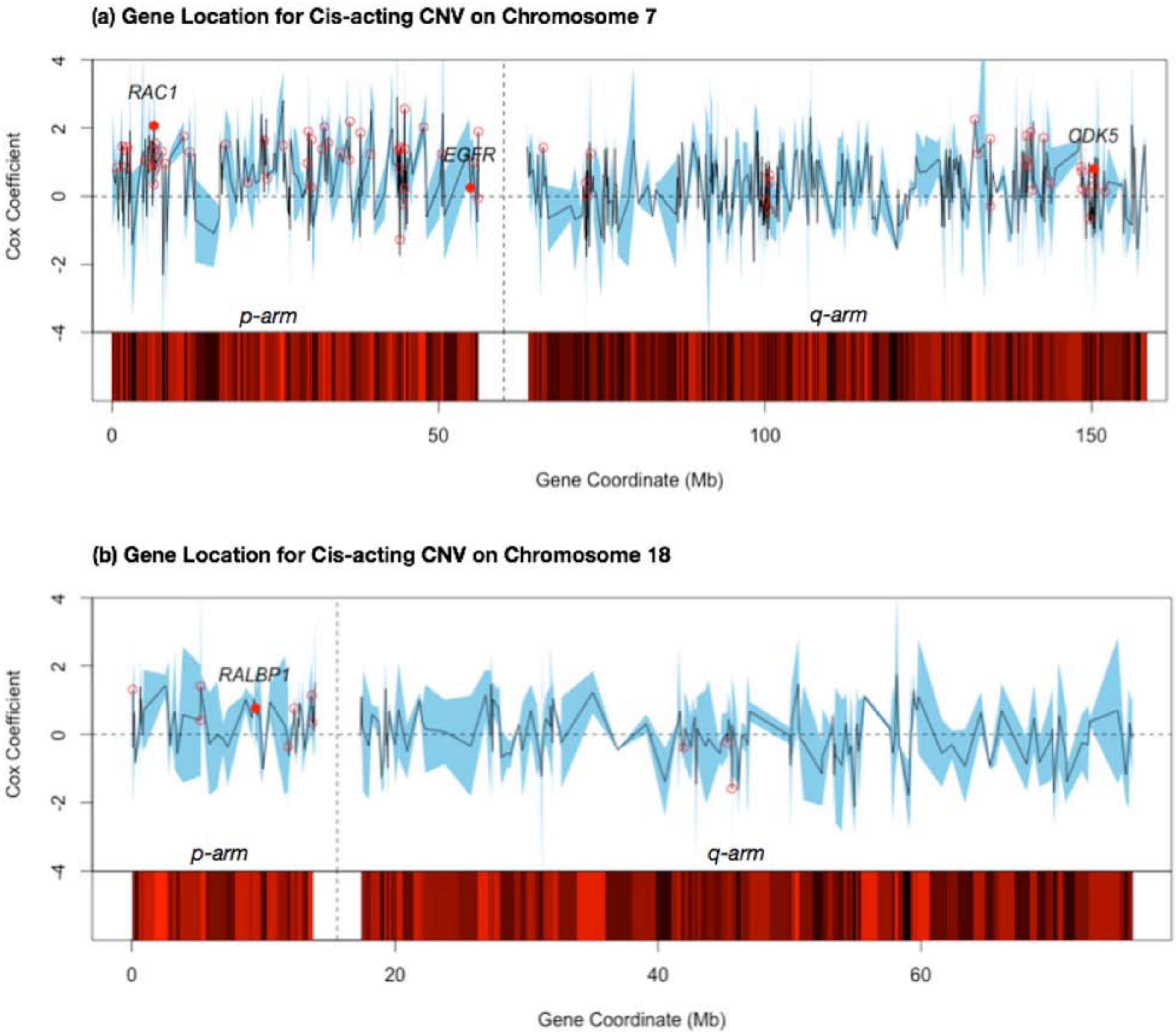

Supplementary Figure S4: Cox regression coefficients from male East Asian datasets of target genes within selected cis-acting CNV modules mapped by the relative locations on the chromosome. The average of the standardized Cox coefficients for the Japanese and Korean male datasets is shown by the black line, with a confidence interval for the two datasets shown by the blue band. Each target gene in the association module is flagged by a hollow red dot. The names of genes with high-ranking PubMed co-citations to lung cancer key terms are highlighted, with the mean Cox coefficient shown with a solid red dot. The p and q arms of each chromosome (i.e. segment boundaries) are separated by blank columns; (a) shows the gene locations for *cis*-acting CNV on chromosome 7; (b) shows the gene locations for *cis*-acting CNV on chromosome 18.

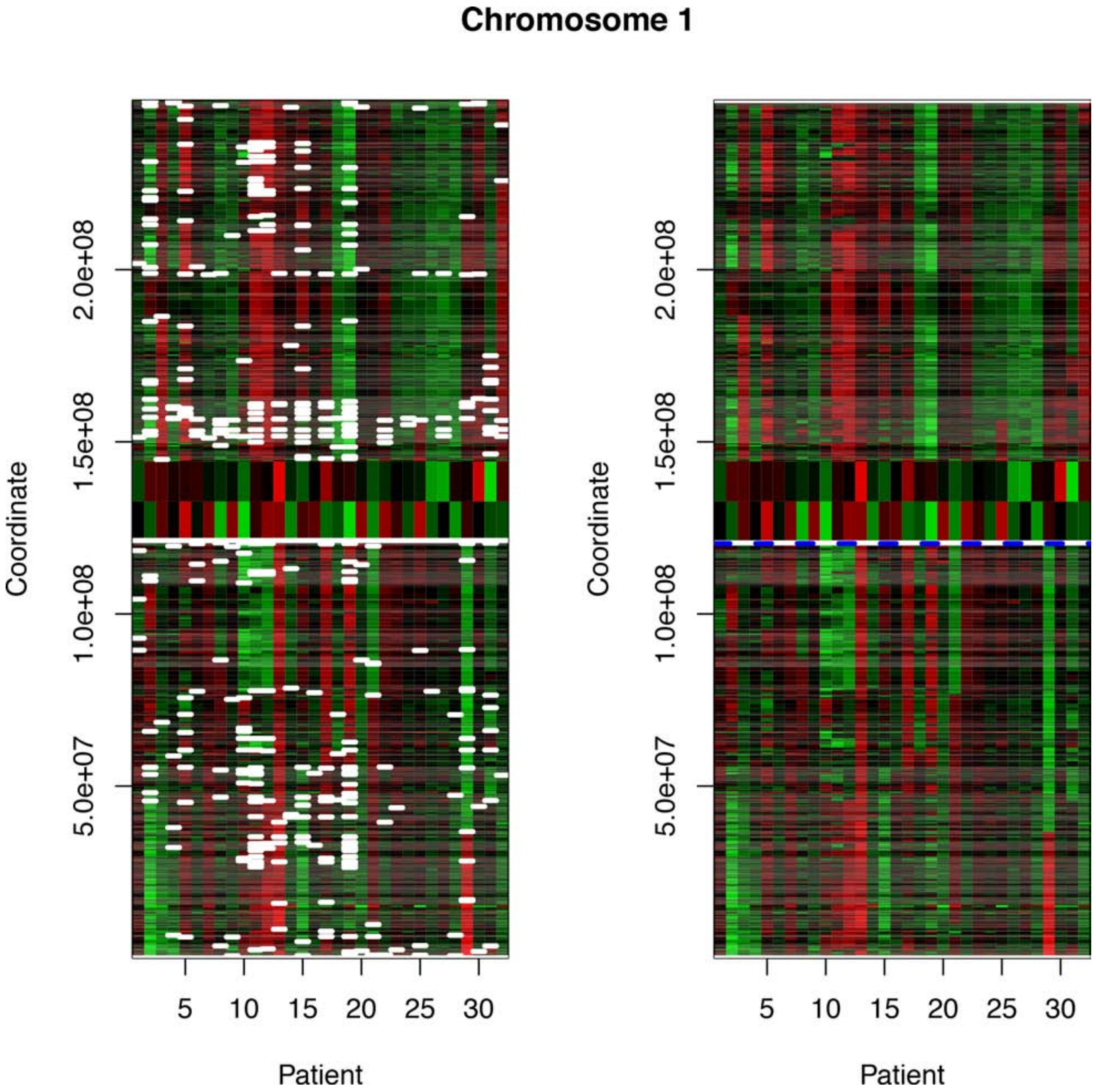
Chromosome 1
Coordinate
2.0e+08
1.5e+08
1.0e+08
5.0e+07
5
10
15
20
25
30
Patient

(*Continued*)

Chromosome 2

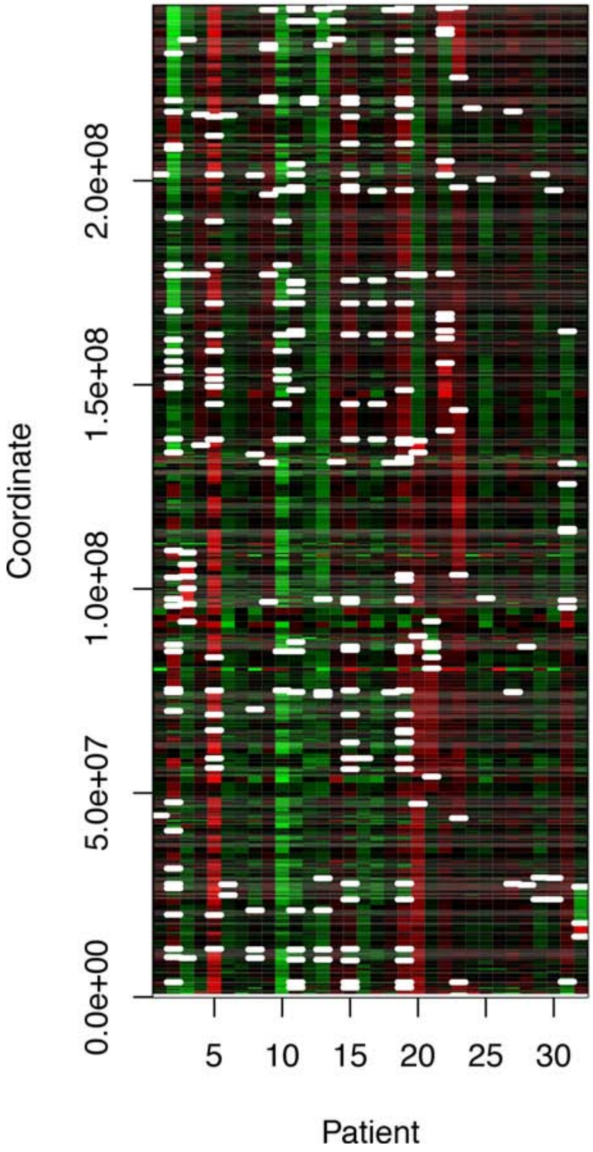

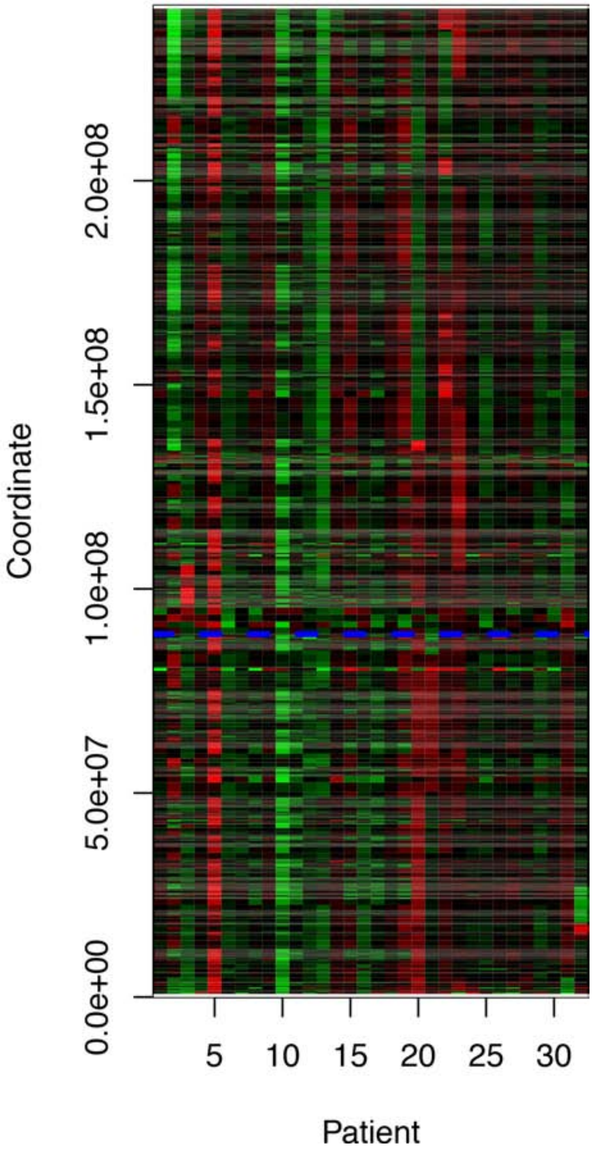

(*Continued*)

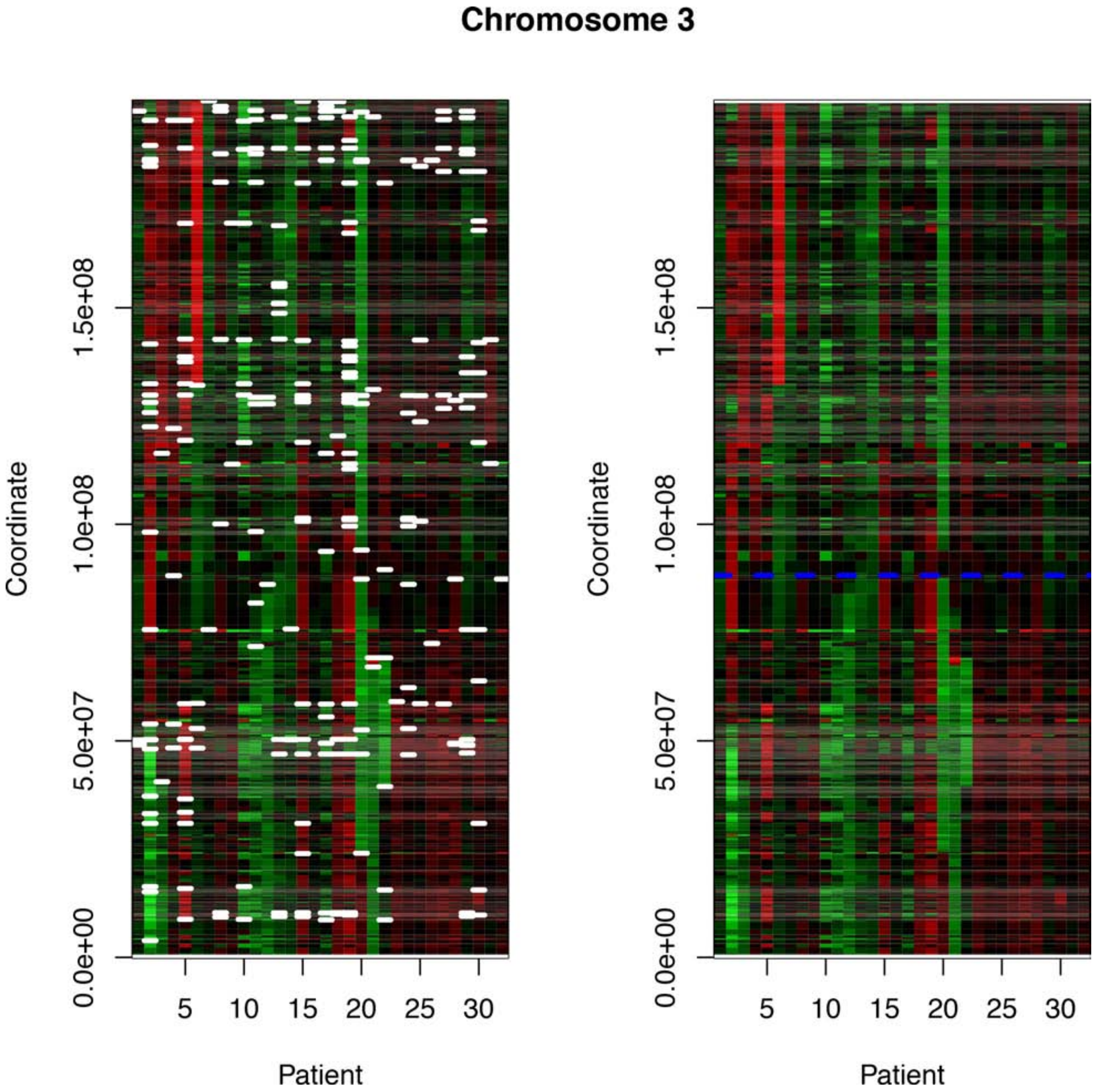
Chromosome 3
Coordinate
0.0e+00
5.0e+07
1.0e+08
1.5e+08
5
10
15
20
25
30
Patient

(*Continued*)

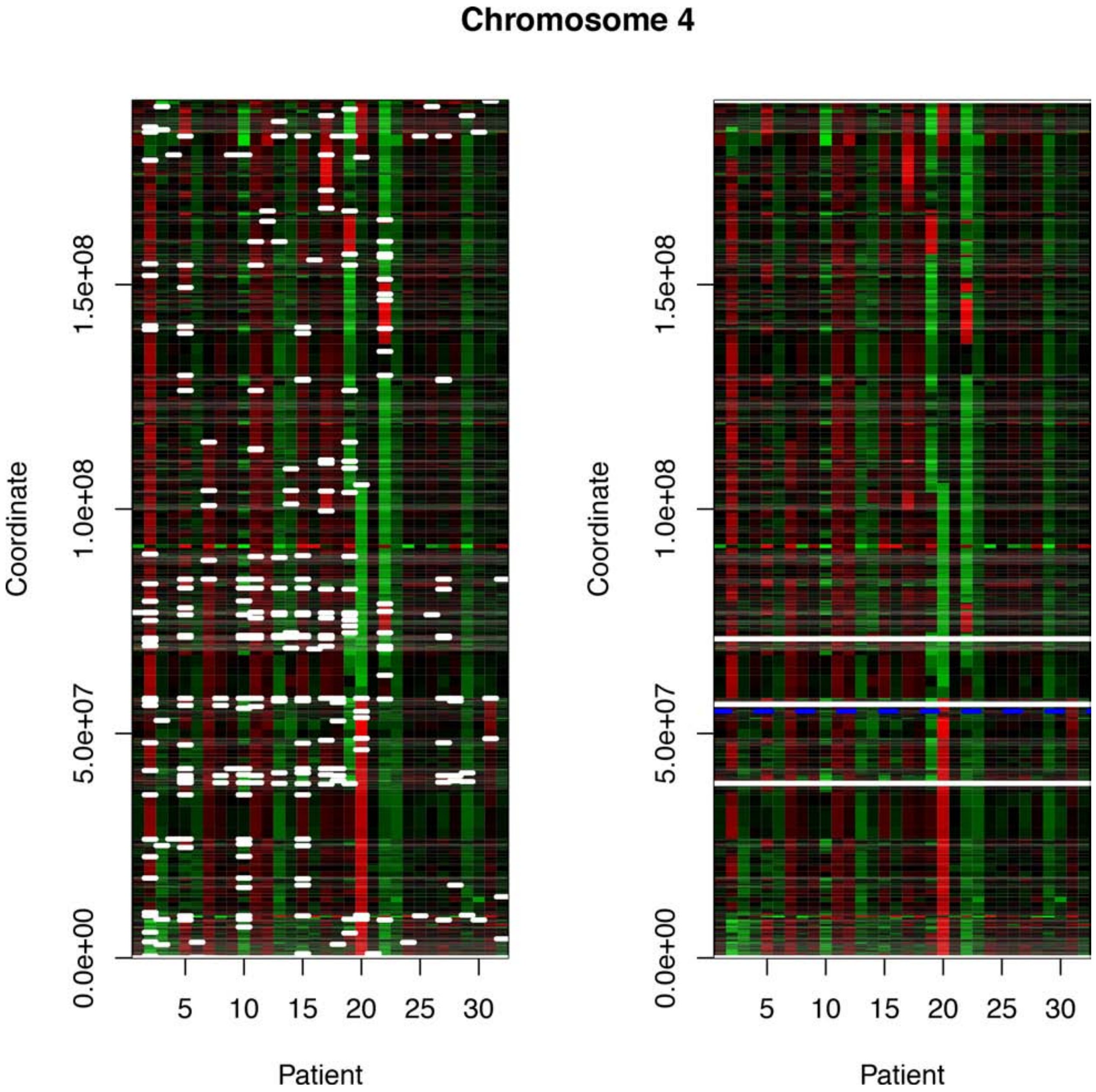
Chromosome 4
Coordinate
0.0e+00
5.0e+07
1.0e+08
1.5e+08
5
10
15
20
25
30
Patient

(*Continued*)

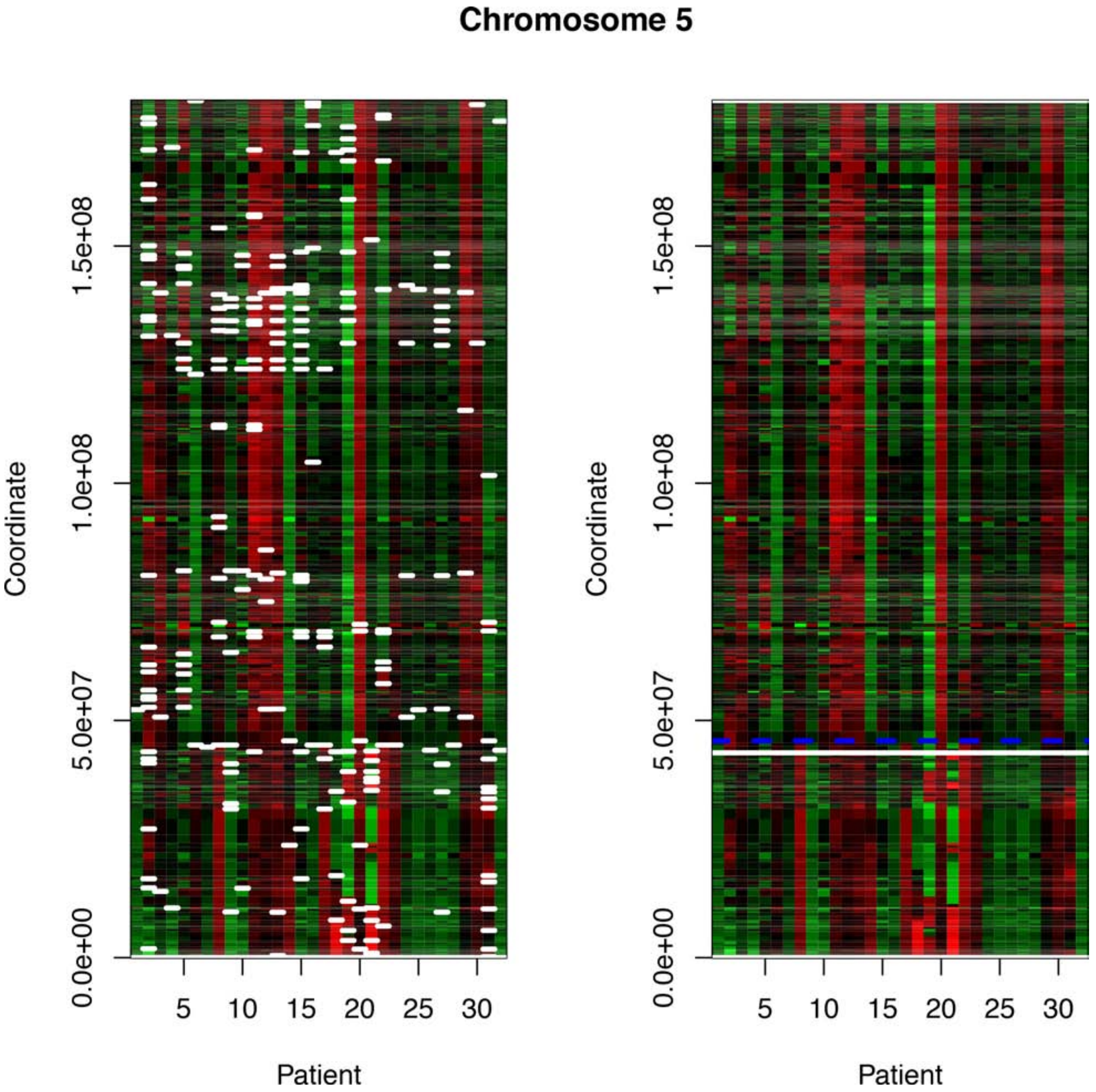
Chromosome 5
Coordinate
0.0e+00
5.0e+07
1.0e+08
1.5e+08
5
10
15
20
25
30
Patient

(*Continued*)

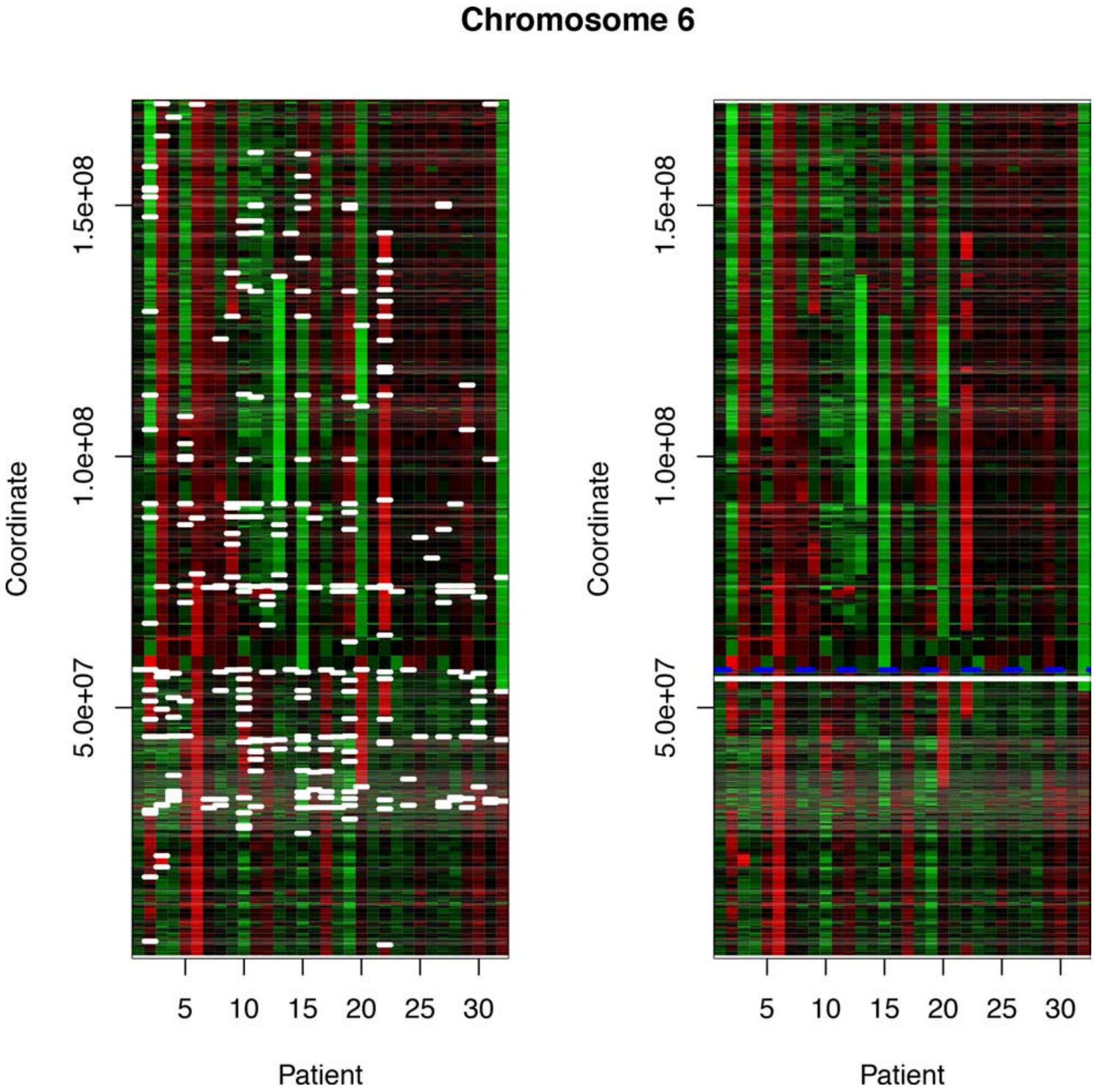
Chromosome 6
Coordinate
1.5e+08
1.0e+08
5.0e+07
5
10
15
20
25
30
Patient

(*Continued*)

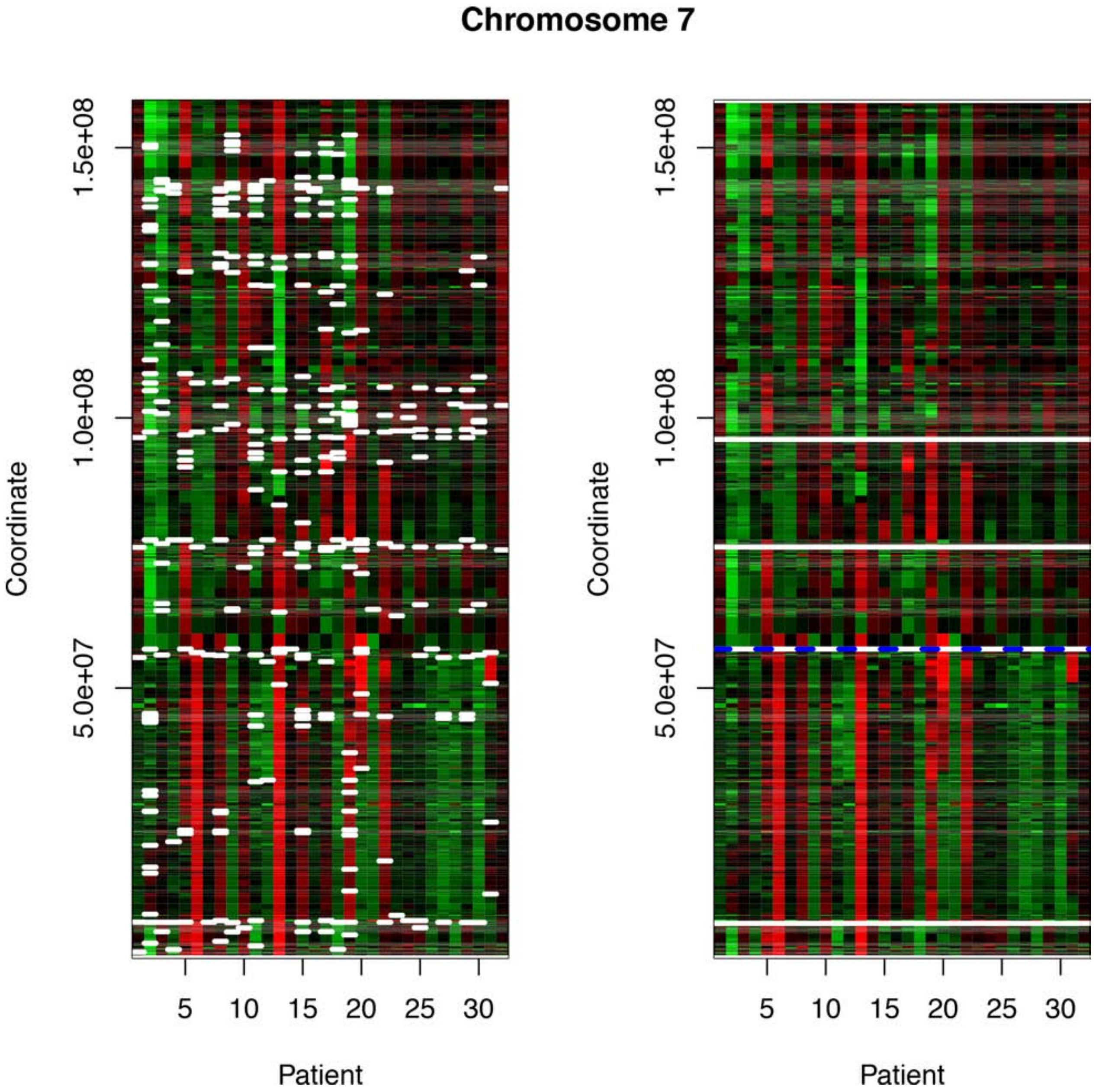
Chromosome 7
1.5e+08
1.0e+08
5.0e+07
Coordinate
5
10
15
20
25
30
Patient

(*Continued*)

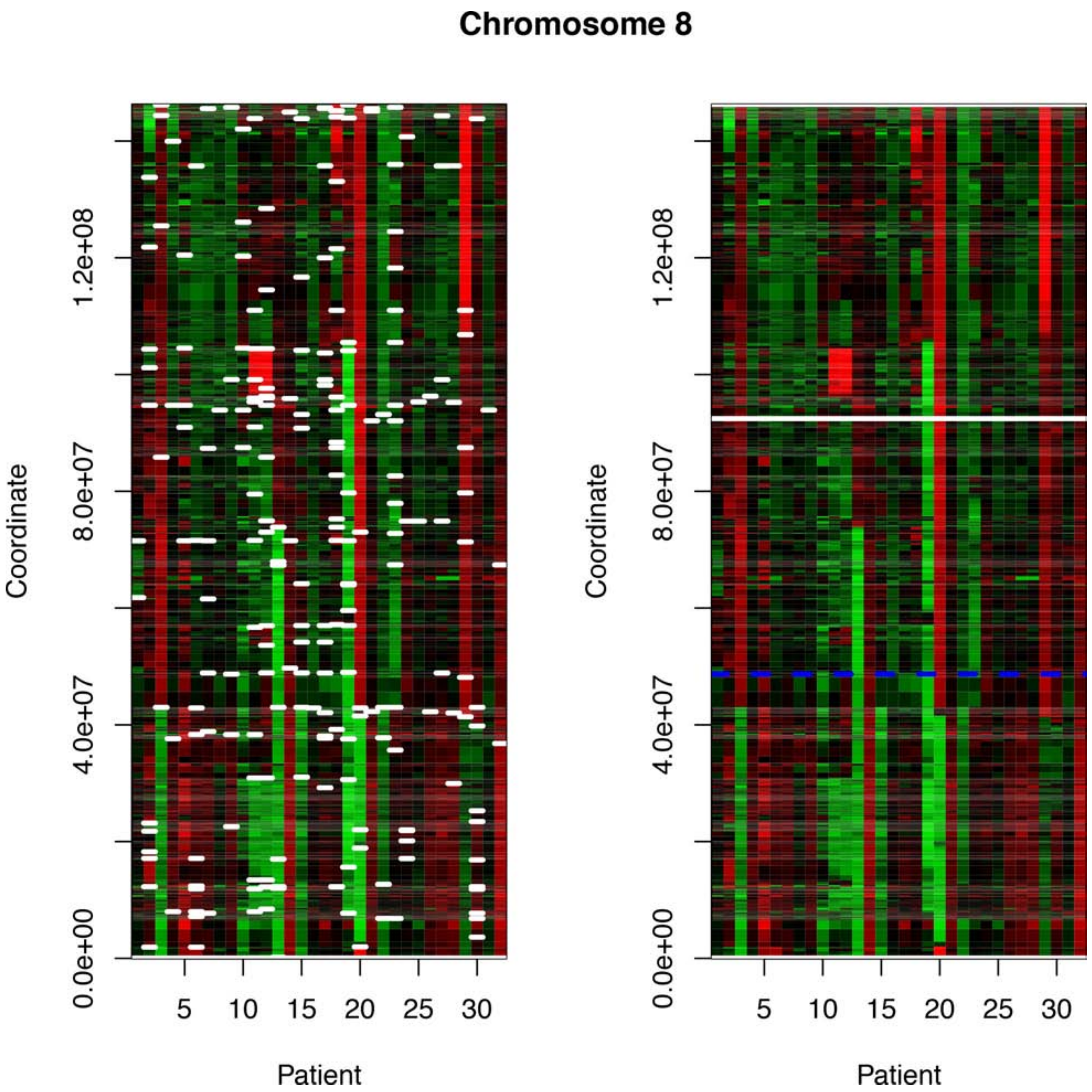
Chromosome 8
Coordinate
0.0e+00
4.0e+07
8.0e+07
1.2e+08
5
10
15
20
25
30
Patient
Coordinate
0.0e+00
4.0e+07
8.0e+07
1.2e+08
5
10
15
20
25
30
Patient

(*Continued*)

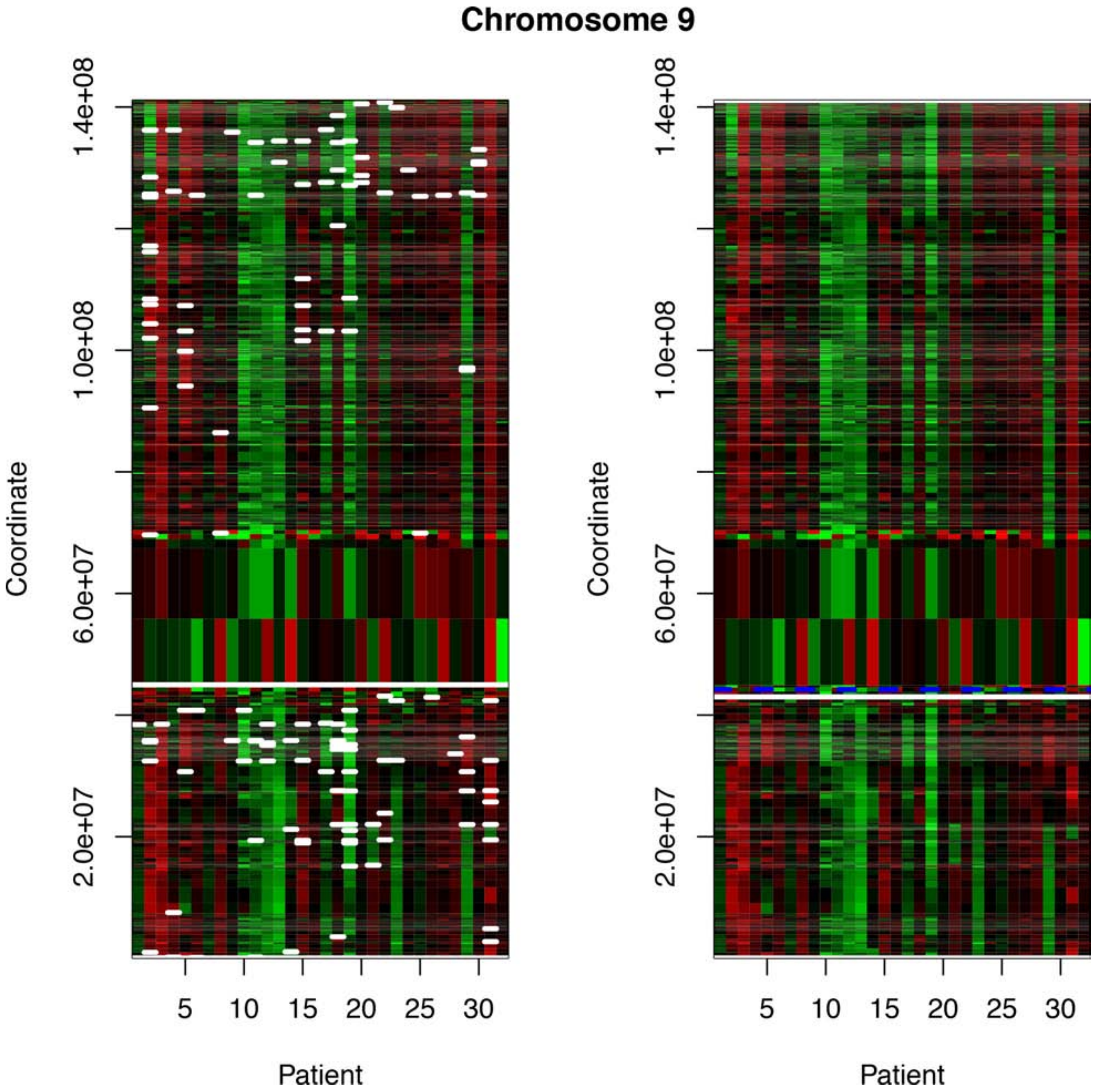
Chromosome 9
Coordinate
1.4e+08
1.0e+08
6.0e+07
2.0e+07
5
10
15
20
25
30
Patient

(Continued)

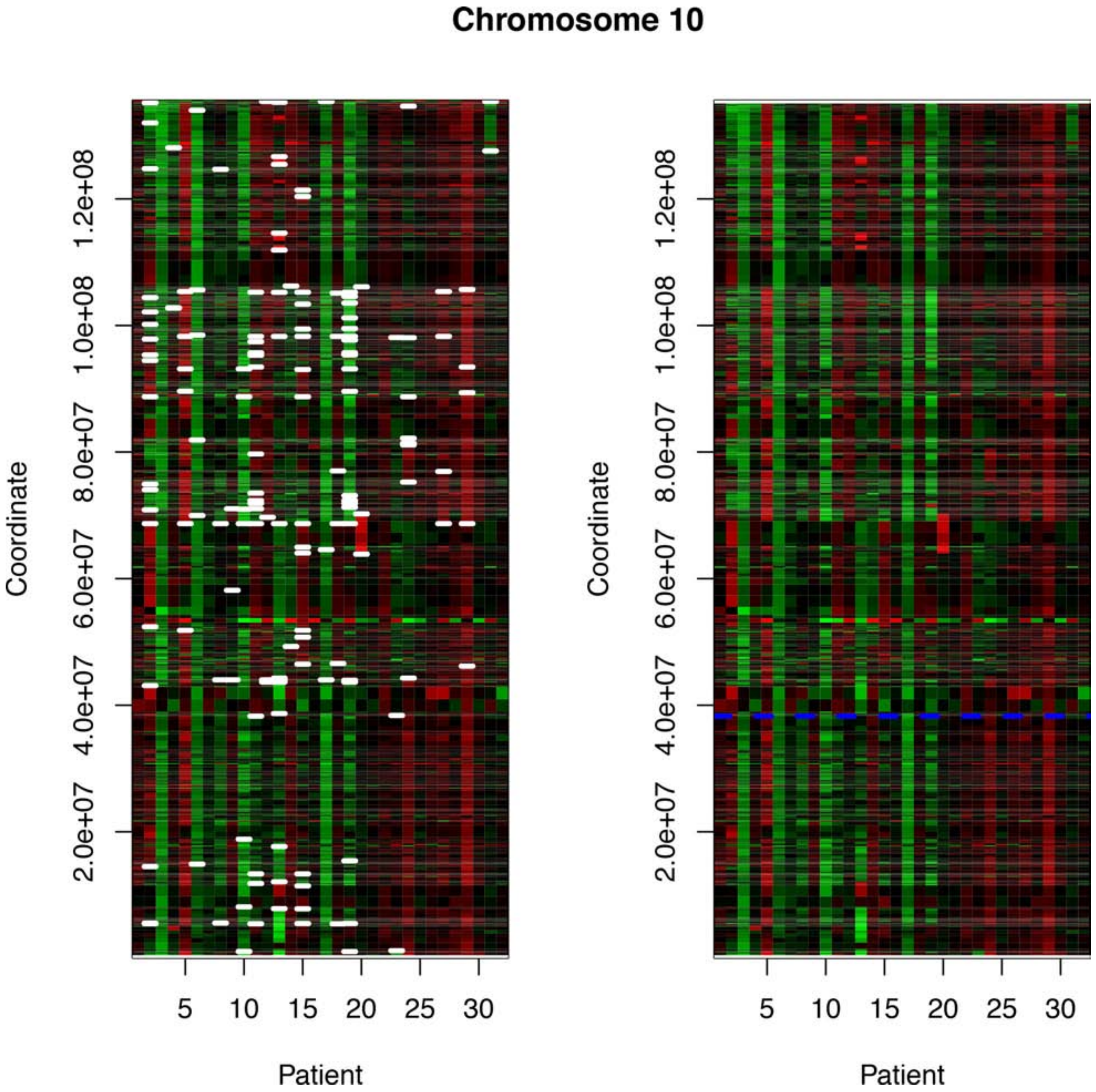
Chromosome 10
Coordinate
1.2e+08
1.0e+08
8.0e+07
6.0e+07
4.0e+07
2.0e+07
5
10
15
20
25
30
Patient
Coordinate
1.2e+08
1.0e+08
8.0e+07
6.0e+07
4.0e+07
2.0e+07
5
10
15
20
25
30
Patient

(Continued)

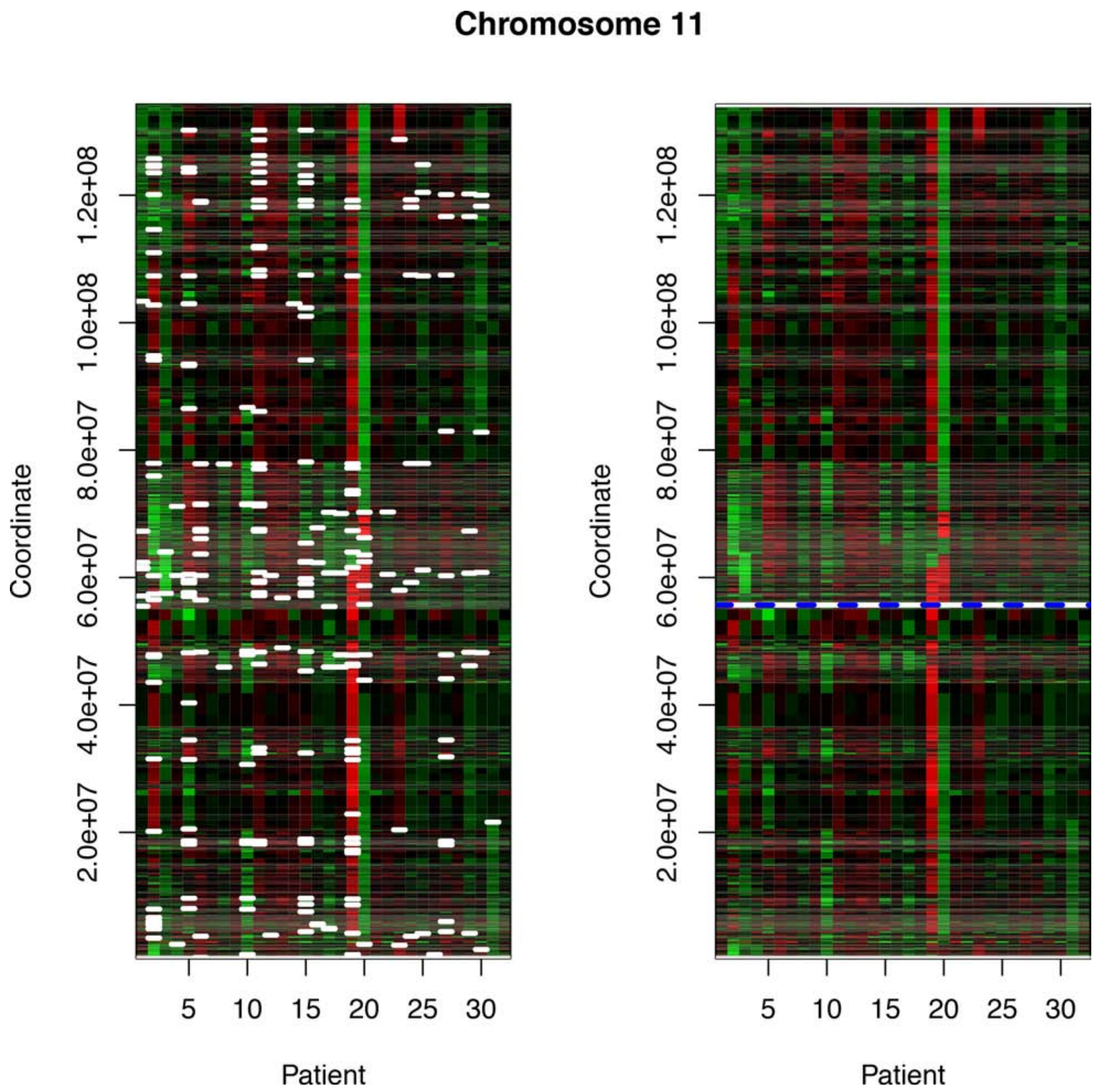
Chromosome 11
Coordinate
1.2e+08
1.0e+08
8.0e+07
6.0e+07
4.0e+07
2.0e+07
5
10
15
20
25
30
Patient
Coordinate
1.2e+08
1.0e+08
8.0e+07
6.0e+07
4.0e+07
2.0e+07
5
10
15
20
25
30
Patient

(*Continued*)

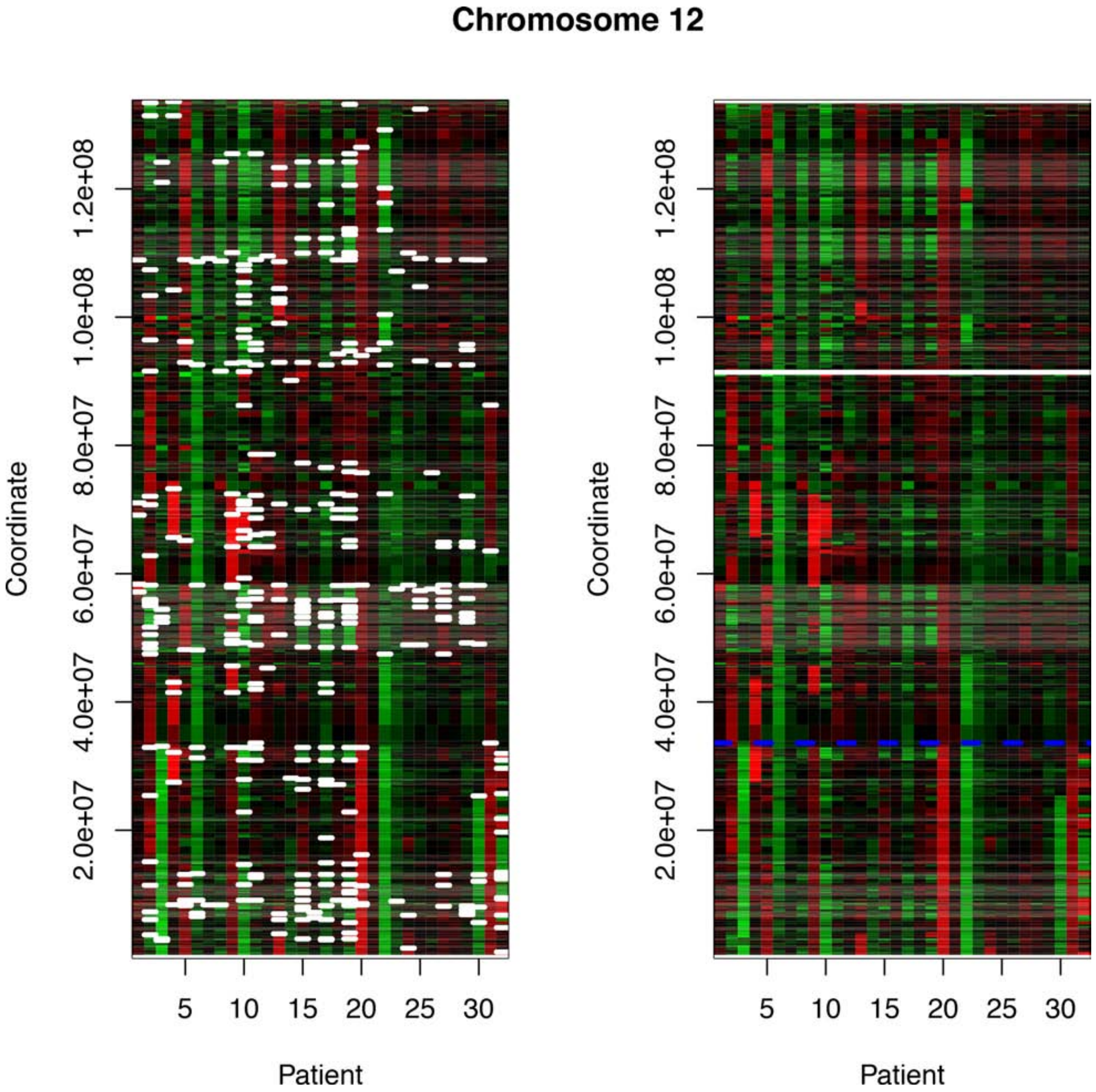
Chromosome 12
Coordinate
1.2e+08
1.0e+08
8.0e+07
6.0e+07
4.0e+07
2.0e+07
5
10
15
20
25
30
Patient

(*Continued*)

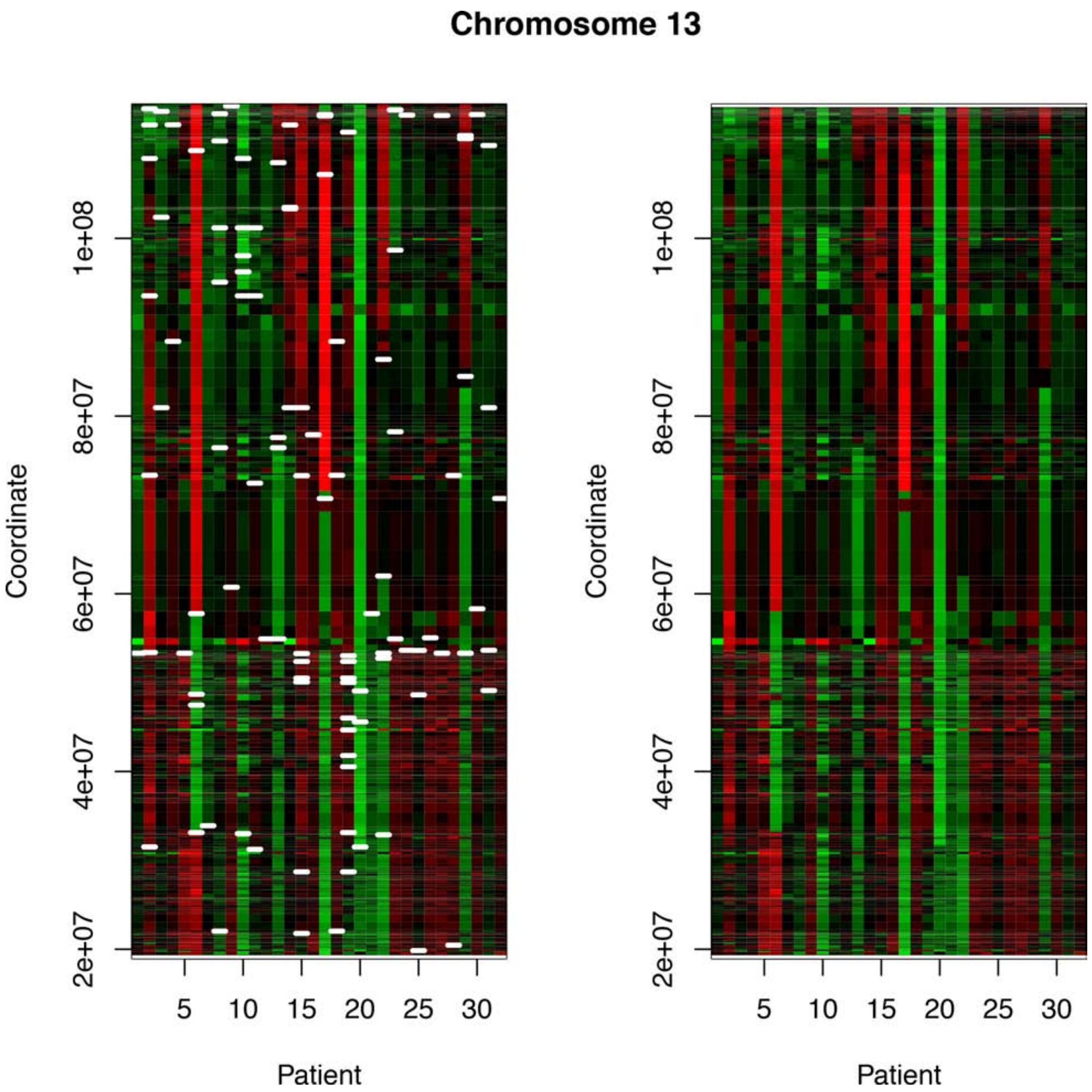

Chromosome 13
Coordinate
1e+08
8e+07
6e+07
4e+07
2e+07
5
10
15
20
25
30
Patient

(*Continued*)

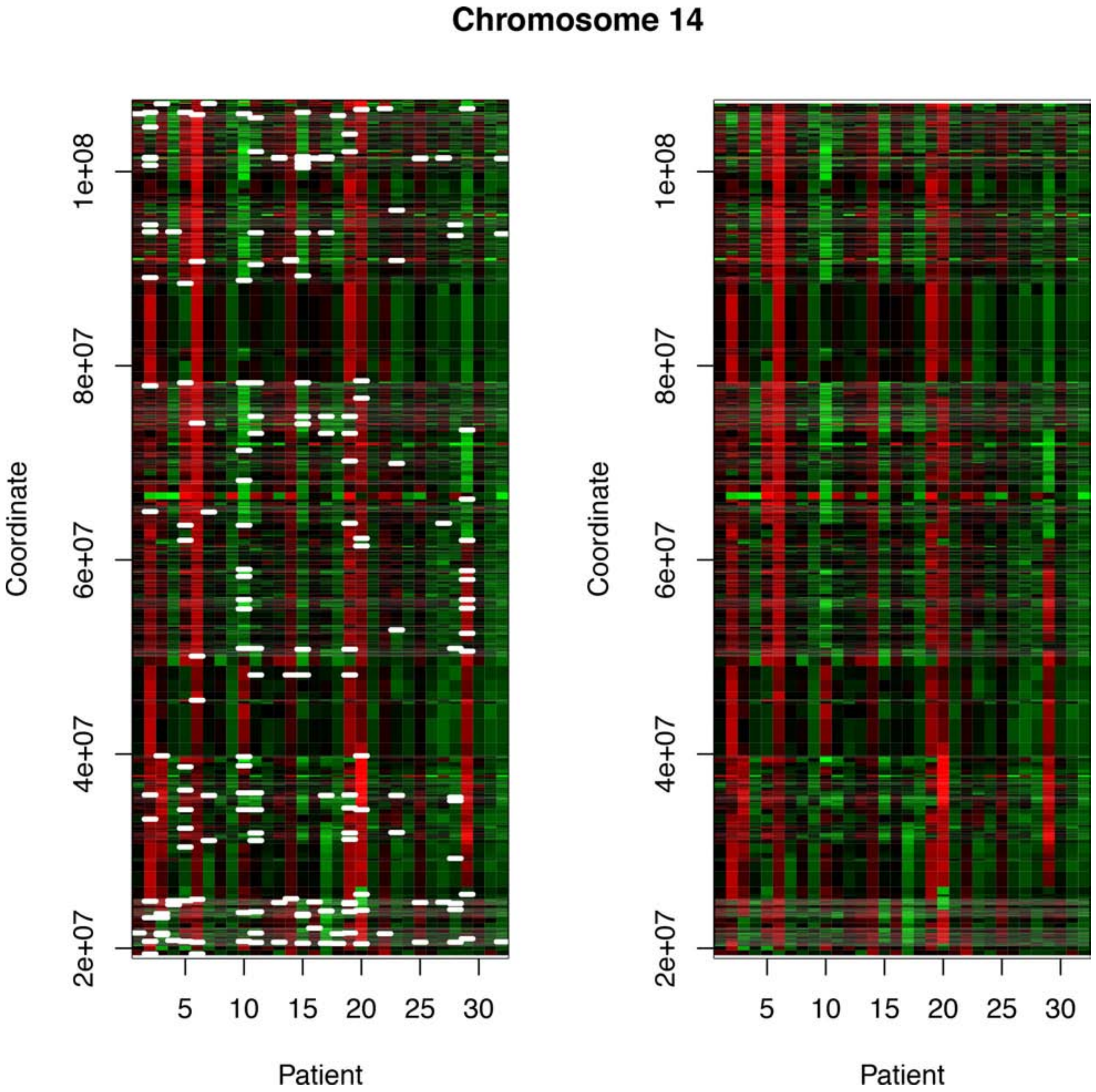
Chromosome 14
Coordinate
1e+08
8e+07
6e+07
4e+07
2e+07
5
10
15
20
25
30
Patient
Coordinate
1e+08
8e+07
6e+07
4e+07
2e+07
5
10
15
20
25
30
Patient

(*Continued*)

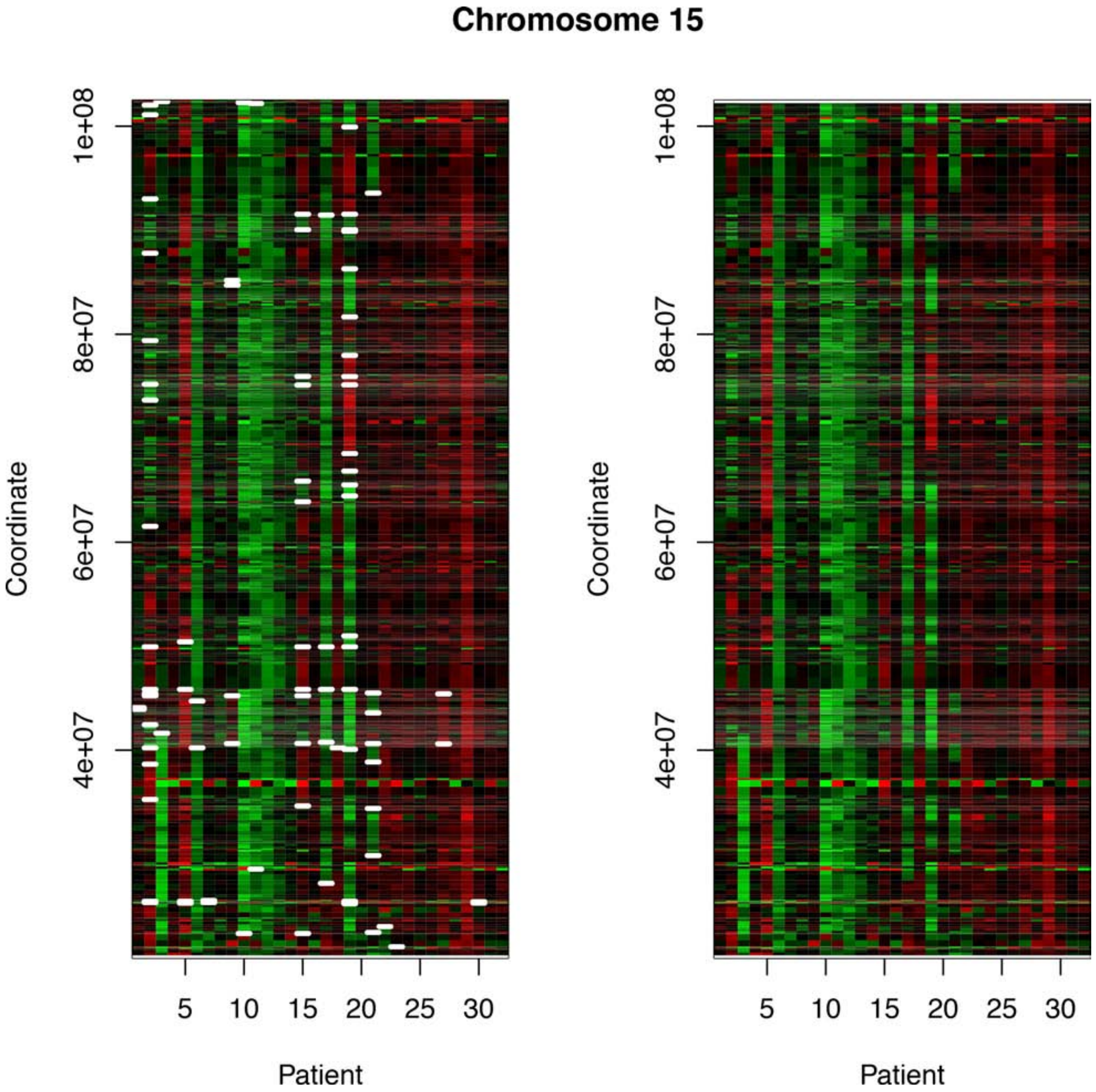
Chromosome 15
Coordinate
1e+08
8e+07
6e+07
4e+07
Patient
5
10
15
20
25
30

(*Continued*)

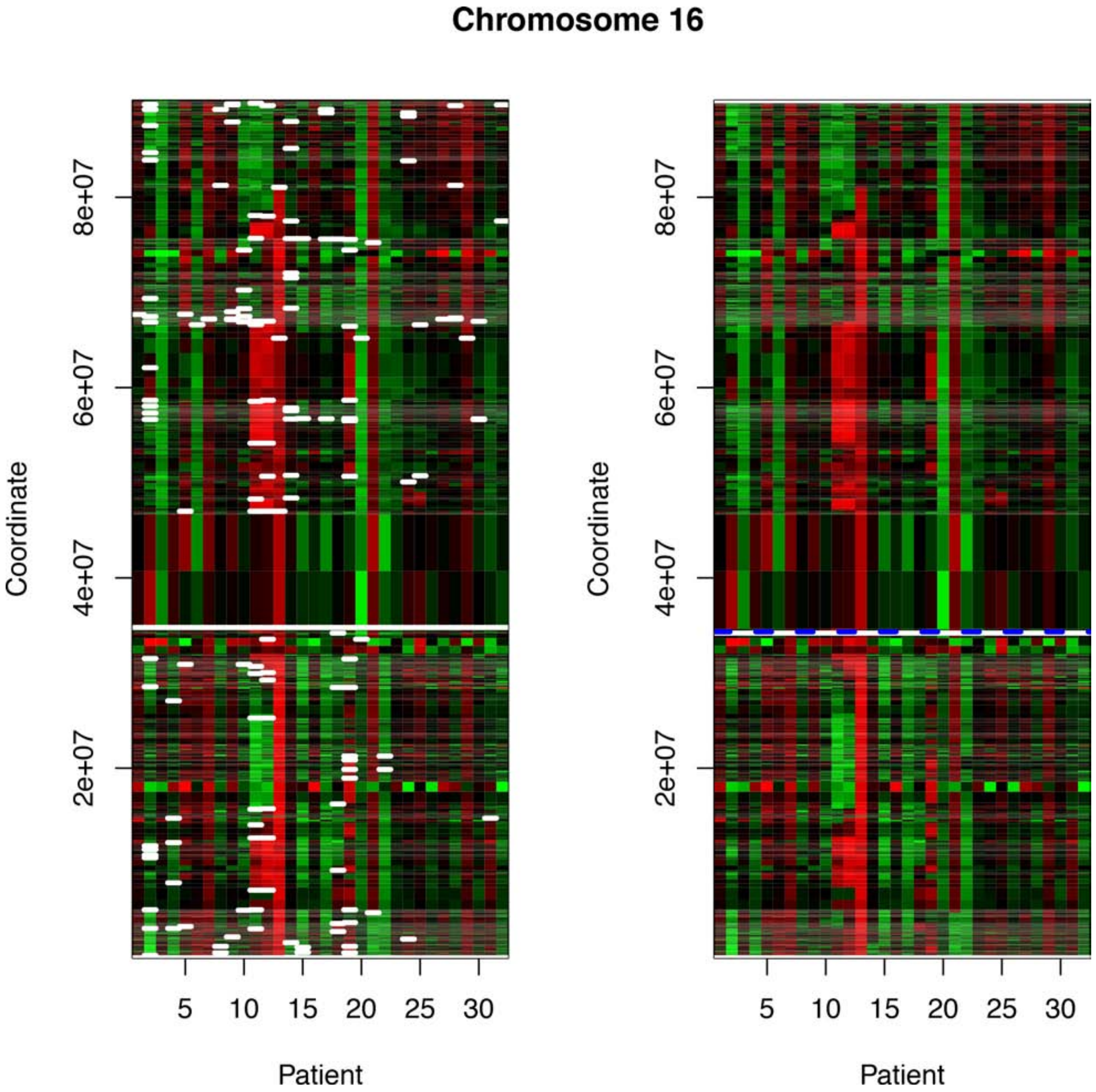
Chromosome 16
Coordinate
8e+07
6e+07
4e+07
2e+07
5
10
15
20
25
30
Patient

(*Continued*)

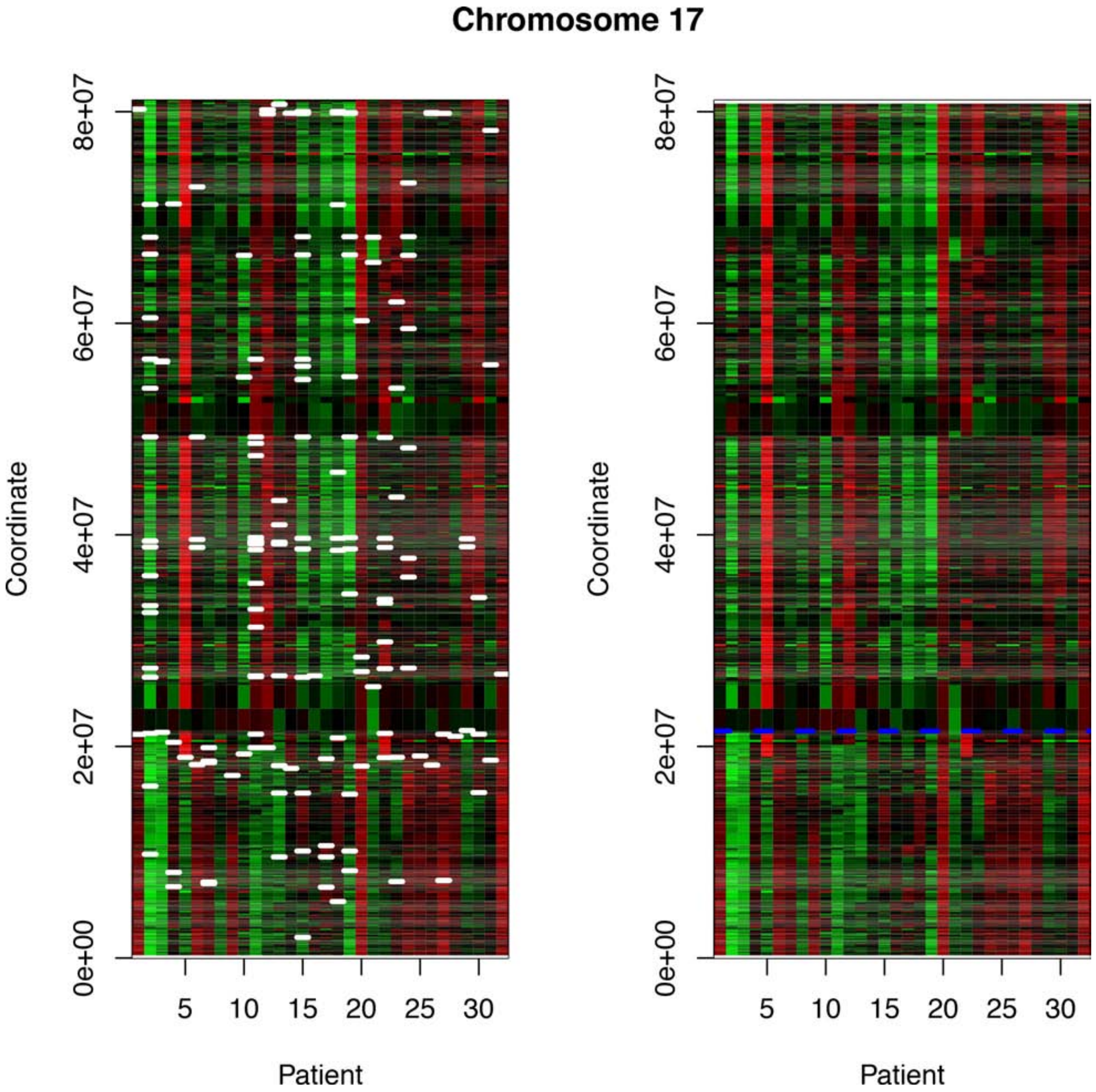
Chromosome 17
Coordinate
8e+07
6e+07
4e+07
2e+07
0e+00
5
10
15
20
25
30
Patient

(*Continued*)

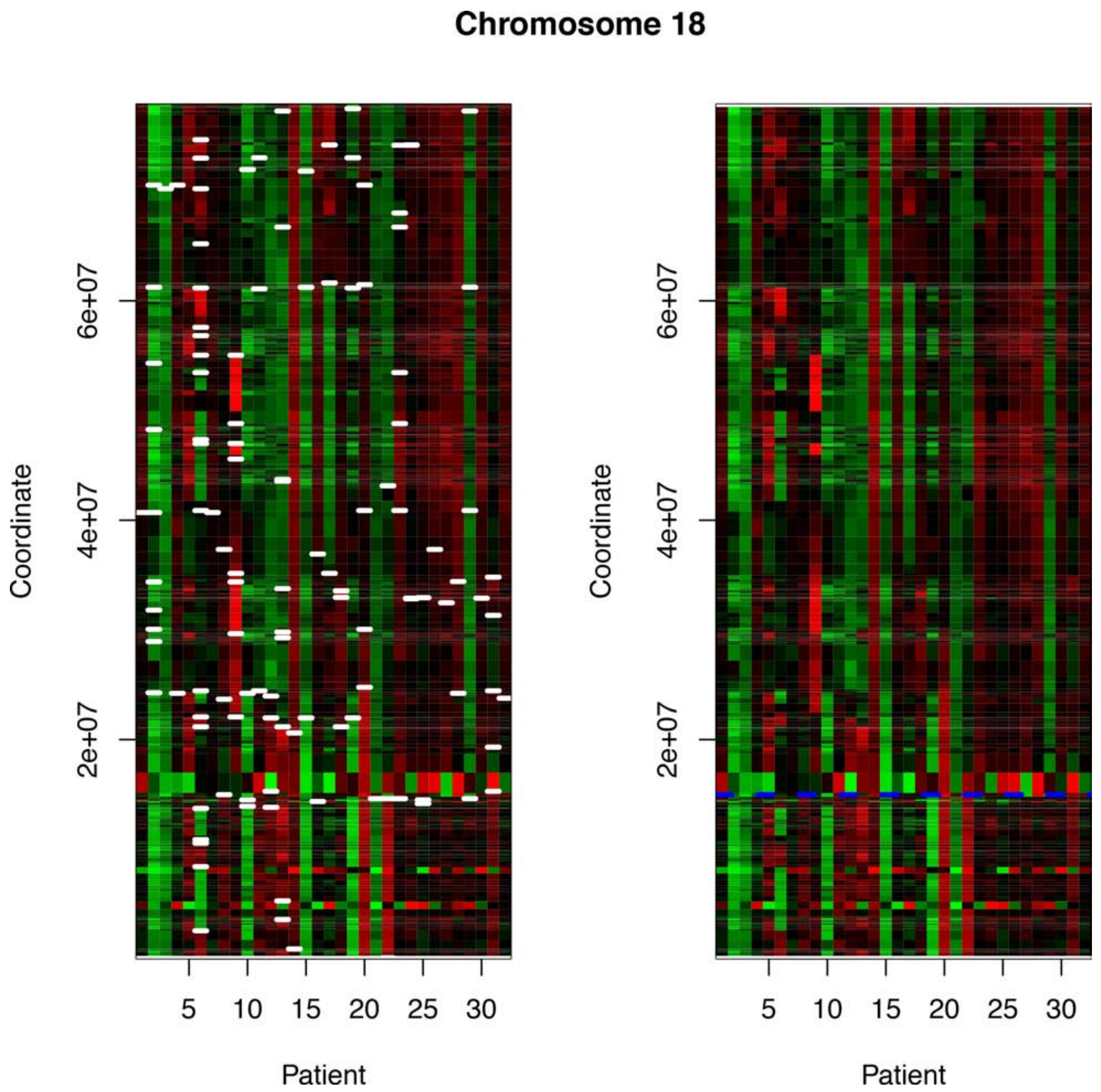
Chromosome 18
Coordinate
6e+07
4e+07
2e+07
5
10
15
20
25
30
Patient
Coordinate
6e+07
4e+07
2e+07
5
10
15
20
25
30
Patient

(*Continued*)

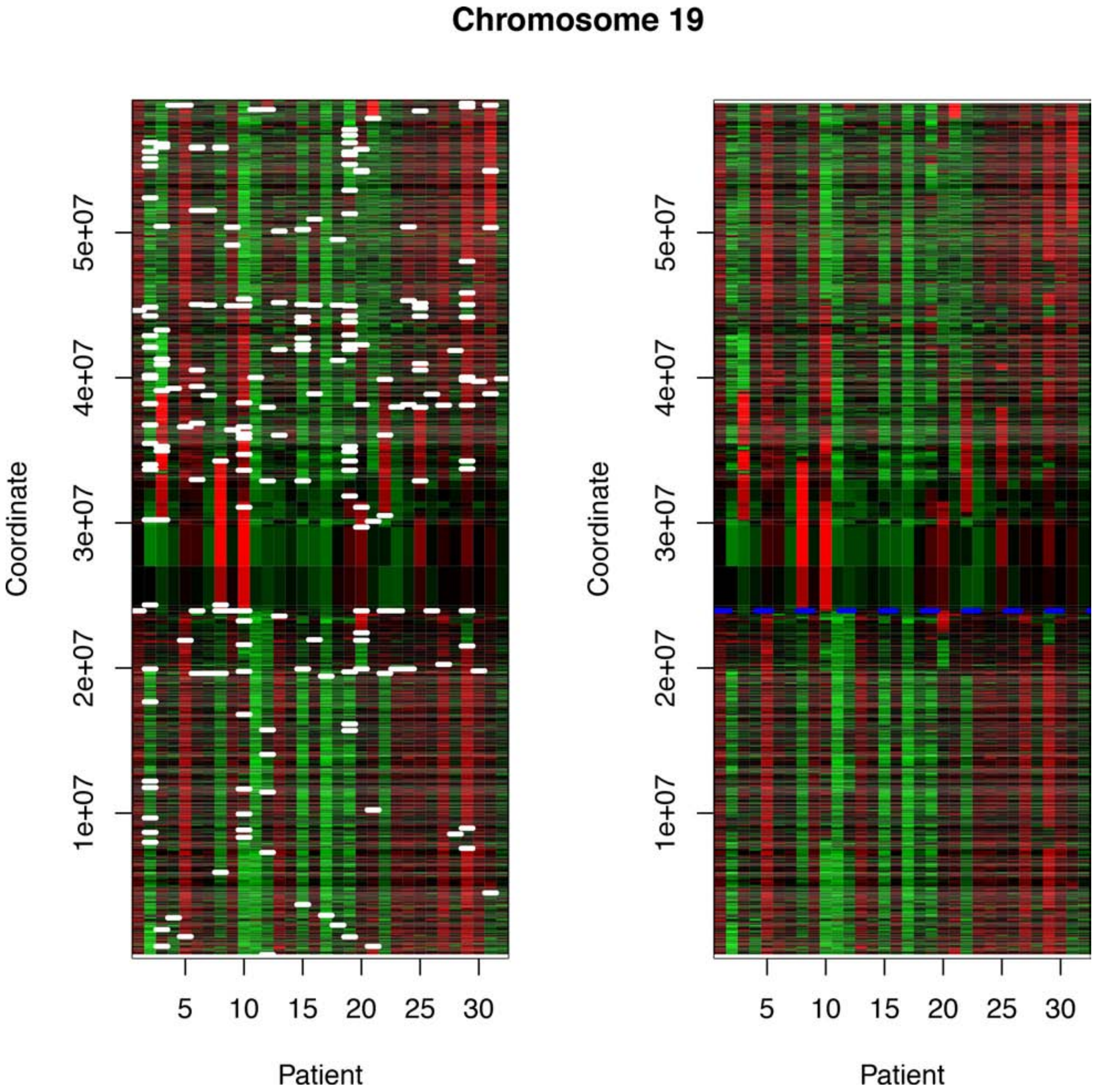
Chromosome 19
Coordinate
5e+07
4e+07
3e+07
2e+07
1e+07
5
10
15
20
25
30
Patient

(*Continued*)

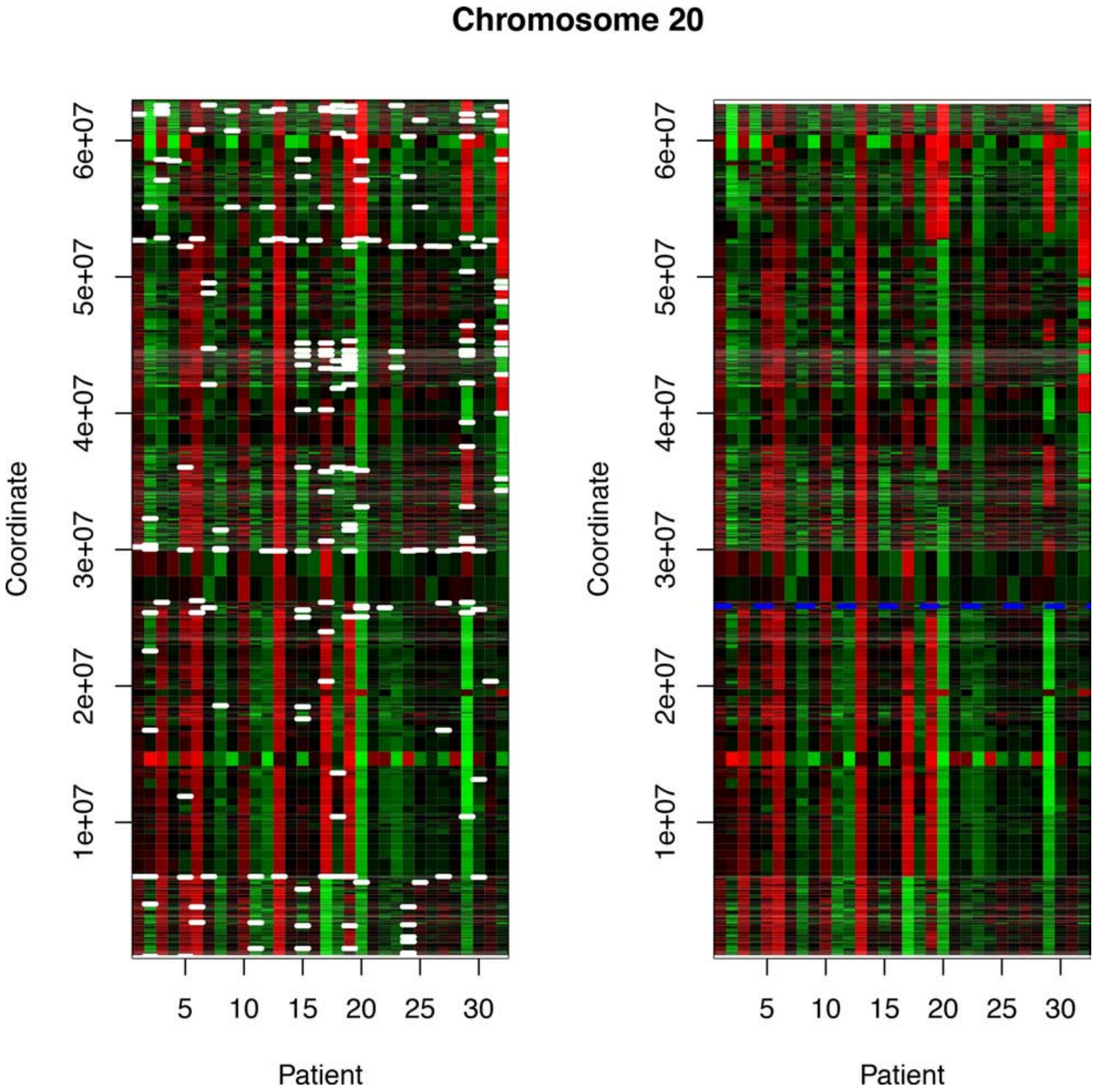
Chromosome 20
Coordinate
6e+07
5e+07
4e+07
3e+07
2e+07
1e+07
5
10
15
20
25
30
Patient

(*Continued*)

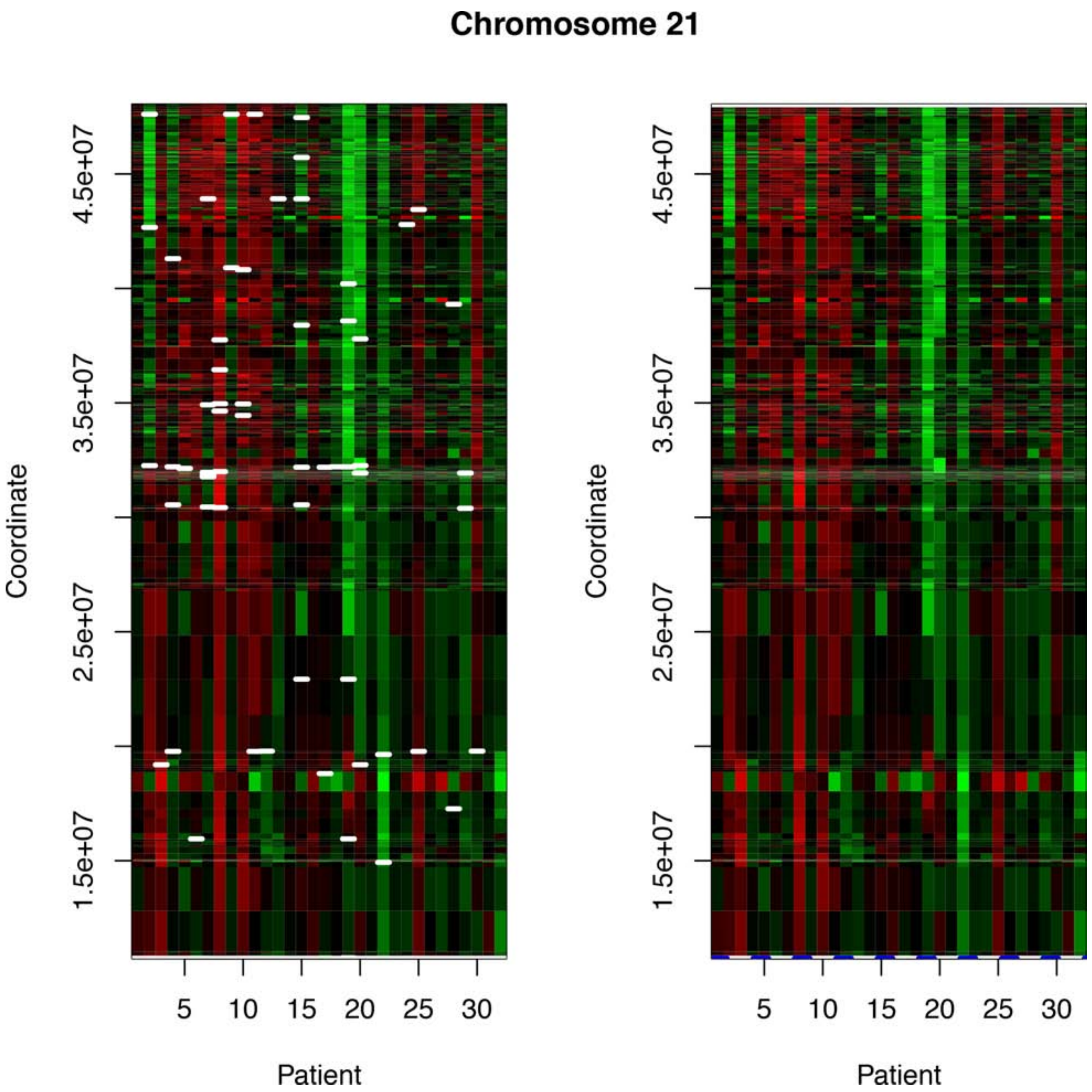
Chromosome 21
Coordinate
4.5e+07
3.5e+07
2.5e+07
1.5e+07
5
10
15
20
25
30
Patient
Coordinate
4.5e+07
3.5e+07
2.5e+07
1.5e+07
5
10
15
20
25
30
Patient

(*Continued*)

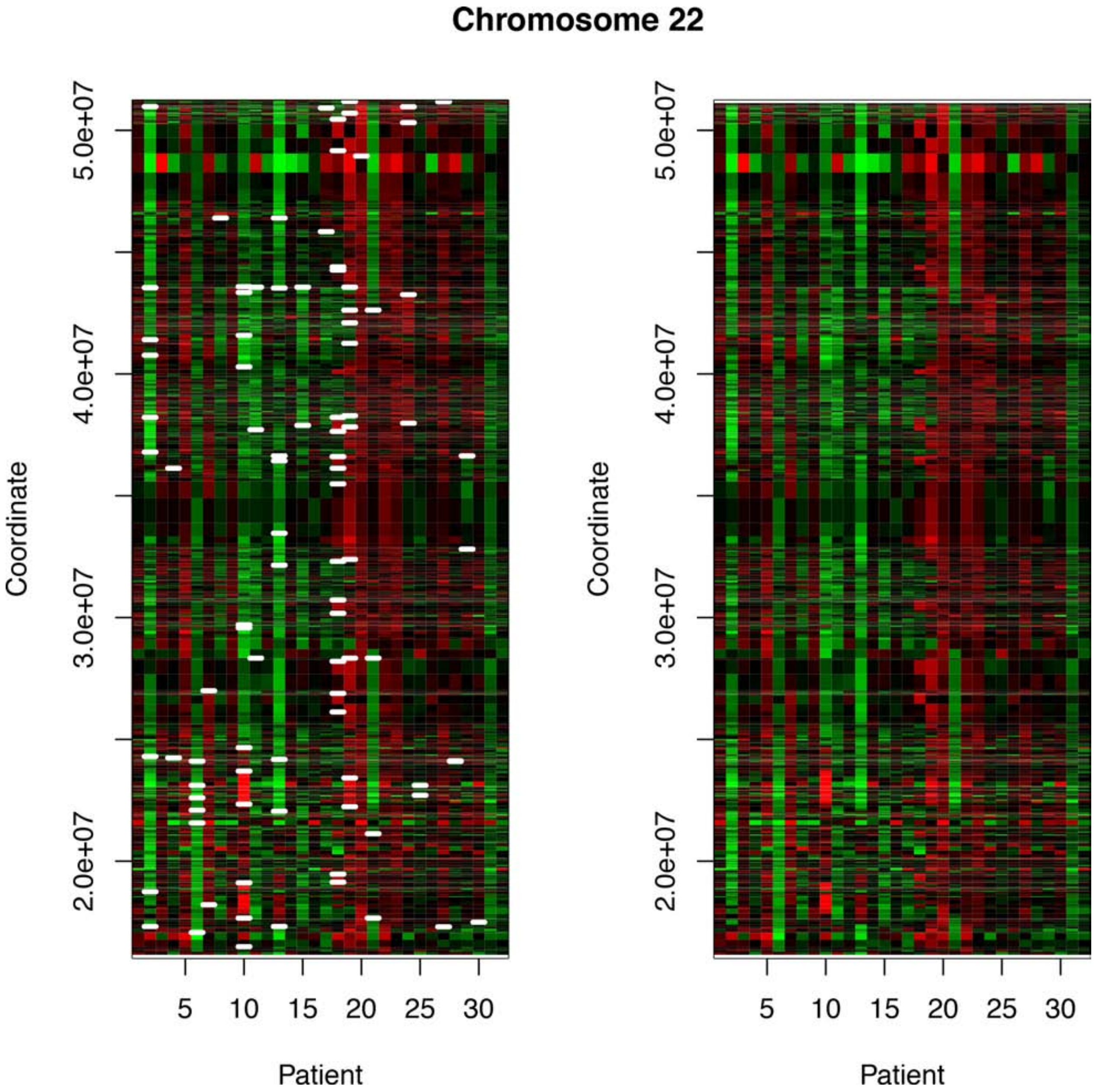
Chromosome 22
Coordinate
5.0e+07
4.0e+07
3.0e+07
2.0e+07
5
10
15
20
25
30
Patient

(*Continued*)

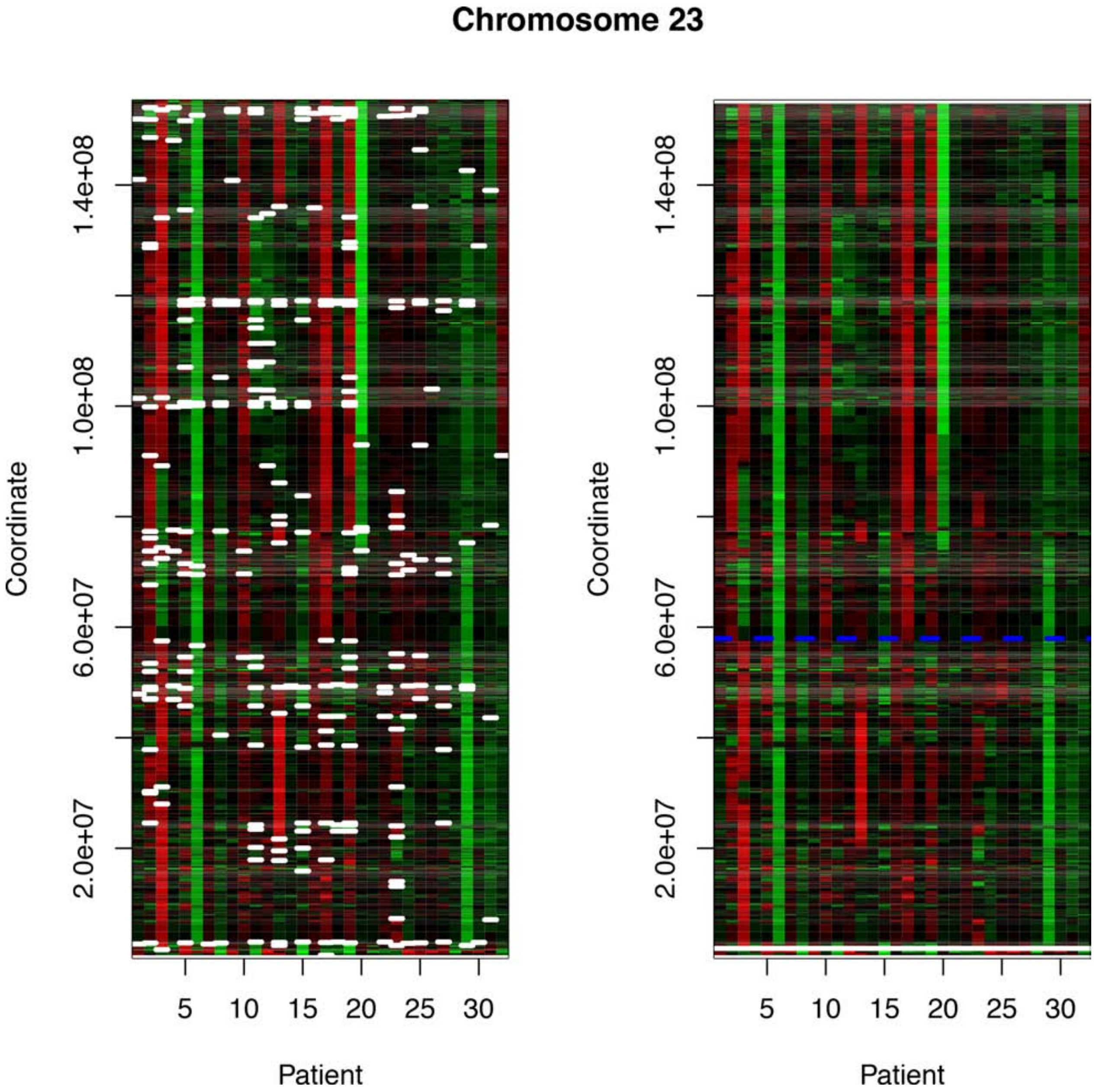

Supplementary Figure S5: Validation of segment boundaries by the circular binary algorithm. Heatmaps of normalized CNV data for each chromosome illustrate the segment positions on the individual patients generated by the circular binary algorithm (left) and the merged complete boundaries across all observations (right). For the heatmaps of complete boundaries we illustrate the p and q arm segmentation boundaries employed in the study (blue dashed line). The white solid lines illustrate the positions of the segment boundaries inferred by the circular binary segments.

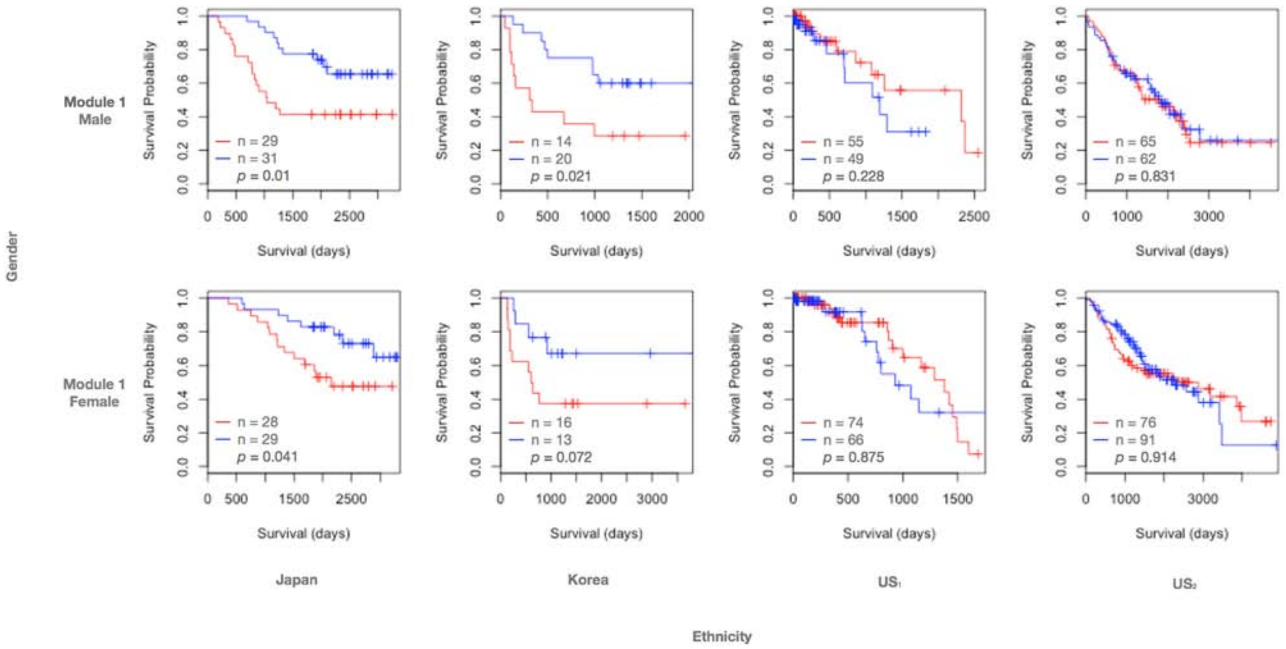

Supplementary Figure S6: Kaplan-Meier curves of cis-acting CNV on chromosome 7 using Circular Binary Segmentation partitions. Kaplan-Meier survival curves of patients divided by median gene expression amongst target genes of the chromosome 7 *cis*-acting CNV module defined by the Circular Binary Segmentation algorithm. A red line indicates the survival curve of the patient group with high median expression levels in the target genes. A blue line indicates the survival curve of the patient group with low median expression levels in the target genes. Tick marks indicate censored data points; *p*-values are determined by log-rank tests. The size of each patient group and the log-rank *p*-value are reported.

Supplementary Table S1. Summary of Association Modules. A summary of the association modules generated in the study. Each module consists of three components: (i) an observed effector molecular aberration on DNA (*cis*-acting CNV, *trans*-acting CNV or methylation); (ii) the downstream target genes with expression profiles associated with the effector molecular aberration; and (iii) regulators (transcription factors or signalling proteins) that mediate the effects between effector molecular aberrations and target gene expressions.

module 1	effector 1, intra segment CNV, chromosome 7<br>84 targets: HEATR2 PSMG3 FTSJ2 NUDT1 EIF3B C7ORF27 RBAK AIMP2 EIF2AK1 C7ORF70 RAC1 DAGLB KDELR2 ZDHHC4 C7ORF26 ZNF12 MIOS RPA3 ICA1 PHF14 TMEM106B SNX13 SP4 C7ORF30 STK31 CBX3 SCRN1 PLEKHA8 GGCT GARS LSM5 AVL9 LOC401321 BBS9 DPY19L1 KIAA0895 ANLN STARD3NL C7ORF36 MRPS24 DBNL POLM POLD2 YKT6 TMED4 ZMIZ2 PPIA PURB HUS1 FIGNL1 EGFR LANCL2 PSPH CCT6A CHCHD2 C7ORF42 BAZ1B ABHD11 RFC2 MOSPD3 ZNHIT1 FIS1 RABL5 CHCHD3 EXOC4 C7ORF49 WDR91 ADCK2 NDUFB2 MRPS33 AGK KIAA1147 CASP2 ARHGEF5 ZNF786 ZNF282 ZNF212 LOC401431 ZNF775 ATG9B CDK5 SLC4A2 FASTK ACTR3B
module 2	effector 1, intra segment CNV, chromosome 18<br>12 targets: USP14 MYL12B C18ORF18 LOC339290 RALBP1 MPPE1 AFG3L2 C18ORF19 RNMT ATP5A1 RPL17 SCARNA17
module 3	effector 1, methylation UBIAD1 VAV1, negative,<br>87 targets: UBIAD1 CDC20 KIF2C NUF2 CENPL KIF14 RRM2 E2F6 GEN1 TP53I3 ZC3H8 SPC25 FASTKD1 MARS2 PTH2R ITM2C NCL HJURP KBTBD5 SOX14 RNF4 NCAPG CCNA2 MND1 LMNB1 KIF20A CDX1 C5ORF40 PTTG1 HMMR PSORS1C2 TUBB2B HIST1H3B C6ORF129 BYSL PRSS2 EZH2 LOC389641 CDCA2 PBK IFNA1 SOHLH1 TET1 KIF11 CEP55 MKI67 GYLTL1B KRTAP5-9 FAM55D THY1 FOXM1 CDCA3 PRB4 FKBP11 TROAP ESPL1 C12ORF45 C13ORF23 C13ORF34 SLC39A2 CDKN3 DLGAP5 NIPA2 MAGEL2 BLM CDYL2 FAM64A SLC25A39 KIF18B KPNA2 TK1 PYCR1 TAF4B SERPINB12 CST1 TPX2 BASE MYBL2 UBE2C COL9A3 MTP18 CENPM GLRA2 PAGE4 IL1RAPL2 KIAA1210 UBE2NL
module 4	effector 1, intra segment CNV, chromosome 1<br>92 targets: TMEM52 ARHGEF16 C1ORF135 AKIRIN1 SPATA6 PARS2 TM2D1 ENSA SAMD13 MCOLN3 C1ORF52 SORT1 CSDE1 NUDT17 POLR3C CHD1L ACP6 SF3B4 SETDB1 PRUNE C1ORF56 LYSMD1 VPS72 PSMD4 PI4KB PSMB4 MRPL9 C1ORF43 UBAP2L HAX1 UBE2Q1 ADAR FLAD1 EFNA4 MTX1 SCAMP3 SSR2 RAB25 MEX3A CCT3 APOA1BP HDGF PRCC IGSF9 PIGM USP21 NDUFS2 SDHC C1ORF226 C1ORF112 METTL13 PRDX6 RFWD2 CEP350 ACBD6 C1ORF25 C1ORF26 C1ORF27 TROVE2 C1ORF106 TMEM9 IPO9 KLHL12 SNRPE GOLT1A RBBP5 DSTYK PFKFB2 CD46 C1ORF74 RCOR3 INTS7 FLVCR1 RPS6KC1 GPATCH2 BPNT1 MOSC1 FBXO28 NVL PARP1 ZNF678 C1ORF35 NUP133 COG2 TTC13 PCNXL2 KIAA1804 C1ORF31 IRF2BP2 GGPS1 TBCE ZNF238
module 5	effector 1, intra segment CNV, chromosome 2<br>69 targets: RNASEH1 IAH1 ODC1 UBE2E3 MATN3 NRBP1 SUPT7L HNRPLL OLA1 SNRNP27 PCBP1 DUSP11 MOBKL1B HTRA2 C2ORF3 KCMF1 VPS24 ZAP70 NCK2 RANBP2 ANKRD57 DDX18 DBI PTPN4 TSN IWS1 DARS SPOPL MBD5 MMADHC ARL5A GALNT13 PKP4 TANK SSB METTL8 DCAF17 SLC25A12 HAT1 ITGA6 GPR155 ATP5G3 KIAA1715 AGPS PRKRA SSFA2 ZC3H15 ASNSD1 ORMDL1 NAB1 MYO1B SF3B1 C2ORF60 ALS2CR4 SUMO1 ABI2 EEF1B2 FASTKD2 XRCC5 DKFZp434H1419 CUL3 MFF AGFG1 CCL20 SPHKAP UBE2F TWIST2 STK25 ING5
module 6	effector 1, intra segment CNV, chromosome 3<br>36 targets: GRM7 EIF1B SETD2 COL7A1 RNF123 UBA3 ARL6IP5 HTR1F C3ORF38 CLDND1 TFG C3ORF17 PLA1A IQCB1 MBD4 TOPBP1 ANAPC13 MSL2 STAG1 DBR1 ARMC8 XRN1 SR140 HPS3 COMMD2 PDCD10 ZNF639 ATP11B DCUN1D1 DVL3 AP2M1 ST6GAL1 RTP4 LOC344887 FBXO45 PAK2
module 7	effector 1, intra segment CNV, chromosome 4<br>9 targets: GUF1 UGT2B4 RUFY3 MOBKL1A WDFY3 BDH2 ANK2 CAMK2D METTL14

(*continued*)

module 8	effector 1, intra segment CNV, chromosome 5<br>23 targets: MARVELD2 TRIP13 LPCAT1 MRPL36 CCT5 RNASEN C5ORF22 TARS RAD1 BRIX1 PRLR C5ORF33 NUP155 MRPS30 KIF3A SKP1 NDFIP1 RBM27 RBM22 DCTN4 LARP1 CCDC99 MGAT4B
module 9	effector 1, intra segment CNV, chromosome 6<br>70 targets: HCG18 BPHL VPS52 GCNT2 ZSCAN16 ZNF187 C6ORF134 CCHCR1 EHMT2 AGPAT1 PBX2 RING1 ZBTB22 CUTA ITPR3 C6ORF125 SNRPC TAF8 KLHDC3 PTK7 SLC35B2 LOC730101 MTO1 SENP6 DOPEY1 SYNCRIP ORC3L CASP8AP2 MAP3K7 FBXL4 USP45 PREP ATG5 AIM1 QRSL1 SEC63 OSTM1 SNX3 C6ORF182 WASF1 CDC40 AMD1 TUBE1 TSPYL1 RWDD1 ZUFSP GOPC NUS1 ASF1A RNF217 TRMT11 RSPO3 RNF146 TBPL1 HBS1L VTA1 PEX3 TAB2 PPIL4 KATNA1 C6ORF72 PCMT1 PLEKHG1 ZBTB2 FBXO5 TMEM181 ACAT2 TCP1 MRPL18 PHF10
module 10	effector 1, intra segment CNV, chromosome 8<br>43 targets: DEFA4 LONRF1 EFHA2 ZDHHC2 CNOT7 VPS37A PCM1 PPP3CC BNIP3L CCDC25 LEPROTL1 DCTN6 UBXN8 WRN MAK16 LSM1 BAG4 DDHD2 PLEKHA2 GOLGA7 FNTA CHCHD7 SNHG6 KCNB2 ZBTB10 CHMP4C CA3 COX6C LRP12 NUDCD1 SNTB1 SLC45A4 PTP4A3 NAPRT1 SCRIB PUF60 CYC1 HSF1 DGAT1 ADCK5 SLC39A4 ZNF250 C8ORF33
module 11	effector 1, intra segment CNV, chromosome 9<br>58 targets: CDC37L1 C9ORF46 CD274 PDCD1LG2 KIAA1432 KIAA2026 RANBP6 C9ORF123 PSIP1 CNTLN RRAGA HAUS6 RPS6 KLHL9 TUSC1 PLAA C9ORF72 TOPORS LOC100129250 APTX DNAJA1 SMU1 NFX1 UBE2R2 GLIPR2 TOMM5 FAM122A GNAQ AGTPBP1 SEMA4D SYK SPTLC1 PHF2 SEC61B STX17 TEX10 MRPL50 NIPSNAP3A SLC44A1 C9ORF6 GNG10 MRRF PDCL RC3H2 ZBTB6 ZBTB26 PPP6C MAPKAP1 ZBTB34 ENG SET IER5L EXOSC2 AIF1L RAPGEF1 SETX RALGDS UBAC1
module 12	effector 1, intra segment CNV, chromosome 10<br>5 targets: GPRIN2 SORBS1 C10ORF84 PLEKHA1 PPP2R2D
module 13	effector 1, intra segment CNV, chromosome 11<br>10 targets: ZNF195 NUP98 CYB5R2 SWAP70 CTR9 LIN7C EXT2 KBTBD4 NARS2 SNX19
module 14	effector 1, intra segment CNV, chromosome 12<br>11 targets: TNFRSF1A MRPL51 MLF2 PEX5 LOC100129361 PLBD1 DERA AEBP2 KLHDC5 RAB5B ANO4
module 15	effector 1, intra segment CNV, chromosome 13<br>8 targets: ZMYM5 NUPL1 GTF3A POLR1D USPL1 FAM48A MED4 TDRD3
module 16	effector 1, intra segment CNV, chromosome 14<br>21 targets: SUPT16H REM2 CDH24 PABPN1 KIAA0391 FANCM SOCS4 KIAA0586 GPHN PLEKHH1 RDH11 ZNF410 ENTPD5 POMT2 SNW1 SMEK1 GSC GLRX5 BDKRB1 CCDC85C MGC23270
module 17	effector 1, intra segment CNV, chromosome 15<br>12 targets: RPS17 NOP10 FAM98B INO80 LOC729082 RTF1 LOC645212 DUT C15ORF17 SEC11A ZNF592 LYSMD4
module 18	effector 1, intra segment CNV, chromosome 16<br>1 targets: AKTIP
module 19	effector 1, intra segment CNV, chromosome 17<br>26 targets: PITPNA C17ORF91 OR1E1 ASPA TMEM93 SPAG7 GPS2 EPN2 MFAP4 ULK2 NLK RUNDC1 PHB TUBD1 BCAS3 ICAM2 ABCA8 C17ORF80 SUMO2 GGA3 PRPSAP1 ASPSCR1 RAC3 GPS1 FASN CSNK1D
module 20	effector 1, intra segment CNV, chromosome 19<br>1 targets: BCAT2

(*continued*)

module 21	effector 1, intra segment CNV, chromosome 20<br>33 targets: TBC1D20 CSNK2A1 PSMF1 SNRPB ITPA RNF24 PCNA C20ORF196 TRMT6 CRLS1 SNRPB2 SNX5 C20ORF72 ZNF133 C20ORF12 POLR3F RBBP9 SEC23B DTD1 CRNKL1 NXT1 C20ORF3 GINS1 NANP TM9SF4 RALY DYNLRB1 UQCC CPNE1 C20ORF4 KIAA0406 RALGAPB STAU1
module 22	effector 1, intra segment CNV, chromosome 21<br>10 targets: ATP5J SOD1 SON HMGN1 MX2 MX1 TRAPPC10 PTTG1IP LOC642852 C21ORF57
module 23	effector 1, intra segment CNV, chromosome 22<br>4 targets: GSTT2 EWSR1 SMTN PACSIN2
module 24	effector 1, intra segment CNV, chromosome 23<br>12 targets: ZRSR2 ZFX ARMCX5 UPF3B ZBTB33 THOC2 XIAP STAG2 MBNL3 FAM122B BCAP31 G6PD
module 25	effector 1, inter segment CNV, chromosome 1, positive, 1 regulators: PRCC<br>77 targets: ANKRD39 LOC285103 TIMP4 CCRL2 CLSTN2 LIN54 DDIT4L AHRR TBCA SPINK1 PDGFRB ARSI GEMIN5 ODZ2 HIST1H2BD HIST1H2BI GUSBL1 CLDN3 TRIM73 TRIM24 PAXIP1 CLDN23 ESRP1 GRHL2 KCNQ3 IL33 IGFBPL1 ALDH1A1 BSPRY NDUFA8 CRB2 ZCCHC24 PRKCDBP CD59 TSPAN9 RERG HOTAIR HOXC11 ZBTB39 TMEM119 SH2B3 PSME1 C14ORF104 FRMD6 MLH3 C14ORF139 RPAP1 PLEKHO2 PRM3 ERAL1 DHRS11 PSMB3 GRB7 PSMD3 KRT33B NT5C3L PTRF TUBG1 VPS25 NAGS CCDC43 EFTUD2 PRR15L SLC35B1 EPN3 RNF43 DCXR PHLPP1 ZSWIM4 ZNF536 CABLES2 GGT5 ACE2 MAP7D2 PHF16 PHKA1 CXORF61
module 26	effector 1, inter segment CNV, chromosome 2, positive, 1 regulators: ODC1<br>81 targets: GLTPD1 SESN2 LCK NT5C1A ZMYND12 OLFML3 MTX1 SYT11 C1ORF66 SH2D2A C1ORF97 TAF1A RHOU PLXNB1 ROBO1 CNBP SERPINI1 LNX1 LOC153684 SIL1 ZNF354B SKIV2L FTSJD2 FGL2 ACHE TMEM213 C7ORF13 PKIA MAF1 GDI2 PRKCQ HNRNPF LOC255512 BSCL2 C11ORF73 MTMR2 ROBO3 HOXC13 ACSS3 CDK8 CAB39L COQ6 TMEM63C SERPINA10 ARPP19 GCNT3 LRRC49 MORF4L1 LOC283693 NGRN WASH3P WDR24 TBL3 E4F1 ABCA17P NMRAL1 UBN1 INO80E SETD1A COTL1 GUCY2D COASY LOC644246 GNA13 ARHGDIA ECH1 SLC8A2 OXT C20ORF94 INSM1 C20ORF134 BLCAP RAB36 UPK3A GTPBP6 CXORF59 WDR13 OPHN1 ZCCHC18 ZNF275 FAM3A
module 27	effector 1, inter segment CNV, chromosome 3, positive, 1 regulators: COL7A1<br>116 targets: TNFRSF18 PEX14 MIIP LYPLA2 PAQR7 XKR8 SESN2 ZMYND12 TMEM53 FAAH CPT2 GBP5 IFI16 TNFSF4 IER5 CAPN8 TRIB2 TMEM150A TFCP2L1 CYP27C1 NMI STAT1 PROM1 RPL9 PARM1 CXCL9 CXCL10 CXCL11 GRID2 PCDHB14 SPINK5 KIAA1949 TAP1 PSMB9 POR MDH2 PPP1R9A OR2F2 IDO1 EYA1 UHRF2 CTSL2 C9ORF30 UGCG EFCAB4A SAA4 PRDX5 C11ORF2 BRMS1 LOC441617 TSKU RPL13P5 C12ORF41 ACADS MORN3 KDELC1 JPH4 HIF1A ADAM21 BATF UBR7 DEGS2 WARS AKT1 FAM173A HS3ST6 CRYM SCNN1B NDUFAB1 IL21R PAPD5 MT3 CMTM4 CES8 ATP6V0D1 PSKH1 DUS2L ESRP2 FUK RILP PELP1 ACADVL LOC100128288 GUCY2D C17ORF76 PLD6 NEK8 SLFN11 CNTD1 PRR11 FN3K AFG3L2 ZNF532 GRIN3B TJP3 LASS4 CCDC151 OCEL1 ECH1 C19ORF54 BCKDHA B3GNT8 KLK11 ZNF304 ZNF134 WFDC3 SNX21 RPS21 TFF3 CECR5 AIFM3 MCAT KLHDC7B GTPBP6 XK PIM2
module 28	effector 1, inter segment CNV, chromosome 5, positive, 1 regulators: PRLR<br>87 targets: HES4 SLC25A33 MAD2L2 PPP1R8 STIL USP1 DEPDC1 MSH4 NTRK1 ARL8A C1ORF186 EXO1 BOLA3 CKAP2L CYP27A1 CACNA2D2 POLQ LAMP3 RPL34 DSP HIST1H2AL GLO1 TTK MPP6 ZPBP GTF2IRD1 NCAPG2 SFTPC C8ORF58 MELK SFTA1P BNIP3 LOC643650 SFTPA2 SFTPD SLC18A2 FAM24A OR51E1 KIF18A MDK PSMC3 AQP11 HTR3A CHEK1 PTPN6 PSMA6 SLC25A21 ERO1L PSMC6 WDHD1 DLST AHSA1 OIP5 CAPN3 FRMD5 PATL2 LOC283761 TPSB2 GINS3 RFWD3 TERF2IP TUBB3 PIK3R6 ALDH3A1 SPAG5 BRCA1 THOC4 WDR45L NDC80 RBBP8 LOC647946 S1PR4 UHRF1 DNMT1 ICAM3 MAST3 ZNF675 RHPN2 BCL2L12 UBE2S C20ORF194 GRAP2 POLA1 SLC9A7 KIF4A LOC100131434 PLXNB3

(*continued*)

module 29	effector 1, inter segment CNV, chromosome 6, positive, 2 regulators: PBX2 RSPO3<br>141 targets: EFHD2 XKR8 SESN2 CAP1 FLJ32224 EPHX4 BCAR3 FAM63A LCE2B ROBLD3 PMF1 KIAA0040 PHLDA3 SYT2 HHAT C1ORF97 LQK1 ZNF692 UBXN2A RND3 CYP20A1 HCLS1 BPESC1 MCCC1 TMEM150C TRAM1L1 IL15 TMEM184C FAM149A IRF1 PCDHB7 SPINK5 CSF1R THG1L STK10 RAB24 SCIN PRPS1L1 ZNF713 VPS37D CLIP2 TRIM73 ZC3HC1 FLJ40852 ATP6V0E2 GALNT11 C8ORF48 SH2D4A HMBOX1 LETM2 GAPDHL7 NACAP1 EIF3H ENPP2 PIP5KL1 ENDOG SLC2A6 RNF208 GATA3 DHTKD1 UBTD1 NPM3 HPS6 AS3MT CALHM2 AFAP1L2 ILK GAS2 SPI1 C11ORF48 FERMT3 ZDHHC24 MRGPRF TSKU SLC37A2 RPL13P5 RPS26 CACNB3 TARBP2 BLOC1S1 CCDC63 SH2B3 ERP29 RPLP0 TRIAP1 ACADS GTF2H3 PABPC3 NAA16 SUGT1 DZIP1 HAUS4 EFS HEATR5A C14ORF149 RAB15 GPR65 WARS ATP8B4 PLEKHO2 DENND4A LCTL THAP10 ZG16 TPSD1 IL32 IGSF6 SBK1 TEPP FUK NLRP1 GUCY2D UNC119 TLCD1 SLFN11 KAT2A VPS25 DLX3 SPATA20 SNORD104 LOC100130933 DCXR TUBB6 CABLES1 C18ORF45 MUC16 NKPD1 MAMSTR MYADM PROCR MAFB WFDC5 ZNF512B IL17RA SLC5A4 APOL1 MYH9 ARHGAP8 CD99 CXORF50B FMR1
module 30	effector 1, inter segment CNV, chromosome 7, positive, 1 regulators: RPA3<br>124 targets: HES5 GPR153 MMACHC TXNDC12 TMEM48 PARS2 C8A USP1 DEPDC1 SSX2IP GLMN GSTM5 BCL2L15 ANXA9 S100A4 RAG1AP1 CRABP2 PEAR1 FMO2 TDRD5 PRG4 SMYD3 CRIM1 LOC728819 LOC151534 ITIH3 MUSTN1 FAM107A NAALADL2 KCNMB3 PSMD2 MAGEF1 LSG1 LOC152217 SLIT2 UGT8 NEIL3 LMBRD2 PIK3R1 ABLIM3 NEU1 HIST1H2AE ZBTB9 MED20 C6ORF168 FAM86B1 PPP3CC FBXO16 GPR124 SGK3 ZFPM2 IGFBPL1 TSC1 ZNF25 CXCL12 ZCCHC24 C10ORF116 ITPRIP MDK MEN1 CRYAB ZW10 FOXRED1 NCAPD3 CLEC1A LRRK2 DDX23 INHBC TMTC4 ERO1L PLEK2 PSMC1 DLK1 FRMD5 RASL12 SENP8 ALPK3 C16ORF91 CLDN9 UBFD1 PALB2 DCTPP1 CHST6 C17ORF100 CHRNB1 CCT6B RDM1 TUBG1 TTLL6 SGCA ABCA6 FN3KRP CCDC102B DNMT1 RNASEH2A DAND5 MPV17L2 ZNF675 KLK11 CENPB ANKRD5 BFSP1 TTPAL WISP2 TH1L ADRM1 CABLES2 ZNF280B CHEK2 IL3RA RBBP7 SRPX HSD17B10 CSTF2 TRMT2B BEX2 ATG4A SLC25A5 HS6ST2 HTATSF1 LDOC1 CXORF40B LAGE3 SLC10A3
module 31	effector 1, inter segment CNV, chromosome 8, positive, 2 regulators: PCM1 NUDCD1<br>131 targets: CLSTN1 RNF186 ASAP3 SEPN1 LCK COL8A2 CAP1 IPO13 DNASE2B GNG5 CCBL2 GPR88 TMEM167B CTSK JTB MUC1 SLC25A44 TOMM40L RXRG ATP1B1 C1ORF220 CHIT1 KCNS3 XDH CNNM3 LOC440895 RPRM RAMP1 POU1F1 TMEM45A CHCHD6 TLR10 FAM175A ADH5 EGF LSM6 RNF175 NDUFS6 CCNO TBCA SMAD5OS FAF2 ZNF354B HMGN4 FKBPL MDFI COL10A1 PLAGL1 DFNA5 HSPB1 DTX2 OCM2 NPTX2 LAMB1 EN2 GLIS3 AK3 CER1 RASEF CDK20 TSTD2 STXBP1 AGPAT2 TPRN PRKCQ DNAJC12 PLA2G12B GOLGA7B HTRA1 AP2A2 SLC22A18 TRIM6 BBOX1 HARBI1 FKBP2 ATG2A YIF1A FXYD6 HYOU1 C3AR1 KLRG1 STK38L CCNT1 ACSS3 C12ORF43 LOC440131 CYSLTR2 VPS36 SP3P PARP2 RNASE6 CHD8 SRP54 KIAA0247 C14ORF4 WDR25 TMEM85 CCNDBP1 COPS2 RSL24D1 GCNT3 LOC283693 BTBD1 DEXI TMEM159 INO80E BCMO1 ANKRD13B DYNLL2 TCAM1P CBX4 ZADH2 ELANE PRAM1 STX10 LPHN1 FXYD5 DMRTC2 KCNN4 ACSS2 VSTM2L DOK5 C21ORF7 SDF2L1 TCN2 KIAA1661 LMF2 SHROOM2 CXORF59 ZNF630 PORCN
module 32	effector 1, inter segment CNV, chromosome 9, positive, 2 regulators: SYK PSIP1<br>184 targets: PUSL1 LRRC47 NOL9 C1ORF224 C1ORF134 ATP13A2 KLHDC7A UBXN10 LYPLA2 NR0B2 XKR8 SESN2 SERINC2 LCK KIAA1522 KIAA0319L TFAP2E DNALI1 ZMYND12 TMEM125 ELOVL1 TMEM53 CYP4Z1 OSBPL9 DHCR24 WLS CTH DNASE2B GBP4 GBP5 GFI1 CD2 CCDC19 SLAMF6 CD247 CD8A LIPT1 MITD1 LOC440895 DYNC1I2 MLPH LOC401052 UBE2E2 CXCR6 LRRC2 HYAL2 CISH TRAT1 C3ORF36 LRRC33 KCNIP4 CXCL9 CXCL10 ELOVL6 NPY1R CCDC111 SLC6A3 GZMK GZMA IRF1 PCDHB14 SPINK5 ITK COL23A1 DDAH2 SLC39A7 NRN1 HMGN4 PGBD1 DHX16 LYPLA2P1 KIAA1586 FAM26F THEMIS C6ORF94 AGR3 TARP C7ORF57 ASL WBSCR27 POR TMEM120A MDH2 LOC401387 PPP1R9A PON3 C7ORF68 GIMAP4 TMEM176A LOC100132891 PKIA RIPK2 OTUD6B C8ORF73 OPLAH PRKCQ CAMK1D OPTN SCT EFCAB4A SLC22A18 GVIN1 C11ORF49 MED19 TMX2 DDB1 BRMS1 BIRC3 CD3D TMEM45B GLB1L2 RPL13P5 CLEC2B NELL2 HOXC13 HSD17B6 SLC16A7 CHST11 NEK3 SP3P C13ORF39 GZMH GZMB FAM161B KIAA0284 NDNL2 COPS2 BNIP2 CTSH TBL3 NPW IL32 ALG1 BFAR CRYM COG7 CHP2 IL21R TUFM CDIPT MT3 POLR2C SETD6 GOT2 CMTM4 CES2 CES3 ATP6V0D1 AARS DHODH GPR172B PLSCR3 GUCY2D CCL5 TMEM99 FOXJ1 SMAD7 PPAP2C TJP3 LASS4 YIPF2 CCDC151 EPHX3 ATP13A1 ECH1 CNFN SEPW1 NKG7 CST7 C20ORF134 MMP24 WFDC3 CECR5 YDJC RAB36 MN1 TST GCAT GRAP2 MCAT UPK3A PRKX OTC IL2RG

(*continued*)

module 33	effector 1, inter segment CNV, chromosome 11, positive, 2 regulators: NUP98 EXT2<br>62 targets: FAM132A CTNNBIP1 KNCN HIAT1 EXTL2 MRPS21 DPM3 ROBLD3 CASQ1 PCP4L1 ACBD6 GNPAT UCN PLXNB1 ATP5I FSTL5 LOC100131067 ANKRD43 FAM153A C6ORF124 TMEM217 C6ORF130 TDRD6 IGF2R ARF5 RPS20 LOC643763 VPS28 RANBP6 CCDC107 HINT2 GCNT1 RXRA EDF1 ZDHHC6 KRT83 APPL2 RAN ATL1 PNMA1 IGHV7-81 NCRNA00052 AXIN1 LOC643714 MLKL C17ORF49 NEURL4 LSMD1 RAB5C DSG2 LIPG RAX RLN3 LPAR2 ZNF101 FKRP ROMO1 LRP5L WWC3 KLHL13 RBMX2 FAM50A
module 34	effector 1, inter segment CNV, chromosome 14, positive, 1 regulators: GPHN<br>222 targets: HES5 PHF13 FBXO6 NECAP2 PINK1 C1QA C1QC C1QB RPL11 CD52 STX12 ERMAP RAD54L ORC1L CDC7 FNDC7 C1ORF162 PHGDH FCGR1B MTMR11 TNFAIP8L2 HCN3 MNDA FCER1G CREG1 RCSD1 GORAB CFH NEK7 PHLDA3 RNPEP TMEM183A MOSC2 NTSR2 YPEL5 QPCT ACYP2 VAMP8 NEURL3 MFSD9 C2ORF40 CCDC115 ARHGAP15 CD302 TMBIM1 HES6 NKIRAS1 SACM1L BTLA CSTA CPA3 C3ORF43 PSAPL1 LAP3 PLAC8 ABCG2 ATOH1 UFSP2 C7 CETN3 SNX2 PCDHB11 MXD3 NRM C6ORF27 C2 PPT2 HLA-DPB1 LY86 HLA-DPA1 RSPH9 C6ORF223 SUPT3H RPS12 BEND3 CCDC28A CITED2 CCDC146 C7ORF23 TMEM130 ARPC1B PILRA C7ORF60 TFEC ZNF800 TPK1 LOC285972 GIMAP7 CLU SGK3 PGCP RPL30 RRM2B CA9 TMEM2 ALDH1A1 FBP1 SLC31A2 RNF183 GGTA1 PAEP TUBB2C WDR85 ZNF25 SGMS1 A1CF DNAJC12 ARHGAP19 CUTC BLOC1S2 PDZD7 C10ORF32 CALHM2 FANK1 LOC387723 C10ORF125 FTH1 C11ORF46 SYT13 MYBPC3 MPEG1 MS4A4A SLC15A3 C11ORF84 MTL5 SPCS2 UVRAG AQP11 ANKRD42 C11ORF75 CASP1 IL18 CRTAM PTPN6 CLEC4A KLRG1 KLRB1 CD69 OLR1 TAS2R9 CREBL2 HEBP1 ST8SIA1 TM7SF3 LRRK2 DNAJC22 GALNT6 NCKAP1L TMBIM4 SLC35E3 THAP2 NTN4 ISCU ALDH2 LOC440117 SLC46A3 KBTBD7 ATP7B NDUFAF1 LYSMD2 CCPG1 RNF111 WHAMM TM6SF1 IGSF6 ITGAL BBS2 CCDC113 DPEP2 DYNLRB2 ZDHHC7 IRF8 TUBB3 ITGAE CXCL16 TEKT1 CLEC10A TNFSF12 PIK3R6 ZNF624 SLC47A1 ALDOC CRLF3 EVI2A CCL18 C17ORF96 MPP2 CD300LF GAA SLC25A10 WDR45L FTL VAV1 FLJ22184 CD320 ZNF763 HAUS8 IFI30 TYROBP BLVRB NTF4 CD37 OSCAR SIGLEC1 LOC149837 MGC44328 HCK MORC3 ATP6V1E1 SLC5A1 CACNG2 CSF2RB TSPO PARVG RAI2 ELK1 VSIG4 BTK LUZP4 FAM70A SASH3 ARHGEF6
module 35	effector 1, inter segment CNV, chromosome 17, positive, 1 regulators: PHB<br>33 targets: SCP2 GBP2 SLC19A2 HIST3H2A LOC339535 TREX1 ST3GAL6 TTC14 LOC285547 LOC152742 TRIM61 IQGAP2 HCG26 GTF2IRD1 BET1 ACTL7B RNF183 TNC CD59 FRMD6 LOC146336 VASN ZNF516 C19ORF21 ARID3A CEBPA ZNF404 FCGRT BLCAP MC3R PRAME GGT5 NDP
module 36	effector 1, inter segment CNV, chromosome 20, positive, 1 regulators: RBBP9<br>23 targets: GLMN TMEM79 NCRNA00116 ZNF619 SHISA5 GCM2 HIST1H2AE HIST1H2BG IP6K3 OSR2 HSD17B7P2 OBFC1 NTF3 PLCZ1 KNTC1 ARL6IP1 ACSM2A HPR CCDC55 SLC25A41 MBD3L2 KLK11 SCML2
module 37	effector 1, inter segment CNV, chromosome 21, positive, 1 regulators: SOD1<br>24 targets: CDA TMEM222 TMEM115 HRH2 GUSBL1 HIST1H3I LOC154872 ZNF572 LOC642361 MANSC1 SCAMP2 CIB1 TSNAXIP1 PIGN LOC147727 ABHD8 COMP ZNF382 NR1H2 C19ORF73 OSBPL2 TPD52L2 RFPL1S SELO
module 38	effector 1, inter segment CNV, chromosome 22, positive, 1 regulators: EWSR1<br>49 targets: TTLL7 OST4 MTIF2 BIN1 TMEM194B ASB1 PLSCR2 TTC14 FGFBP2 CSN1S2A ANKRA2 PCDHB2 PCDHB7 GNPDA1 LOC100268168 HCP5 HIST1H2BE HIST1H2BK SLC22A2 RADIL NPVF SEMA3E BET1 TMEM213 MYBL1 PKN3 ILK TRMT112 DDX25 COPS7A SUOX PCK2 CBLN3 ACOT4 DAPK2 LRRC49 C15ORF51 TMEM88 MED24 METRNL CEBPA PEG3 C20ORF96 PKIG H2BFS CXORF23 CFP ACRC BEX1
module 39	effector 1, inter segment CNV, chromosome 23, positive, 1 regulators: ZFX<br>12 targets: ZSWIM5 FNBP1L GRB14 CACNA2D1 C8ORF40 C12ORF26 ETFA LOC100130894 VMO1 CNN2 ZNF486 STMN3

(continued)

module 40	effector 1, methylation ELAVL4 WDR77 REG4 RNASEL NR5A2 PLCL1 PROM1 ADH7 PALLD CASP3 AGR3 ABCB5 GHRHR ABCB1 TG IFNB1 TAL2 PTPN3 AKNA RAG2 TYR KLRK1 KCNRG CCL5 VSIG1, negative,<br>47 targets: C1ORF109 ELAVL4 WDR77 REG4 TOMM40L RNASEL NR5A2 USH2A PODXL2 HAUS3 PROM1 ADH7 PALLD CASP3 HMHB1 ASCC3 LIN28B GTF3C6 FRK AGR3 ABCB5 GHRHR ZNF138 ABCB1 NPTX2 TG IFNB1 TAL2 PTPN3 AKNA RAG2 TCN1 PC TYR KLRK1 KRT6A KCNRG LOC121952 SLC39A2 GNPNAT1 GAN CCL5 ZNF560 KCNJ14 SRXN1 SYAP1 VSIG1
module 41	effector 1, methylation S100A14 PVRL4 SIX2 GALNT3 IL12B AGR2 EGFR STEAP4 STEAP2 FGF20 PNMA2 BLNK FGFR2 CD82 MMP7 ERBB3 NCOR2 NR2F2 CTF1 TM4SF5 TNS4 FXYD3 FERMT1 BCAS1 BMP7 ZBP1 PRAME, negative,<br>48 targets: FLJ40434 S100A14 PVRL4 PIK3C2B C4BPA COLEC11 SIX2 GALNT3 IL12B AGR2 EGFR MAGI2 STEAP4 STEAP2 FGF20 PNMA2 C8ORF71 C9ORF152 TSPAN15 BLNK FGFR2 CD82 LRP4 CDC42EP2 CCDC83 MMP7 RPL6 ERBB3 COQ10A NCOR2 MOAP1 CDC42BPB DNM1P41 NR2F2 LYRM1 CTF1 PDP2 TM4SF5 TNS4 HEXIM2 LOC113230 FXYD3 FERMT1 BCAS1 ZBP1 PRAME TRMU TFDP3
module 42	effector 1, methylation ATF6 BARD1 BCL6 EBF1 MICB HFE GLI3 RECK LZTS2 C11ORF30 VDR RGS6 NUMB NRG4 TP53 STAT3 NME1 C19ORF6 MLLT1 AKT2 FOSB SOD1, negative,<br>31 targets: ATF6 SCN9A BARD1 GRK7 BCL6 C4ORF6 EBF1 HFE FKBP9 GLI3 PLOD3 RECK LZTS2 FADS1 C11ORF30 RGS6 NUMB NRG4 TP53 STAT3 NME1 FLJ36644 KIR2DS2 C19ORF6 MLLT1 AKT2 FOSB KIR3DL3 KIR2DL3 TMC2 SOD1
module 43	effector 1, methylation GFI1 TMEFF2 IHH GATA2 CXCL1 POU4F2 SPARC GCM2 FSCN1 MEOX2 HOXA5 HOXA9 PTN NKX6-2 MYOD1 FLI1 ACVRL1 HOXC11 POU4F1 HOXB4 MAFB SIM2, negative,<br>22 targets: GFI1 TMEFF2 IHH GATA2 CXCL1 POU4F2 SPARC GCM2 FSCN1 MEOX2 HOXA5 HOXA9 PTN NKX6-2 MYOD1 FLI1 ACVRL1 HOXC11 POU4F1 HOXB4 MAFB SIM2
module 44	effector 1, methylation LCK FCGR2B NR3C1 LGI1 LAG3 CD69 DCD GLIPR1 CST7, negative,<br>40 targets: LCK ZNF642 FCGR2B KIF21B DTL SLC16A14 HAUS3 MAD2L1 MND1 NR3C1 FUCA2 NFE2L3 ZNRF2 MGC16075 DBF4 NPTX2 RINT1 EZH2 PBK DECR1 PDP1 CEP55 LGI1 PRMT3 TMEM123 LAG3 CD69 GLIPR1 SHCBP1 GAN C17ORF58 ABHD3 CST7 C21ORF45 PCP4 PRPS2 GLA SLC25A43 UBE2A RAP2C

Supplementary Table S2. Results of validation tests. A summary of the test statistics calculated for each of the validation tests: (a) The adjusted Kolmogorov-Smirnov *p*-values for the validation of passenger coherence in female external East Asian subdata; (b) The adjusted Kolmogorov-Smirnov *p*-values for the validation of prognostic power in female external East Asian subdata; and (c) A summary of log-rank *p*-values for the Kaplan-Meier curves of female external East Asian subdata.

(a) Expression coherence of target genes (Female East Asian). Kolmogorov-Smirnov adjusted *p*- values for 2-sided test.

**Module**	**1**	**2**	**3**	**4**	**5**	**6**	**7**	**8**	**9**	**10**
Japan_F	0.001	0.001	0.001	0.001	0.001	0.001	0.001	0.001	0.001	0.001
Korea_F	0.001	0.001	0.001	0.001	0.001	0.001	0.14	0.001	0.001	0.007
Module	11	12	13	14	15	16	17	18	19	20
Japan_F	0.001	0.013	0.001	0.001	0.001	0.001	0.001	1	0.001	1
Korea_F	0.001	0.662	0.001	0.38	0.001	0.001	0.001	1	0.018	1
Module	21	22	23	24	25	26	27	28	29	30
Japan_F	0.001	0.001	0.465	0.001	0.001	0.001	0.001	0.001	0.001	0.001
Korea_F	0.001	0.556	0.023	0.001	0.001	0.001	0.001	0.001	0.001	0.001
Module	31	32	33	34	35	36	37	38	39	40
Japan_F	0.001	0.001	0.025	0.001	0.716	0.819	0.001	0.072	0.604	0.11
Korea_F	0.001	0.001	0.001	0.001	0.001	0.086	0.004	0.001	0.327	0.001
Module	41	42	43	44						
Japan_F	0.12	0.13	0.001	0.001						
Korea_F	0.001	0.098	0.001	0.001						

(*continued*)

Kolmogorov-Smirnov adjusted *p*-values for 1-sided test.

**Module**	**1**	**2**	**3**	**4**	**5**	**6**	**7**	**8**	**9**	**10**
Japan_F	0.001	0.001	0.001	0.001	0.001	0.001	0.001	0.001	0.001	0.001
Korea_F	0.001	0.048	0.001	0.001	0.001	0.001	0.071	0.001	0.001	0.088
Module	11	12	13	14	15	16	17	18	19	20
Japan_F	0.001	0.001	0.001	0.001	0.001	0.001	0.001	1	0.001	1
Korea_F	0.001	0.422	0.001	0.346	0.001	0.001	0.001	1	0.091	1
Module	21	22	23	24	25	26	27	28	29	30
Japan_F	0.001	0.001	0.239	0.001	0.001	0.001	0.001	0.001	0.001	0.001
Korea_F	0.001	0.282	0.013	0.001	0.881	1	1	0.001	1	1
Module	31	32	33	34	35	36	37	38	39	40
Japan_F	0.001	0.001	0.004	0.001	0.686	0.437	0.001	0.038	0.474	0.065
Korea_F	0.076	0.999	0.966	0.001	0.056	0.043	0.086	0.011	0.992	0.001
Module	41	42	43	44						
Japan_F	0.072	0.121	0.001	0.001						
Korea_F	0.004	0.843	0.001	0.001						

(*continued*)

(b) Prognostic power of association modules (Female East Asian). Kolmogorov-Smirnov *p*-values for 2-sided test (detect any significant difference from background of random genes).

**Module**	**1**	**2**	**3**	**4**	**5**	**6**	**7**	**8**	**9**	**10**
Japan_F	0.001	0.001	0.001	0.001	0.086	0.001	0.03	0.007	0.814	0.003
Korea_F	0.001	0.001	0.001	0.001	0.001	0.001	0.002	0.001	0.001	0.179
Module	11	12	13	14	15	16	17	18	19	20
Japan_F	0.6	0.092	0.079	0.085	0.234	0.002	0.047	1	0.215	1
Korea_F	0.002	0.572	0.045	0.321	0.02	0.572	0.045	1	0.17	1
Module	21	22	23	24	25	26	27	28	29	30
Japan_F	0.001	0.05	0.923	0.728	0.006	0.852	0.636	0.001	0.177	0.001
Korea_F	0.001	0.006	0.607	0.685	0.007	0.731	0.02	0.001	0.015	0.001
Module	31	32	33	34	35	36	37	38	39	40
Japan_F	0.894	0.19	0.518	0.001	0.518	0.085	0.943	0.594	0.576	0.261
Korea_F	0.001	0.006	0.243	0.001	0.497	0.744	0.432	0.377	0.365	0.205
Module	41	42	43	44						
Japan_F	0.047	0.842	0.509	0.004						
Korea_F	0.293	0.555	0.306	0.001						

(*continued*)

Kolmogorov-Smirnov *p*-values for 1-sided test (greater than).

**Module**	**1**	**2**	**3**	**4**	**5**	**6**	**7**	**8**	**9**	**10**
Japan_F	0.001	0.001	0.001	0.001	0.051	0.001	0.975	0.006	0.422	0.002
Korea_F	0.001	0.001	0.001	0.001	0.001	0.001	0.975	0.001	0.001	0.989
Module	11	12	13	14	15	16	17	18	19	20
Japan_F	0.409	0.762	0.039	0.047	0.139	0.002	0.908	1	0.001	1
Korea_F	0.002	0.344	0.021	0.9	0.946	0.032	0.043	1	0.001	1
Module	21	22	23	24	25	26	27	28	29	30
Japan_F	0.1	0.027	0.735	0.382	0.006	0.584	0.344	0.001	0.983	0.001
Korea_F	0.001	0.002	0.523	0.823	0.003	0.577	0.01	0.001	0.006	0.001
Module	31	32	33	34	35	36	37	38	39	40
Japan_F	0.529	0.833	0.239	0.937	0.951	0.037	0.677	0.993	0.295	0.134
Korea_F	0.001	0.003	0.126	0.001	0.241	0.417	0.225	0.911	0.17	0.111
Module	41	42	43	44						
Japan_F	0.752	0.473	0.951	0.001						
Korea_F	0.165	0.289	0.822	0.001						

(*continued*)

Kolmogorov-Smirnov *p*-values for 1-sided test (less than).

**Module**	**1**	**2**	**3**	**4**	**5**	**6**	**7**	**8**	**9**	**10**
Japan_F	0.979	0.882	0.997	0.989	0.894	0.965	0.017	0.996	0.783	0.965
Korea_F	1	0.984	0.992	0.999	0.999	1	0.002	0.999	0.876	0.085
Module	11	12	13	14	15	16	17	18	19	20
Japan_F	0.303	0.053	0.727	0.993	0.131	0.999	0.022	1	0.712	1
Korea_F	0.39	0.307	0.288	0.162	0.015	0.389	0.965	1	0.602	1
Module	21	22	23	24	25	26	27	28	29	30
Japan_F	0.501	0.941	0.528	0.476	0.946	0.499	0.605	0.829	0.084	0.932
Korea_F	0.988	0.943	0.308	0.351	0.98	0.373	0.977	0.945	0.91	0.89
Module	31	32	33	34	35	36	37	38	39	40
Japan_F	0.508	0.092	0.638	0.001	0.248	0.855	0.598	0.309	0.962	0.694
Korea_F	0.916	0.509	0.961	0.111	0.435	0.482	0.735	0.198	0.588	0.655
Module	41	42	43	44						
Japan_F	0.025	0.748	0.239	0.993						
Korea_F	0.759	0.586	0.163	0.997						

(*continued*)

(c) Kaplan-Meier Log-rank *p*-values (Female East Asian). Log-rank test *p*-values from Kaplan-Meier curves

**Module**	**1**	**2**	**3**	**4**	**5**	**6**	**7**	**8**	**9**	**10**
Japan_F	0.091	0.051	0.038	0.145	0.354	0.174	0.21	0.118	0.282	0.443
Korea_F	0.001	0.01	0.007	0.059	0.142	0.447	0.46	0.034	0.568	0.918
Module	11	12	13	14	15	16	17	18	19	20
Japan_F	0.698	1	0.192	0.571	0.924	0.165	0.588	1	0.83	1
Korea_F	0.768	0.759	0.581	0.709	0.252	0.47	0.603	1	0.196	1
Module	21	22	23	24	25	26	27	28	29	30
Japan_F	0.77	0.129	0.748	0.821	0.292	0.333	0.852	0.122	0.626	0.009
Korea_F	0.107	0.963	0.852	0.232	0.113	0.113	0.967	0.021	0.819	0.136
Module	31	32	33	34	35	36	37	38	39	40
Japan_F	0.42	0.897	0.999	0.503	0.025	0.075	0.786	0.499	0.674	0.522
Korea_F	0.404	0.681	0.285	0.335	0.138	0.811	0.769	0.199	0.659	0.358
Module	41	42	43	44						
Japan_F	0.838	0.769	0.41	0.103						
Korea_F	0.327	0.713	0.038	0.215						

(*continued*)

Supplementary Table S3. Pubmed co-citation analysis and OMIM intersection of cancer-related genes. To find candidate effector genes on inferred association modules, we employed two tests. (a) The first examined whether module members were frequently co-cited with variants of the keyword ”lung cancer” from prior studies. We wrote a Pearl script to query the NCBI PubMed database and counted the number of publications where variants of the keyword ”lung cancer” and the name of a gene were co-present in the text. We then sorted all the genes according to their co-citation numbers and identified the intersection of the top-ranking 5% genes and members in each module. (b) The second approach utilizes the NCBI OMIM database of cancer-related genes. The table presents the intersection of listed cancer-related genes in the OMIM database with target genes of the significant association modules.

(a) PUBMED Co-citation analysis of selected genes and variants of the keyword “lung cancer”.

Symbol	Module	PubMed Search
EGFR	1	5886
RAC1	1	143
EZH2	3	63
FOXM1	3	47
TK1	3	25
RALBP1	2	23
THY1	3	22
RRM2	3	18
CDC20	3	16
CDK5	1	15

*High citation counts were found for BASE, BLM and NCL genes. However, as these terms have generic meanings beyond a gene label, the citations were further investigated and found not to have sufficient links to lung cancer to achieve significance.

(*continued*)

(b) Intersection of module members with NCBI OMIM database of cancer-related genes.

Module 1	Module 2	Module 3
FTSJ2		UBIAD1
NUDT1		CDC20
RAC1		KIF14
KDELR2		RRM2
ICA1		TP5313
CBX3		NCL
STARD3NL		NCAPG
PPIA		CCNA2
EGFR		KIF20A
RFC2		PTTG1
CASP2		HMMR
ARHGEF2		BYSL
ZNF282		EXH2
		PBK
		IFNA1
		CEP55
		MKI67
		THY1
		TROAP
		ESPL1
		SLC39A2
		BLM
		TK1
		MYBL2
		CENPM
		PAGE4

(*continued*)

Supplementary Table S4. IPA pathway analysis. A pathway enrichment was performed on the target genes of all modules using QIAGENs Ingenuity pathway analysis (IPA). The IPA software provides matches of functions or pathways with a significant overlap with the target genes. For each pathway, a *p*-value is calculated using a right tailed Fishers exact test to assess the likelihood that any identified overlap of genes is statistically significant (i.e. not due to random chance). As the software performs tests on a large number of pathways, it is appropriate to adjust for multiple testing and provides an adjusted *p*-value using the Benjamini-Hochberg method. Significance was determined by $p < 0.01$. The results were further filtered to report only pathways with an intersection > 4 genes.

**Module 1**	metabolism of DNA (p = 3.50E-04)<br>molecules: CASP2, EGFR, HUS1, POLD2, POLM, PPIA, RAC1, RPA3<br><br>DNA replication (p = 5.60E-04)<br>molecules: EGFR, HUS1, POLD2, POLM, RAC1, RPA3<br><br>repair of DNA (p = 9.96E-04)<br>molecules: EGFR, FIGNL1, HUS1, POLM, RAC1, RPA3<br><br>cell death of pheochromocytoma cell lines (p = 1.04E-03)<br>molecules: CASP2, EGFR, PPIA, RAC1<br><br>development of cytoplasm (p = 3.58E-03)<br>molecules: ATG9B, CDK5, CHCHD2, EGFR, FIS1, RAC1, SLC4A2<br><br>translation of protein (p = 3.89E-03)<br>molecules: EGFR, EIF2AK1, EIF3B, FTSJ2, PURB<br><br>translation of mRNA (p = 5.13E-03)<br>molecules: EGFR, EIF2AK1, EIF3B, PURB<br><br>morphogenesis of neurites (p = 7.45E-03)<br>molecules: CDK5, DBNL, EGFR, RAC1, SP4, TMEM106B
**Module 2**	NA
	uterine serous papillary cancer (p = 3.82E-13)<br>molecules: CCNA2, CDC20, ESPL1, FOXM1, HMMR, KIF11, KIF2C, KPNA2, MYBL2, PAGE4, PTTG1, SPC25, TK1, TPX2, UBE2C<br><br>segregation of chromosomes (p = 2.17E-11)<br>molecules: CCNA2, ESPL1, HJURP, KIF11, KIF2C, NCAPG, NUF2, PTTG1, SPC25<br><br>alignment of chromosomes (p = 1.80E-08)<br>molecules: CCNA2, DLGAP5, KIF14, KIF2C, NCAPG<br><br>mitosis of tumor cell lines (p = 9.38E-08)<br>molecules: CDC20, DLGAP5, FOXM1, KIF11, NUF2, PTTG1, SPC25, TPX2<br><br>mitosis of cervical cancer cell lines (p = 1.93E-07)<br>molecules: CDC20, DLGAP5, KIF11, NUF2, PTTG1, SPC25, TPX2<br><br>cell cycle progression (p = 6.78E-07)<br>molecules: BLM, CCNA2, CDC20, CDKN3, DLGAP5, EZH2, FOXM1, KIF11, KIF2C, NUF2, PTTG1, RNF4, SPC25, TPX2, UBE2C

(*continued*)

**Module 3**	M phase (*p* = 7.06E-07)<br>molecules: CDC20, CEP55, DLGAP5, KIF14, KIF20A, NUF2, PTTG1, UBE2C<br><br>mitosis (*p* = 1.49E-06)<br>molecules: CDC20, DLGAP5, FOXM1, KIF11, KIF2C, NUF2, PTTG1, SPC25, TPX2, UBE2C<br><br>apoptosis (*p* = 1.87E-06)<br>molecules: BLM, CDC20, CDCA2, CDX1, E2F6, ESPL1, EZH2, HMMR, IFNA1/IFNA13, KIF11, KIF14, KPNA2, LMNB1, MYBL2, NCL, NUF2, PBK, PTTG1, RRM2, SPC25, TAF4B, THY1, TPX2, ZC3H8<br><br>cell death (*p* = 6.25E-06)<br>molecules: BLM, CDC20, CDCA2, CDX1, E2F6, ESPL1, EZH2, FOXM1, HMMR, IFNA1/IFNA13, KIF11, KIF14, KPNA2, LMNB1, MYBL2, NCL, NUF2, PBK, PTTG1, RRM2, SPC25, TAF4B, THY1, TK1, TPX2, UBE2C, ZC3H8<br><br>necrosis (*p* = 8.45E-06)<br>molecules: BLM, CDC20, CDCA2, CDX1, E2F6, EZH2, HMMR, IFNA1/IFNA13, KIF11, KIF14, KPNA2, LMNB1, MYBL2, NCL, NUF2, PBK, PTTG1, RRM2, SPC25, THY1, TK1, TPX2, UBE2C, ZC3H8<br><br>cell death of tumor cell lines (*p* = 1.14E-05)<br>molecules: BLM, CDC20, CDCA2, CDX1, EZH2, HMMR, KIF11, KIF14, KPNA2, LMNB1, MYBL2, NCL, NUF2, PBK, PTTG1, RRM2, SPC25, THY1, TK1, TPX2, UBE2C<br><br>apoptosis of tumor cell lines (*p* = 1.39E-05)<br>molecules: BLM, CDC20, CDCA2, CDX1, EZH2, HMMR, KIF11, KIF14, KPNA2, LMNB1, NCL, NUF2, PBK, PTTG1, RRM2, SPC25, THY1, TPX2<br><br>apoptosis of cervical cancer cell lines (*p* = 4.96E-05)<br>molecules: CDC20, CDCA2, KIF11, KIF14, NUF2, PBK, SPC25, TPX2<br><br>gastrointestinal tract cancer (*p* = 5.16E-05)<br>molecules: BLM, BYSL, CCNA2, CDKN3, CDX1, CDYL2, CEP55, CST1, DLGAP5, E2F6, EZH2, FASTKD1, FOXM1, GLRA2, GYLTL1B, HMMR, IFNA1/IFNA13, IL1RAPL2, KIAA1210, KIF14, KIF18B, KIF20A, KPNA2, MAGEL2, MKI67, NCAPG, NCL, NIPA2, NUF2, PBK, PRB4, PTH2R, PTTG1, RRM2, SPC25, TET1, THY1, TK1, UBE2C<br><br>M phase of tumor cell lines (*p* = 5.80E-05)<br>molecules: CEP55, KIF14, KIF20A, NUF2, PTTG1<br><br>proliferation of tumor cell lines (*p* = 7.26E-05)<br>molecules: CCNA2, CDCA2, CDX1, DLGAP5, EZH2, FOXM1, GYLTL1B, HMMR, KIF20A, KPNA2, MKI67, NCAPG, NCL, PBK, PTTG1, RRM2, TPX2, UBE2C, UBIAD1<br><br>digestive tract cancer (*p* = 1.40E-04)<br>molecules: BLM, BYSL, CCNA2, CDKN3, CDX1, CDYL2, CEP55, CST1, DLGAP5, E2F6, EZH2, FASTKD1, FOXM1, GLRA2, GYLTL1B, HIST1H3B, HMMR, IFNA1/IFNA13, IL1RAPL2, KIAA1210, KIF14, KIF18B, KIF20A, KPNA2, MAGEL2, MKI67, NCAPG, NCL, NIPA2, NUF2, PBK, PRB4, PTH2R, PTTG1, RRM2, SPC25, TET1, THY1, TK1, TPX2, UBE2C<br><br>digestive organ tumor (*p* = 1.61E-04)<br>molecules: BLM, BYSL, CCNA2, CDKN3, CDX1, CDYL2, CEP55, CST1, DLGAP5, E2F6, EZH2, FASTKD1, FOXM1, GLRA2, GYLTL1B, HIST1H3B, HMMR, IFNA1/IFNA13, IL1RAPL2, KIAA1210, KIF14, KIF18B, KIF20A, KPNA2, MAGEL2, MKI67, NCAPG, NCL, NIPA2, NUF2, PBK, PRB4, PTH2R, PTTG1, RRM2, SPC25, TET1, THY1, TK1, TPX2, UBE2C

(*continued*)

	hepatocellular carcinoma (*p* = 1.90E-04)<br>molecules: CCNA2, CDKN3, EZH2, HIST1H3B, NCAPG, RRM2, TPX2, UBE2C<br><br>G2 phase (*p* = 2.26E-04)<br>molecules: BLM, CCNA2, FOXM1, KIF11, KPNA2, MYBL2<br><br>proliferation of cells (*p* = 2.33E-04)<br>molecules: BLM, CCNA2, CDCA2, CDKN3, CDX1, DLGAP5, EZH2, FOXM1, GYLTL1B, HMMR, KIF11, KIF20A, KIF2C, KPNA2, LMNB1, MKI67, MYBL2, NCAPG, NCL, PBK, PRSS2, PTTG1, RRM2, TPX2, UBE2C, UBIAD1<br><br>colorectal cancer (*p* = 2.80E-04)<br>molecules: BLM, BYSL, CCNA2, CDKN3, CDYL2, CEP55, CST1, DLGAP5, E2F6, EZH2, FASTKD1, GYLTL1B, HMMR, IFNA1/IFNA13, IL1RAPL2, KIAA1210, KIF14, KIF18B, KIF20A, KPNA2, MAGEL2, MKI67, NCAPG, NCL, NIPA2, NUF2, PBK, PRB4, PTH2R, PTTG1, RRM2, SPC25, TET1, TK1, UBE2C<br><br>arrest in G2 phase (*p* = 2.85E-04)<br>molecules: BLM, CCNA2, FOXM1, KIF11, MYBL2<br><br>metastasis (*p* = 3.28E-04)<br>molecules: DLGAP5, EZH2, FOXM1, IFNA1/IFNA13, KIF20A, NCAPG, PBK, PTTG1, RRM2<br><br>acute myeloid leukemia (*p* = 4.20E-04)<br>molecules: EZH2, FOXM1, PRSS2, RRM2, TET1, UBE2C<br><br>glioblastoma cancer (*p* = 4.71E-04)<br>molecules: CDKN3, EZH2, MKI67, PTTG1, RRM2, UBE2C<br><br>interphase (*p* = 5.42E-04)<br>molecules: BLM, CCNA2, CDCA2, CDKN3, EZH2, FOXM1, KIF11, KPNA2, MYBL2<br><br>neuroepithelial tumor (*p* = 5.49E-04)<br>molecules: CDKN3, EZH2, HIST1H3B, KIF14, KPNA2, MKI67, PTTG1, RRM2, UBE2C<br><br>colon cancer (*p* = 6.90E-04)<br>molecules: BLM, BYSL, CCNA2, CDKN3, CDYL2, CEP55, CST1, DLGAP5, EZH2, FASTKD1, GYLTL1B, HMMR, IFNA1/IFNA13, KIAA1210, KIF14, KIF18B, KPNA2, MAGEL2, MKI67, NCAPG, NCL, NIPA2, NUF2, PBK, PRB4, PTH2R, PTTG1, RRM2, SPC25, TET1, TK1<br><br>respiratory system tumor (*p* = 7.87E-04)<br>molecules: CCNA2, CDC20, CDCA2, EZH2, FOXM1, HIST1H3B, MKI67, PRSS2, RRM2, THY1, TK1, UBE2C, UBE2NL<br><br>breast or colorectal cancer (*p* = 8.56E-04)<br>molecules: BLM, BYSL, CCNA2, CDC20, CDKN3, CDYL2, CEP55, CST1, DLGAP5, E2F6, EZH2, FASTKD1, FOXM1, GYLTL1B, HMMR, IFNA1/IFNA13, IL1RAPL2, KIAA1210, KIF14, KIF18B, KIF20A, KPNA2, MAGEL2, MKI67, MYBL2, NCAPG, NCL, NIPA2, NUF2, PBK, PRB4, PTH2R, PTTG1, RRM2, SPC25, TET1, TK1, UBE2C<br><br>arrest in interphase of tumor cell lines (*p* = 9.42E-04)<br>molecules: CCNA2, CDCA2, EZH2, FOXM1, KIF11, MYBL2

(*continued*)

	abdominal cancer (*p* = 1.06E-03)<br>molecules: BLM, BYSL, CCNA2, CDC20, CDKN3, CDYL2, CEP55, CST1, DLGAP5, E2F6, ESPL1, EZH2, FASTKD1, FOXM1, GEN1, GLRA2, GYLTL1B, HIST1H3B, HMMR, IFNA1/IFNA13, IL1RAPL2, ITM2C, KIAA1210, KIF11, KIF14, KIF18B, KIF20A, KIF2C, KPNA2, MAGEL2, MKI67, MYBL2, NCAPG, NCL, NIPA2, NUF2, PAGE4, PBK, PRB4, PTH2R, PTTG1, RRM2, SPC25, TET1, THY1, TK1, TPX2, TROAP, TUBB2B, UBE2C
	interphase of tumor cell lines (*p* = 1.17E-03)<br>molecules: CCNA2, CDCA2, CDKN3, EZH2, FOXM1, KIF11, MYBL2
	non small cell lung adenocarcinoma (*p* = 1.28E-03)<br>molecules: CCNA2, CDC20, CDCA2, FOXM1, PRSS2, RRM2, TK1, UBE2C
	gastrointestinal carcinoma (*p* = 1.49E-03)<br>molecules: BLM, CCNA2, CDYL2, CST1, DLGAP5, E2F6, EZH2, FASTKD1, GYLTL1B, HMMR, IFNA1/IFNA13, IL1RAPL2, KIAA1210, KIF14, KIF18B, KPNA2, MAGEL2, MKI67, NCAPG, NCL, NIPA2, NUF2, PRB4, PTH2R, PTTG1, RRM2, SPC25, TET1, THY1, TK1, UBE2C<br>metastatic colorectal cancer (*p* = 1.77E-03)
	molecules: DLGAP5, KIF20A, NCAPG, PBK, RRM2<br>mammary tumor (*p* = 1.87E-03)
	molecules: CCNA2, CDC20, EZH2, FOXM1, HMMR, KIF18B, KIF2C, KPNA2, MKI67, MYBL2, NCL, PTTG1, RRM2, TET1, TK1, UBE2C
	G1 phase of tumor cell lines (*p* = 1.97E-03)<br>molecules: CDCA2, CDKN3, EZH2, FOXM1, MYBL2
	glioma (*p* = 2.20E-03)<br>molecules: CDKN3, EZH2, HIST1H3B, KPNA2, MKI67, PTTG1, RRM2, UBE2C
	large-cell lymphoma (*p* = 2.61E-03)<br>molecules: EZH2, HIST1H3B, RRM2, TAF4B, TUBB2B
	malignant neoplasm of pelvis (*p* = 2.62E-03)<br>molecules: CCNA2, CDC20, CEP55, E2F6, ESPL1, EZH2, FOXM1, GEN1, HMMR, IL1RAPL2, ITM2C, KIAA1210, KIF11, KIF14, KIF2C, KPNA2, MKI67, MYBL2, NCAPG, PAGE4, PRB4, PTTG1, RRM2, SPC25, TK1, TPX2, TUBB2B, UBE2C
	arrest in cell cycle progression (*p* = 2.82E-03)<br>molecules: CCNA2, CDKN3, EZH2, FOXM1, RNF4
	bladder cancer (*p* = 2.82E-03)<br>molecules: EZH2, IFNA1/IFNA13, RRM2, TROAP, UBE2C
	brain tumor (*p* = 3.61E-03)<br>molecules: CDKN3, EZH2, HIST1H3B, KPNA2, MKI67, PTTG1, RRM2, UBE2C
	colorectal carcinoma (*p* = 3.81E-03)<br>molecules: BLM, CCNA2, CDYL2, CST1, DLGAP5, E2F6, EZH2, FASTKD1, GYLTL1B, HMMR, IFNA1/IFNA13, IL1RAPL2, KIAA1210, KIF14, KIF18B, KPNA2, MAGEL2, MKI67, NCAPG, NCL, NIPA2, NUF2, PRB4, PTH2R, SPC25, TET1, TK1, UBE2C

(*continued*)

	lung cancer (*p* = 4.17E-03)<br>molecules: CCNA2, CDC20, CDCA2, EZH2, FOXM1, HIST1H3B, PRSS2, RRM2, TK1, UBE2C, UBE2NL<br><br>astrocytoma (*p* = 4.19E-03)<br>molecules: CDKN3, EZH2, KPNA2, MKI67, PTTG1, RRM2, UBE2C<br><br>lymphohematopoietic cancer (*p* = 4.31E-03)<br>molecules: CDC20, EZH2, FOXM1, HIST1H3B, KIF2C, KPNA2, MKI67, PRSS2, PSORS1C2, RRM2, TAF4B, TET1, TUBB2B, UBE2C<br><br>hematological neoplasia (*p* = 4.39E-03)<br>molecules: CDC20, EZH2, FOXM1, HIST1H3B, KIF2C, KPNA2, MKI67, PRSS2, PSORS1C2, RRM2, TAF4B, TET1, TUBB2B, UBE2C<br><br>carcinoma in lung (*p* = 4.67E-03)<br>molecules: CCNA2, CDC20, CDCA2, FOXM1, HIST1H3B, PRSS2, RRM2, TK1, UBE2C, UBE2NL<br><br>uterine cancer (*p* = 5.78E-03)<br>molecules: CCNA2, CDC20, CEP55, ESPL1, EZH2, FOXM1, GEN1, HMMR, ITM2C, KIAA1210, KIF11, KIF14, KIF2C, KPNA2, MKI67, MYBL2, NCAPG, PAGE4, PRB4, PTTG1, RRM2, SPC25, TK1, TPX2, TUBB2B, UBE2C<br><br>upper gastrointestinal tract cancer (*p* = 8.04E-03)<br>molecules: CDX1, EZH2, FOXM1, GLRA2, MKI67, PTTG1, RRM2, THY1, UBE2C<br><br>breast cancer (*p* = 8.69E-03)<br>molecules: CDC20, EZH2, FOXM1, HMMR, KIF18B, KPNA2, MKI67, MYBL2, NCL, PTTG1, RRM2, TET1, TK1, UBE2C

Supplementary Table S5. ABSOLUTE estimates of purity and ploidy. We employed the ABSOLUTE algorithm to estimate both ploidy and purity of samples in the training set. The table displays the output of the algorithm ABSOLUTE.summarize.

array	sample	call status	purity	ploidy	Genome doublings	delta	Coverage for 80% power	Cancer DNA fraction	Subclonal genome fraction	tau	E_CR
152T	152T	low purity	NA	NA	NA	NA	NA	NA	NA	NA	NA
149T	149T	called	0.43	3.99	0	0.15	34	0.6	0.02	3.971621209	0
158T	158T	called	0.24	4.37	0	0.09	58	0.4	0	4.278956914	0
143T	143T	called	0.19	2.11	0	0.1	56	0.2	0	2.076247313	0
139T	139T	called	0.4	2.02	0	0.2	21	0.4	0.03	2.001124491	0
103T	103T	called	0.21	2.04	0	0.11	51	0.21	0.01	2.108279617	0
92T	92T	called	0.2	4.44	0	0.08	67	0.36	0.01	4.474294799	0
156T	156T	called	0.17	1.72	0	0.09	63	0.15	0.01	1.755721731	0
148T	148T	called	0.19	2.33	0	0.09	59	0.21	0.01	2.400968667	0
145T	145T	called	0.17	2.75	0	0.08	68	0.22	0	2.734291266	0
157T	157T	called	0.21	4.99	0	0.08	69	0.4	0.02	4.675873423	0
120T	120T	called	0.16	1.94	0	0.08	67	0.16	0.01	1.988575466	0
126T	126T	called	0.19	3.63	0	0.08	65	0.3	0	3.618382836	0
131T	131T	called	0.18	2.12	0	0.09	60	0.19	0.01	2.115452618	0
109T	109T	called	0.24	5.86	0	0.08	67	0.48	0.01	5.725772162	0
137T	137T	called	0.21	5.26	0	0.08	68	0.42	0.02	4.872997584	0
159T	159T	called	0.17	2.44	0	0.08	65	0.2	0.01	2.388252884	0
124T	124T	called	0.22	4.6	0	0.09	62	0.4	0.03	4.429964011	0
118T	118T	called	0.18	3.1	0	0.08	67	0.25	0	3.107149166	0
125T	125T	called	0.24	6.06	0	0.08	67	0.49	0.04	5.789412538	0
114T	114T	called	0.17	2.55	0	0.08	67	0.21	0.01	2.584035313	0
119T	119T	called	0.2	2.73	0	0.09	59	0.25	0.04	2.728373486	0
117T	117T	Called	0.21	3.99	0	0.09	64	0.34	0.03	3.634842266	0

Supplementary Table S6. Threshold values for incorporating associations in the models. Pairwise associations were filtered with different thresholds of log-likelihood ratios and correlation coefficients according to the levels and types of effectors.

level	type	log odds ratio	*p*-value	correlation coefficient
1	*cis*-acting segment CNV	0.8	1	0.3
2	*trans*-acting segment CNV	2	1	0.5
2	DNA methylation	2	1	0.3
3	*trans*-acting segment CNV	2	1	0.5
3	DNA methylation	2	1	0.3

Supplementary Table S7. FDR measurements for pairwise associations and validation tests. For pairwise associations, we constructed a null model by computing pairwise association scores with randomly permuted data, calculated the expected number of significant pairs arising from the null model, and counted the ratio of this false discovery number and the number of significant pairs from the observed data. To calculate the FDR of validated modules, we randomly assigned genes to modules of the same sizes and counted the fraction of randomized modules passing the validation tests.

1. Pairwise associations

Pairwise 1 - Intra-segment CNV
Pairwise 2 - Inter-Segment CNV
Pairwise 3 - Methylation

	Observed	Expected	FDR
Pairwise 1	2,307	473.5	0.21
Pairwise 2	2,426	1,124	0.46
Pairwise 3	146,186	109,640	0.75

2. Validation tests

Test 1 - Coherance of Target Genes
Test 2 - Prognostic Power of Association Modules: Distribution of Cox Coefficients
Test 3 - Prognostic Power of Association Modules: Kaplan-Meier Log-Rank Test

	Observed	Expected	FDR
Test 1	19	1.295	0.0682
Test 2	11	3.089	0.2808
Test 3	3	0.5697	0.1899
All	3	0	< 0.002
